# Origin and Evolution of Key Enzymes in the Anammox Pathway Revisited

**DOI:** 10.1093/gbe/evaf244

**Published:** 2025-12-18

**Authors:** Emil Hägglund, Alejandro Jiménez-González, Erik Hagström, Patrik Björkholm, Lionel Guy, Siv G E Andersson

**Affiliations:** Molecular Evolution, Department of Cell and Molecular Biology, Science for Life Laboratory, Biomedical Centre, Uppsala University, Uppsala 752 36, Sweden; Molecular Evolution, Department of Cell and Molecular Biology, Science for Life Laboratory, Biomedical Centre, Uppsala University, Uppsala 752 36, Sweden; Molecular Evolution, Department of Cell and Molecular Biology, Science for Life Laboratory, Biomedical Centre, Uppsala University, Uppsala 752 36, Sweden; Molecular Evolution, Department of Cell and Molecular Biology, Science for Life Laboratory, Biomedical Centre, Uppsala University, Uppsala 752 36, Sweden; Department of Medical Biochemistry and Microbiology, Science for Life Laboratory, Biomedical Centre, Uppsala University, Uppsala, Sweden; Molecular Evolution, Department of Cell and Molecular Biology, Science for Life Laboratory, Biomedical Centre, Uppsala University, Uppsala 752 36, Sweden

**Keywords:** anaerobic ammonium oxidation, anamoxosome, hydrazine synthase, hydrazine dehydrogenase, hydroxylamine oxidoreductase

## Abstract

Anaerobic ammonium oxidizing bacteria in the class “*Candidatus* Brocadiia” in the *Planctomycetota* are the only known group of bacteria capable of producing energy by coupling the oxidation of ammonium to the reduction of nitrite within a unique bacterial organelle called the anammoxosome. Due to the lack of homologs in other species, it is hypothesized that the key enzyme in this process, the hydrazine synthase complex, originated by de novo birth. We performed extensive searches for proteins that exhibited similarity in sequence and structure to the hydrazine synthase subunits and identified distantly related homologs in anaerobic bacteria from the phyla *Planctomycetota* and *Desulfobacterota*. However, key residues of importance for the enzymatic function were not conserved, rejecting the hypothesis that the identified genes represent previously unrecognized anammox bacteria. Phylogenetic analyses indicate that the anammox pathway has been assembled from genes acquired by horizontal gene transfer from a variety of anaerobic bacteria. The ancestral states of enzymes in the hydroxylamine oxidoreductase family were inferred, and transitions between reductive and oxidative forms of the enzymes were mapped onto the phylogenetic tree. Finally, it is shown that the signal sequences of key enzymes in the anammox pathway are able to transport a reporter gene into the periplasm of *Escherichia coli* cells. In conclusion, our findings suggest that the hydrazine synthase complex has evolved from already existing heme-binding periplasmic proteins and that the anammoxosome has an endogenous origin.

Significance The anammoxosome is the only described energy-producing organelle in bacteria, but the origin of the organelle and its key enzyme, the hydrazine synthase complex, is unknown. By using sequence-based and structure-based search methods, this study uncovers homologs to the hydrazine synthase subunits in distantly related anaerobic bacterial species. Phylogenetic and experimental studies indicate that the enzymes in the anammox pathway evolved from heme-binding proteins targeted to the periplasmic space, consistent with theories suggesting that the anammoxosome evolved from invaginations of the cytoplasmic membrane.

## Introduction

The anaerobic ammonium oxidation (anammox) bacteria are key organisms in the global nitrogen cycle. They are the only known organisms capable of producing energy by oxidizing ammonium with nitrate to form dinitrogen gas, and it is estimated that 30% to 50% of the N_2_ production in the oceans is derived from this process (Lam and Kuypers 2011; Oshiki et al. 2016; Stein and Klotz 2016). Based on thermodynamic calculations, the anammox pathway was predicted as a potential microbial energy route already in 1977 (Broda 1977; Oren 2015), but it was not until the 1990s that the anammox process was observed (Van de Graaf et al. 1990; Mulder 1995; van de Graaf et al. 1995; Kuenen 2008). Although the reaction mechanisms have since been determined and are well understood, the origin of this unique bioenergetic system is still enigmatic.

The anammox bacteria are classified into the class “*Candidatus* Brocadiia” in the phylum *Planctomycetota* (Oren 2022), recognized for their complex cellular architectures. The anammox reaction takes place inside an organelle called the anammoxosome, which occupies 50% to 70% of the bacterial cell volume and is the only energy-producing organelle described in bacteria so far (Neumann et al. 2014; de Almeida et al. 2015). The membranes of the anammoxosome are uniquely enriched with ladderane phospholipids that are thought to control the permeability of the membranes (Sinninghe Damsté et al. 2002; Kartal et al. 2013; Moss et al. 2018). It has been observed that the anammoxosome membrane is associated with the chromosome during the cell division process (van Niftrik et al. 2004; Fuerst 2005; Van Niftrik et al. 2009).

**Fig. 1.**
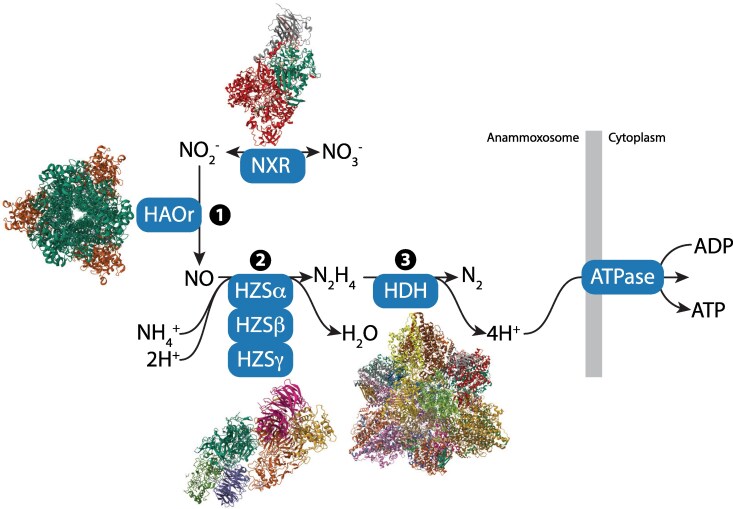
Overview of the anaerobic ammonium oxidation pathway. Nitrite reduction to nitric oxide is performed by a conserved nitrite reductase of the hydroxylamine oxidoreductase (HAO)-like family. Nitric oxide and ammonia are used to form hydrazine by the hydrazine synthase (HZS). Next, hydrazine is oxidized to dinitrogen gas by hydrazine dehydrogenase (HDH). The resulting protein motive force drives the synthesis of ATP by F-ATP synthase attached to the anammoxosome membrane. Nitrite can also be oxidized to nitrate by a nitrite oxidoreductase complex (NXR). Protein structure cartoons of the multidomain enzymes involved in the pathway are shown.

The anammox pathway is a three-step process of redox reactions that involves the formation and oxidation of hydrazine (N_2_H_4_), resulting in a proton motive force that drives ATP synthesis (Fig. 1). Hydrazine is an extremely reactive and highly toxic compound. The first step in the anammox reaction is the reduction of nitrite (NO_2_^−^) to nitric oxide (NO). Initially, it was thought that this reaction was catalyzed by nitrite reductase, NirS (in “*Candidatus* Scalindua brodae”) and NirK (in “*Candidatus* Jettenia caeni”). However, not all members of the anammox bacteria have these proteins. More recently, it has been shown that a highly conserved octaheme cytochrome *c* protein of the hydroxylamine oxidoreductase (HAO)-like protein family in “*Candidatus* Kuenenia stuttgartiensis” (HAOr) can catalyze this first reaction (Ferousi et al. 2021).

The second step involves the synthesis of hydrazine from NO and ammonium (NH_4_^+^). This step is performed by the hydrazine synthase complex (HZS), a soluble and biochemically unique enzyme consisting of a dimer of three subunits (α, β, γ). The structure of the HZS complex shows that the α-subunit consists of an N-terminal six-bladed β-propeller, a middle domain containing the heme αI active site, and a C-terminal domain with an atypical bis-histidine-coordinated heme *c* site (heme αII) (Dietl et al. 2015). The γ-subunit consists of an N-terminal domain, which contains the heme γI active site, and a C-terminal domain with the bis-histidine-coordinated heme *c* site (heme γII), thought to be involved in electron transfer. The β-subunit is a seven-bladed β-propeller with an insertion in the sixth propeller blade but lacks any known active site. The protein structure has also provided insights into the mechanism whereby hydrazine is generated and transported within the HZS complex. It is suggested that NO is first reduced to hydroxylamine at the active site of the γ-subunit (heme γI), after which it diffuses through a tunnel to the active site of the α-subunit (heme αI), where it is condensed with ammonia to generate hydrazine (Dietl et al. 2015). The tunnel opens to the surface halfway between the two sites, allowing hydrazine to exit to the surface. Structurally, the γ-subunit of the HZS shows similarity to that of diheme cytochrome *c* peroxidases (CCPs), and it has been noted that hydroxylamine and hydrogen peroxide have the same isoelectric point (Dietl et al. 2015).

The third step of the anammox pathway is the oxidation of hydrazine to dinitrogen gas. Similar to HAOr, the hydrazine dehydrogenase (HDH) is also a member of the HAO-like family (Maalcke et al. 2016). Unlike the membrane-associated mitochondrial cytochrome complexes, HDH is not linked to the anammoxosome membrane but is a soluble protein within the organelle (de Almeida et al. 2015). Crystal structures and cryo-electron microscopy structures suggest that HDH consists of 24 copies of a single subunit arranged as an octamer of trimers (α_3_)_8_ (Akram et al. 2019). Each monomer contains eight heme groups, which form a ring-like relay system for electron transfer. Remarkably, the complex contains as many as 192 heme groups that serve as an extended electron transfer network with 24 exit sites, facilitating interactions with electron acceptor proteins. Although the identities of the electron acceptors are not known, they are thought to be small 15-kDa cytochrome *c*-like proteins (Akram et al. 2019). The four electrons released from the hydrazine oxidation process are transferred from the electron acceptors to the cytochrome *bc*1 complexes, resulting in the import of protons to the anammoxosome and generating a proton motive force that drives the synthesis of ATP by the canonical F-ATP synthase.

The nitrite oxidoreductase (NXR) complex is another key enzymatic complex that can catalyze the oxidation of nitrite to nitrate and the reduction of nitrate to nitrite (Chicano et al. 2021). The complex is localized inside the anammoxosome (de Almeida et al. 2015), and forms tubular superstructures consisting of heterotrimers of the NXR-ABC subunits connected by the tubule-inducing and heme-containing protein NXR-T (Chicano et al. 2021). The subunits in the NXR complex are encoded by a cluster of 15 genes in the “*Ca.* Kuenenia stuttgartiensis” genome (Chicano et al. 2021).

The anammox reaction depends on the availability of ammonium produced by anaerobic respiration and nitrite produced by aerobic ammonia oxidation (Sonthiphand et al. 2014). However, NO can also be used in the absence of nitrite (Hu et al. 2019). Consequently, the anammox bacteria are adapted to habitats at the interface of aerobic-anaerobic environments, such as the oxygen minimum zones in the oceans and the nitrate-ammonium transition zones in subsurface sediments (Zhao et al. 2020). Members of the family “*Ca*. Brocadiaceae”, which comprise the genera “*Ca*. Brocadia”, “*Ca*. Jettenia”, and “*Ca.* Kuenenia”, are commonly observed in wastewater treatment plants, and members of the family “*Ca.* Scalinduaceae”, which comprise the genus “*Ca*. Scalindua”, are identified in marine environments. Recently, a novel family named “*Candidatus* Anammoxibacteraceae” was recognized in biofilms inside a subsea road tunnel in Norway (Suarez et al. 2022). In addition, a deeply diverging family named “*Candidatus* Bathyanammoxibiaceae” was discovered in marine sediments from the Arctic Mid-Ocean Ridge (Zhao et al. 2022). This novel family of anammox bacteria might also be present in freshwater sediments and soil habitats, as inferred from 16S rRNA analyses (Zhao et al. 2022).

Currently, the public databases hold more than 100 metagenome-assembled genomes (MAGs) and single-cell amplified genomes of anammox bacteria obtained from environmental surveys (Speth et al. 2017; Zhao et al. 2020) and wastewater bioreactors (Speth et al. 2016; Park et al. 2017). However, no anammox bacterial cells have yet been obtained in pure culture, which has hampered attempts to determine their cell physiology and obtain high-quality, closed genomes. As an alternative to pure cultures, enrichment cultures have been established in bioreactor systems, and closed genomes and draft genomes in various stages of completion have been assembled from “*Ca*. Kuenenia”, “*Ca*. Brocadia”, *Ca*. Jettenia and “*Ca*. Scalindua” (Strous et al. 2006; Hira et al. 2012; Speth et al. 2012; Ali et al. 2015; Oshiki et al. 2015; Speth et al. 2015; Ali et al. 2016; Narita et al. 2017; Oshiki et al. 2017; Park et al. 2017; Frank et al. 2018; Ali et al. 2020; Ding and Adrian 2020; Okubo et al. 2021). The enrichment cultures also contained co-cultivated bacteria from the phyla *Bacteroidota* and *Chloroflexota* (Okubo et al. 2021; Oshiki et al. 2022), which may be involved in the supply of NO (Okubo et al. 2021), exchange of amino acids and vitamins, and/or in the degradation of polysaccharides (Oshiki et al. 2022).

Despite the availability of genomic data, resolved protein structures and knowledge about the reaction mechanisms of the key enzymes involved in the anammox reaction, there is no consensus on when and how these enzymes originated. One hypothesis, inferred from a phylogenomic and molecular dating approach, suggests that the anammox bacteria originated during the Great Oxidation Event (GOE) when oxygen levels increased, and nitrite became available (Liao et al. 2022). It has been suggested that HZS and HDH originated first, while the other enzymes in the pathway were acquired later via horizontal gene transfer (Hu et al. 2019). For example, it has been shown that the NXRs in the anammox bacteria are phylogenetically related to the NXRs in *Nitrospina* and *Nitrospira* (Lücker et al. 2010, 2013; Kitzinger et al. 2018). Other suggested donors of the horizontally acquired genes belong to the *Firmicutes*, *Proteobacteria*, and *Euryarchaeota* (Liao et al. 2022).

However, the origin of the HZS complex, which is thought to have been the prime event in the subsequent cascade of evolutionary changes, remains elusive. One hypothesis is that this complex originated by de novo birth in the anammox bacteria since no homologs with significant sequence similarity could be identified (Liao et al. 2022). More recently, homologs to the HZS complex were identified in another lineage of the *Planctomycetota*, leading to the suggestion that anammox bacteria are more widespread than previously thought (Suarez et al. 2023). To understand how this dramatic metabolic transition to anaerobic ammonium oxidation occurred, we have re-examined the evolutionary relationships of key enzymes in the anammox pathway. Here, we present the results of our phylogenetic studies and propose a model for the origin and evolution of the key enzymes involved in the anammox pathway.

## Results

### Genome Datasets that Represent the Diversity of the Anammox Bacteria

We first compiled two datasets of genomes to represent the genetic diversity of the anammox bacteria. The first dataset consisted of complete or near-complete genomes from bacteria cultivated in laboratory bioreactor enrichment cultures. This dataset, referred to as the high-quality genome dataset, contained a total of eight genomes. It included four closed and two draft genomes (3.3 to 4.1 Mb) from members of the family “*Ca*. Brocadiaceae” as well as two draft genomes (4.7 to 4.8 Mb, 4 to 47 contigs) from “*Ca*. Scalinduaceae” (Table S1).

The second dataset consisted of MAGs obtained from environmental bacteria to broaden the phylogenetic diversity. We started out by extracting all MAGs classified as “*Ca*. Brocadiia” according to the NCBI taxonomy (2022-11-30) and/or the GTDB v207 taxonomy and used the RNA polymerase subunit beta (RpoB) as a marker protein to screen for a representative set of MAGs (Fig. S1a). After removing MAGs that lacked the *rpoB* gene as well as MAGs that were misclassified as “*Ca*. Brocadiia”, we inferred a maximum likelihood phylogeny based on the RpoB protein sequences from the remaining 150 MAGs (Fig. S1b). Of these, 137 MAGs clustered within the clade containing “*Ca*. Brocadiaceae” and “*Ca*. Scalinduaceae”, while 13 MAGs were placed outside this group. We selected a total of 12 MAGs that represented the diversity of the anammox bacteria and were composed of <200 contigs, including four MAGs from “*Ca*. Brocadiaceae”, four MAGs from “*Ca*. Scalinduaceae”, and four MAGs from the early diverging lineages. The 12 MAGs were added to the 8 high-quality genomes to generate an extended dataset of 20 genomes (Table S1).

**Fig. 2.**
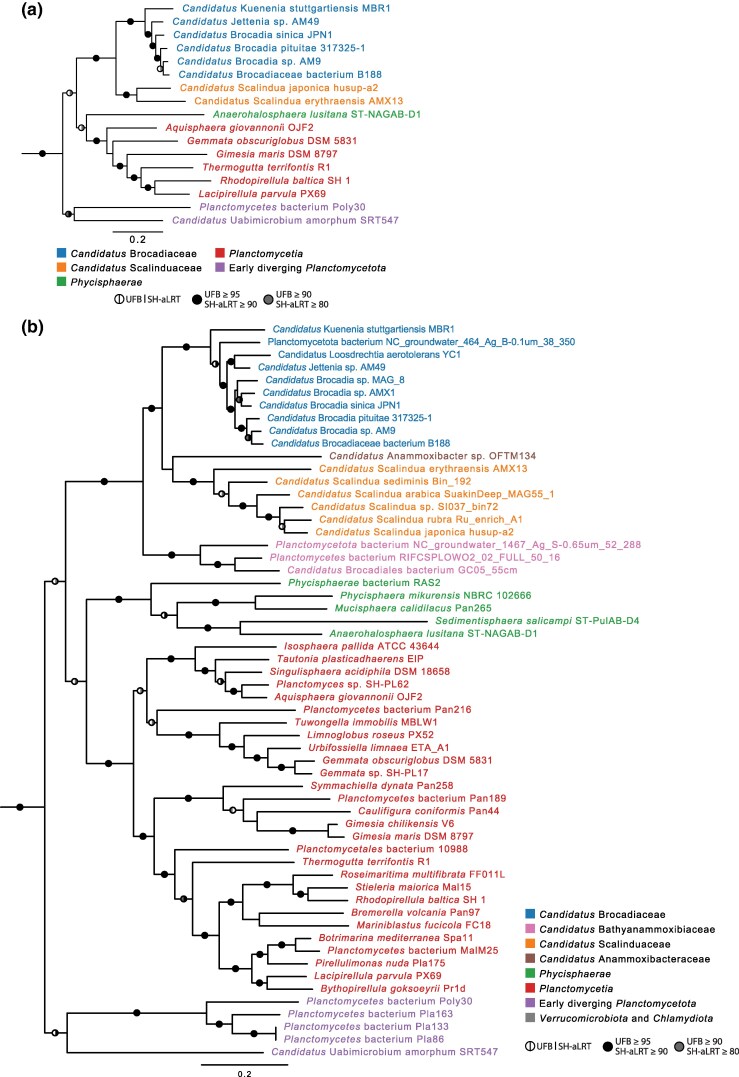
Maximum likelihood phylogenies of RNA polymerase subunit beta. The phylogenies were inferred from RNA polymerase subunit beta (RpoB) sequences encoded by genes in the a) high-quality and b) extended genome datasets from the class “*Candidatus* Brocadiia”. Phylogenies were calculated with IQ-Tree v2.2.0 under the LG + F + G substitution model and rooted with taxa from the PVC superphylum. Ultrafast bootstrap (UFB) ≥ 95 and SH-aLRT ≥ 90 are marked with black semicircles and UFB ≥ 90 and SH-aLRT ≥ 80 are marked with gray semicircles on the branches. The complete phylogenies of the high-quality and extended genome datasets are shown in Figs. S1c and S1d, respectively.

Two additional maximum likelihood phylogenies were inferred from the RpoB protein sequences encoded by the high quality (Fig. 2a; Fig. S1c) and the extended genome datasets (Fig. 2b; Fig. S1d), along with outgroup genomes from the PVC phylum. The RpoB phylogeny confirmed that “*Candidatus* Anammoxibacter sp. OFTM134” diverged early within the clade encompassing “*Ca*. Brocadiaceae” and “*Ca*. Scalinduaceae”, and that “*Ca*. Bathyanammoxibiaceae” diverged prior to “*Ca*. Brocadiaceae” and “*Ca*. Scalinduaceae” with 100% bootstrap support. Thus, the selected genomes recovered the known diversity of the anammox bacteria, as described in the literature. We reasoned that including more MAGs of lower sequence quality, lower completeness and/or higher levels of contamination would risk introducing noise into the analyses without providing any added value.

### Identification of Homologous Proteins to the Anammox Enzymes

**Fig. 3.**
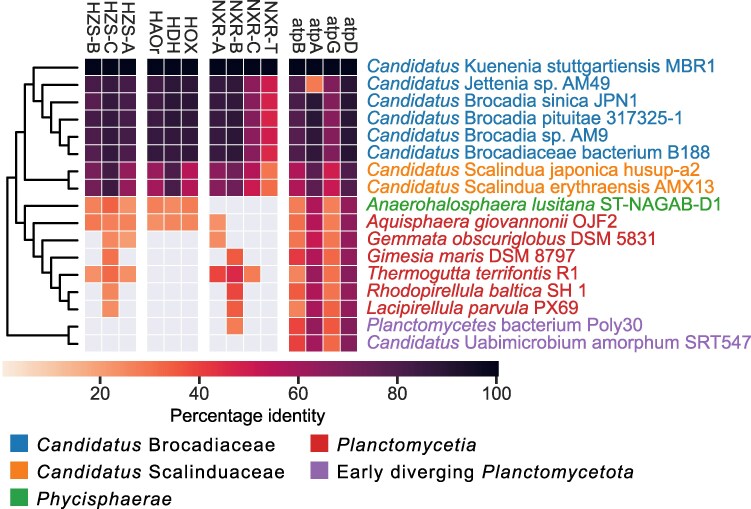
Phyletic distribution pattern of key enzymes in the anammox pathway. The pattern is shown for taxa in the high-quality genome dataset of the anammox bacteria and representative species from *Planctomycetota*. Species are colored according to taxonomic affiliation. Colors in boxes show the percentage identity of the alignment in the local BLAST searches. HZS-A, HZS-B, and HZS-C = subunits of hydrazine synthase complex; HAOr, HOX = enzymes in the hydroxylamine oxidoreductase family; HDH = hydrazine dehydrogenase; NXR-A, NXR-B, NXR-C, NXR-T = subunits of the nitrite oxidoreductase complex; ATPA, ATPB, ATPD, ATPG = subunits of the F-ATP synthase complex. The phyletic distribution pattern of the same set of enzymes in the extended genome dataset is shown in Fig. S2.

Proteins in the hydrazine synthase (HZS), hydroxylamine oxidoreductase (HAO), and NXR complexes in “*Ca*. Kuenenia stuttgartiensis” were used as queries in a homology search against the proteomes of the selected taxa in “*Ca*. Brocadiia” along with the proteomes of the selected outgroup species in the PVC phylum (Fig. 3; Fig. S2; Table S2). A local BLAST search showed protein sequence identity values ranging from 50% to 90% for hits to the anammox bacteria and 25% to 30% for hits to the other species within the PVC superphylum. Importantly, no species other than the anammox bacteria within our representative set of species from the *Planctomycetota* contained homologs to all proteins in the HZS, NXR, and HAO families. However, *Anaerohalosphaera lusitana* and *Aquisphaera giovannonii* contained proteins with sequence similarity to the HZS and HAO-like proteins, and *Thermogutta terrifontis* contained homologs to protein subunits in the HZS and the NXR complexes.

We repeated the analyses to also search for homologs to the HZS complex outside the PVC phylum, including all representative species in GTDB v207. The search resulted in 300 to 1,000 hits with bit scores of 80 to 200 (Fig. S3a), of which we examined a smaller subset of hits present in RefSeq (Fig. S3b, Table S3). More than 20 hits were obtained in the RefSeq database when the α-subunit was used as the query, no hits were found when only the β-subunit was used as the query, and only five hits were obtained to the γ-subunit. However, we obtained more than 100 hits when using a protein consisting of fused β- and γ-subunits as the query. This result suggests that the putative homologs to the β- and γ-subunits of the HZS complex outside the anammox bacteria may also be fused, as in “*Ca.* Scalindua japonica”.

To study the relationships of the putative homologs to the HZS complex, we inferred maximum likelihood phylogenies based on the α- and the βγ-subunits and their putative homologs in other species, as defined above. The tree obtained from the α-subunits showed that the homologs could be sorted into four groups, two of which contained members of the *Planctomycetota*, including *Aquisphaera* spp. and *T. terrifontis* (Fig. S4a, Table S4a). The tree inferred from the putative homologs of the βγ-subunits contained many more taxa from diverse phyla, not only from *Planctomycetota* but also species affiliated with *Bacteroidota* and *Bacillus* (Fig. S4b, Table S4b). We reasoned that genes coding for protein subunits that share a common origin with the HZS complex would most likely contain genes for both the α- and βγ-subunits located within the same operon. Interestingly, a subset of genomes contained a gene for a homolog to the fused βγ-subunit located in the vicinity of the gene for the homolog to the α-subunit (Fig. S4c) and vice versa (Fig. S4d).

**Fig. 4.**
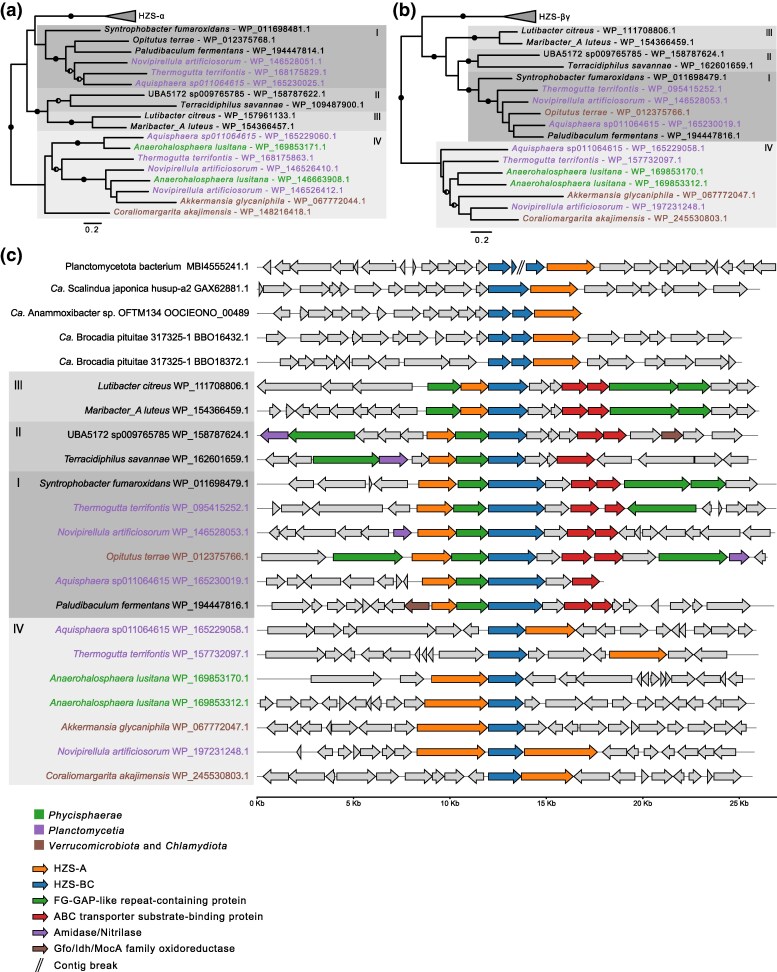
Maximum likelihood phylogenies of hydrazine synthase subunits. The phylogenies were inferred from the a) α-subunit and b) βγ-subunits of hydrazine synthase in the anammox bacteria and their putative homologs encoded by co-localized genes in other species. c) Gene order structures for genomic regions containing the genes included in the phylogenies. Clades in the trees are marked with I, II, III, and IV. The phylogenies are midpoint rooted and ultrafast bootstrap support ≥ 95 and SH-aLRT support ≥ 80 are indicated with semicircles. The complete phylogenies are shown in Fig. S5a and b.

Next, we selected a representative set of genomes in which the genes for homologs to the α- and βγ-subunits were co-localized. The phylogenetic trees inferred from the protein alignments suggested that the homologs encoded by co-located genes could be sorted into four clades, which we, for simplicity, have numbered clade I to clade IV (Fig. 4a and b; Fig. S5ab; Tables S5ab). Both clade I and clade IV contained three species that belong to the *Planctomycetota* (*Aquisphaera giovannonii, T. terrifontis,* and *Novipirellula artificiosorum).* Notably, *A. lusitana,* which belongs to *Phycisphaera*, also contained two copies of these gene clusters, although both clusters belonged to clade IV. Homologs encoded by co-localized genes were also detected in a few species outside the PVC phylum; in clade I, these comprised *Syntrophobacter fumaroxidans* and *Paludibaculum fermentans*. The sequence identities of the homologs to the HZS subunits were estimated from the trimmed alignment to range from 23% to 67% within the clades of the α-subunit (Fig. S5c) and from 35% to 74% within clades of the βγ-subunit (Fig. S5d).

We also examined the order of genes for the homologs to the HZS subunits within each of the four clades (Fig. 4c). In the anammox bacteria, the genes for the β- and γ-subunits were located upstream of the gene for the α-subunit. The sequences of the two copies for HZS subunits in “*Ca.* Brocadiaceae” were identical. However, the surrounding gene order structures were not, and we therefore included both copies in the comparison. Unfortunately, the genes for HZS subunits in “*Ca*. Bathyanammoxibiaceae” were located on different contigs, which broke the operon structure. Nevertheless, it was evident that the β- and γ-subunits were encoded by two distinct genes, as in “*Ca*. Brocadiaceae”.

Interestingly, the converse organization of these genes was seen in taxa classified into clades I-III, such that the gene for the α-subunit was located upstream of the gene for the βγ-subunit (Fig. 4c). Genes for the α- and βγ-subunits were flanking a gene coding for an FG-GAP repeat-containing protein annotated as CRTAC1 family protein in members of clades I and II. The gene for the FG-GAP repeat protein was also identified in the taxa of clade III. However, it was located upstream, rather than downstream, of the gene for the putative homolog to the α-subunit in the HZS complex. Furthermore, two genes for ABC transporter substrate-binding proteins were identified downstream of this gene cluster. In clade IV, the order of the genes for the α- and βγ-subunits was taxa-specific, and no genes for FG-GAP repeat proteins or ABC transporters were identified in this gene neighborhood. Taken together, the co-localization of the genes for the putative homologs to the α- and βγ-subunits in genomes that belong to clades I–IV in the tree indicate that they may be part of the same complex, like the HZS subunits in the anammox bacteria.

### Predicted Structures of the Homologs to the HZS Subunits

We also performed searches for structural homologs to the α, β, and γ subunits of the HZS complex in “*Ca*. Kuenenia stuttgartiensis” (Table S6a) and to the fused βγ-subunit of “*Ca*. Scalindua japonica” (Table S6b) using the FoldSeek web server (van Kempen et al. 2024). Significant hits to the α-subunit were obtained to proteins in anammox bacteria (*E* < 10^−95^) and among the 50 top hits (*E* < 10^−35^) we recognized the homologs assigned to clade I in the phylogeny shown in (Fig. 4a) from *A. giovannonii, N. artificiosorum, T. terrifontis*, and *S. fumaroxidans*. In total, more than 600 hits were obtained (*E* < 10^−10^) most of which were described in the PDB database as “HZS_alpha domain-containing protein” or “cytochrome *c* domain-containing protein”. No proteins with another, experimentally confirmed function were among the top hits to the α-subunit of the HZS complex.

When the single β and γ-subunits were used as the query in the Foldseek search, the strongest hits were to proteins in the anammox bacteria (*E* < 10^−50^) and a few other members of the *Planctomycetota* (*E* < 10^−25^). As in the sequence-based searches for homologs, more and stronger hits were obtained when the fused βγ-subunit rather than the individual subunits were used as the query. However, the top hits in this search did not include the proteins encoded by genes located in close proximity to the genes for the homologs to the α-subunit in species assigned to clade I in the sequence-based phylogeny (Fig. 4b). Some of the putative homologs βγ-subunit were described as CCPs or cytochrome *c* domain-containing proteins (*E* < 10^−30^), suggesting that they are heme-binding proteins. Part of the query protein also showed structural similarity to YTVN and PQQ domains in a broad variety of bacteria, indicating a role in protein-protein interaction or protein complex assembly.

To obtain an estimate of the similarity in structure, we performed pairwise structure alignments between the α-subunit and the fused βγ-subunits of the HZS complex in “*Ca*. Kuenenia stuttgartiensis” and their homologs in other species for which predicted structures were available in the AlphaFold database (Fig. S6a; Table S7). Overall, the TM-scores were in the range of 0.6 to 0.9, confirming that they are similar in structure. The TM-scores were generally higher for the βγ-subunits (average 0.78) than for the α-subunits (average 0.67). The TM-scores were estimated to 0.66 to 0.67 for the structure-based alignments of the α-subunit and to 0.80 to 0.81 for the βγ-subunits to the homologous proteins in *S. fumaroxidans* and *Opitutus terrae* (Fig. S6b, Table S7). These scores represented the highest TM-scores among all proteins in the RefSeq database that displayed sequence similarity to HZS subunits in the anammox bacteria and were present in the AlphaFold database.

**Fig. 5.**
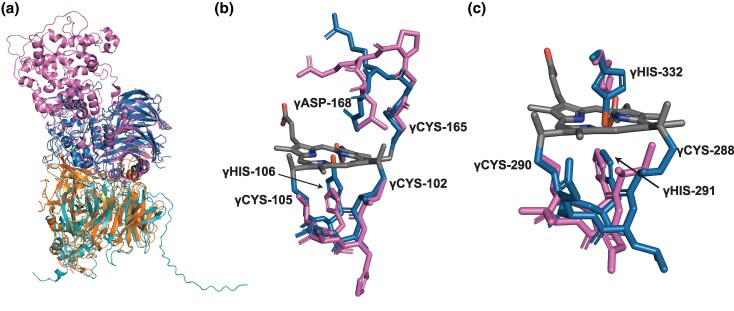
Structure-based comparison of hydrazine synthase subunits. a) Structural alignment of α- (orange) and βγ-subunits (blue) of hydrazine synthase (HZS) and predicted structure of the homologs from *Syntrophobacter fumaroxidans* (cyan, pink). b) Alignment of the binding pocket for heme γI from γ-subunit of HZS (blue) and the corresponding region of *S. fumaroxidans* (pink), and c) alignment of the binding pocket for heme γII from γ-subunit of HZS (blue) and the corresponding region of *S. fumaroxidans* (pink).

Based on a structure superposition of the α-subunits and βγ-subunits to the predicted structure of the homolog in *S. fumaroxidans* (Fig. 5a) and Multiple sequence alignments (MSAs) of the homologs the α-subunits and βγ-subunits (Fig. S7), we examined the conservation of key amino acid residues in the HZS subunits that are considered to be of importance for its function with a focus on the heme-containing active sites.

The α-subunit of “*Ca*. Kuenenia stuttgartiensis” contains a *c*-type heme (heme α1) and a bis-histidine-coordinated *c*-type heme (heme αII) (Dietl et al. 2015). While all homologs in the anammox bacteria and clades I-III contain the heme α1-site, members of clades II and III, and *O. terrae* in clade I, lack the heme αII-site. Members of the II and III clades also lack roughly the last one hundred amino acids of the protein, which explains their overall shorter lengths. The heme α1 differs from a canonical heme-*c* binding pocket in that αHis587 does not coordinate the heme iron, but a zinc ion which is jointly coordinated with αC303. The heme iron is instead coordinated by αTyr591. Interestingly, the protein alignment reveals a gap of two amino acids in all homologs from clades I-III at positions corresponding to αTyr591 and the nearby αSer590. The αC303, which is involved in coordination of the zinc ion in the α-subunit, is not conserved in any homologs. However, this residue is neither conserved in all anammox bacteria. For example, it is not present in members of “*Ca.* Scalinduaceae”. Consistently, the predicted structure of the homolog in *S. fumaroxidans* suggests that it contains the canonical heme-*c* binding pocket and thus differs from the α-subunit in this respect. The bis-His-coordinated heme site, heme αII, is only present in protein homologs from clade I. However, the αHis772, which coordinates the heme iron at the distal side in the α-subunit, is absent in these proteins.

The homologs to the βγ-subunits are more conserved in sequence and structure, and all contain the two canonical heme-binding motifs in their C-terminal ends. Interestingly, members of clade I contain an additional eight heme-binding motifs at their N-terminal ends that are classified as two cytochrome *c*554 domains, except *O. terrae,* which only contains four such motifs. The homologs in clade II have no additional heme-binding motifs, while the homologs in clade III have one extra motif. The β-subunit contains a seven-bladed β-propeller domain but no heme-binding motif. Similar to the β-subunit, the homologs contain an insertion in the sixth propeller blade, which forms a loop, suggested to be important for the reaction mechanism in HZS as it is placed in a tunnel connecting the γ-subunit and the α-subunit and proposed to modulate the transport between the two subunits. The loop contains a conserved glutamic acid coordinating a magnesium ion in the β-subunit. While the loop contains some residues also present in the homologs, the glutamic acid was not conserved.

The structure around heme γ1 is generally conserved between the γ-subunits in “*Ca*. Kuenenia stuttgartiensis” and the homologous heme in *S. fumaroxidans* (Fig. 5b). Heme γ1 is coordinated by γHis106 and is covalently bound to γCys102 and γCys105, and it also has a third, unique covalent bond to γCys165. The homologous proteins in clade I also contain a cysteine at this site, suggesting that they also may form a third covalent bond between the heme group and γCys165. The heme γII, which is thought to serve a role in electron transfer, is located in the same position in the γ-subunit in “*Ca*. Kuenenia stuttgartiensis” and its homologs in *S. fumaroxidans* (Fig. 5c). However, immediately upstream of the heme-binding motif, there is a stretch of 10 residues that are conserved in the γ-subunits of the anammox bacteria but absent from their homologs from the I-III clades, and immediately downstream of the motif, there is a stretch of six conserved residues in the homologs that are absent from the anammox bacteria. Cytochrome *c* peroxidase and MauG also contain two heme-binding motifs at similar positions. They have a Tryptophan residue that is important for their catalytic activity, which is placed at a position that corresponds to γHis144 in the γ-subunit. Interestingly, the homologs in clades I-III also have a conserved Tryptophan residue, rather than a Histidine residue at this position. These results suggest that although the protein homologs show sequence and structural similarity to the subunits of the HZS complex, the residues that are most important for its function are not conserved.

### Expansion and Diversification of Hydroxylamine Oxidoreductases

To learn more about the evolution of the hydroxylamine oxidoreductase (HAO)-like enzymes, we inferred a phylogeny based on proteins from the anammox bacteria and their homologs in RefSeq, as identified by a BLASTP search against the GTDB database (Table S8). The clades in the phylogeny that contained HAO-like proteins were named from HAO1-HAO10 according to Okubo et al. 2021, and the phylogeny was rooted with octaheme nitrite reductase (ONR) as an outgroup, according to (Soares et al. 2022).

**Fig. 6.**
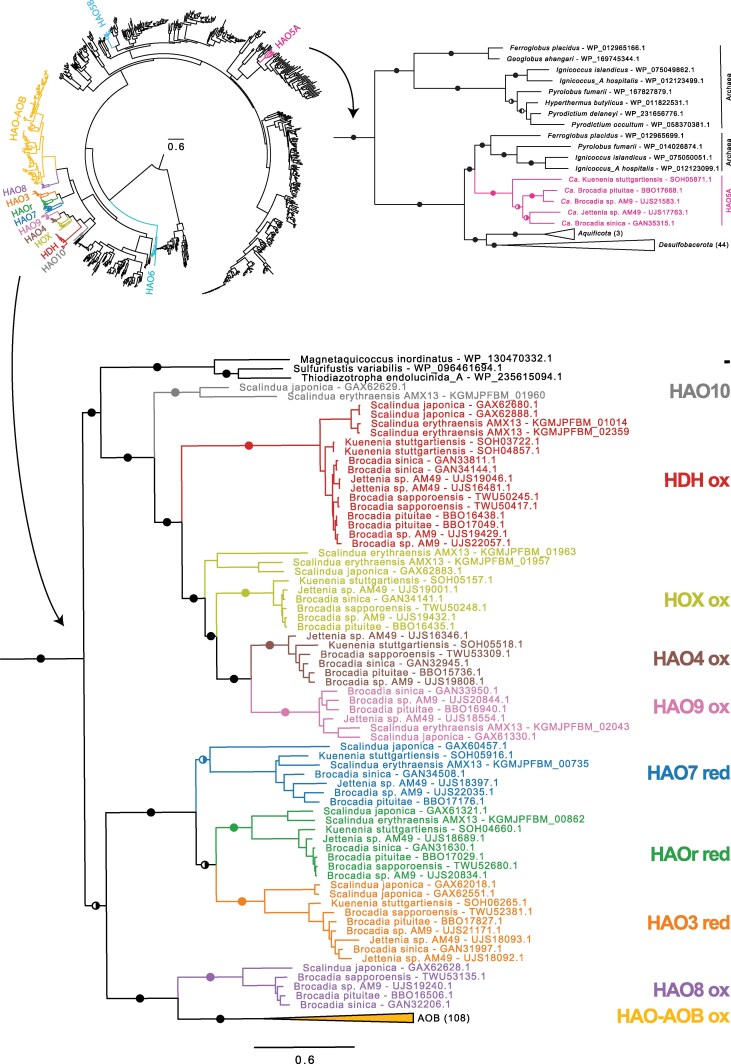
Maximum likelihood phylogeny of octaheme cytochrome *c* (OCC) proteins. The phylogeny was inferred from the hydroxylamine oxidoreductase (HAO)-like proteins encoded by genes in the high-quality genome dataset of the anammox bacteria and their homologs in other species. An overview of the phylogenetic relationship of all OCC proteins is shown, in which the two subsections show the relationship of a large clade of HAO-like proteins from the anammox bacteria together with HAO from aerobic ammonium oxidizing bacteria (AOB), and also HAO5A to other bacterial and archaeal taxa. The phylogenies were rooted with ONR as an outgroup and ultrafast bootstrap support ≥ 95 and SH-aLRT support ≥ 80 are indicated with semicircles. The complete phylogeny is shown in Fig. S8a.

The phylogenies inferred from the HAO proteins encoded by the high-quality (Fig. 6; Fig. S8a) and extended (Fig. S8b; Table S9) genome datasets showed that nine of the HAO-like enzymes in the anammox bacteria belonged to the same clade, here called the major clade, while another three enzymes represented single lineages that clustered elsewhere. The major clade could be further sorted into three subclades, one of which contained HAOr (HAO2), HAO3, and HAO7, another that contained HAO8, and a third subclade that contained HAO4, HAO9, HAO10, HDH, and HOX. The phylogenies confirmed that the HAO-like enzymes in the major clade originated prior to the diversification of the anammox bacteria. However, HAO4, HAO5A, and HAO6 could not be identified in “*Ca*. Scalindua,” and HAO10 was absent from “*Ca*. Brocadiaceae” and “*Ca.* Bathyanammoxibiaceae”. Furthermore, none of the HAO-like enzymes that clustered outside the major clade (HAO5a, HAO5b, and HAO6) contained representatives from *“Ca.* Bathyanammoxibiaceae”.

Interestingly, and as also observed in Okubo et al. 2021, the subclade that contained HAO8 formed a sister group to the HAO enzymes in aerobic ammonium oxidizing bacteria (AOB). Furthermore, the subclade that contained HOX and HDH clustered with a few bacterial species such as *Magnetoquicoccae inordinatus,* while HAO5A clustered with a few archaeal species such as *Ignococcus islandicus* and *Ferroglobus placidus*. The major clade as well as the three smaller subclades of HAO enzymes in the anammox bacteria were embedded within larger clades that contained many species in the phylum *Desulfobacterota*.

**Fig. 7.**
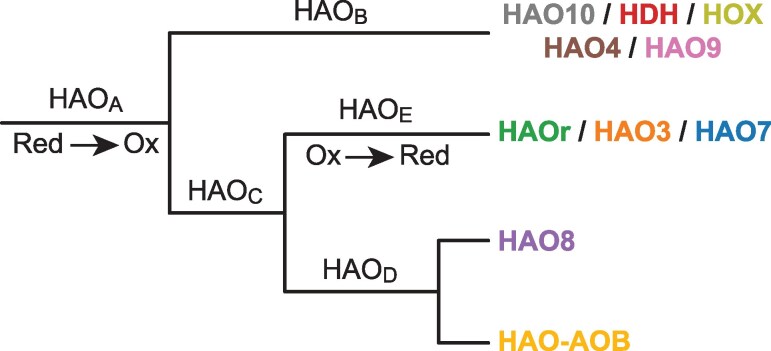
Schematic illustration of the evolution of the oxidative hydroxylamine oxidoreductase (HAO)-like proteins in the anammox bacteria. A schematic tree based on the results of the ancestral sequence reconstruction, which indicated a single transition from a reductive to an oxidative enzyme at the ancestral node HAO_A_, followed by the reversion to a reductive enzyme at the ancestral node HAO_E_. The complete phylogeny is shown in Fig. S8c.

Oxidative HAOs, such as HDH and HOX, contain a unique Tyrosine residue in one subunit in contrast to reductive HAOs, such as HAOr and HAO3 (Caranto et al. 2016; Ferousi et al. 2021). Using ancestral sequence reconstruction, we inferred the presence of the Tyrosine residue in the ancestral sequences (Fig. 7; Fig. S8c; Table S10 in the SciLifeLab repository https://doi.org/10.17044/scilifelab.c.8014474). Based on the results, it was predicted that the large majority of bacterial species outside the anammox bacteria contained the reductive version of the enzyme and that the conversion of reductive to oxidative HAOs occurred in the ancestral node of the major clade in the anammox bacteria, followed by a reversion to a reductive form at the ancestral node of the subclade containing HAOr, HAO3 and HAO7. It was further predicted that HAO6 changed from a reductive to an oxidate enzyme in some species of “*Ca*. Brocadiaceae”.

### Horizontal Exchange of Genes for Subunits of the NXR Complex

The NXR in anammox bacteria can catalyze the oxidation of nitrite to nitrate and the reduction of nitrate to nitrite. We examined the evolution of the NXR subunits in the selected anammox bacterial species and their most closely related homologs in other bacterial species (Table S11). To this end, unrooted maximum likelihood phylogenies were inferred for each of the four subunits of the complex. In line with previous phylogenetic analysis of the NXR-A subunit (Lücker et al. 2010, 2013; Kitzinger et al. 2018), the anammox NXR-A clustered with proteins from *Nitrospirota* and the *Nitrospinota* (Fig. S9a). This clade belonged to an even larger clade that contained species from diverse phyla, such as *Proteobacteria* and *Halobacteriota* (archaea). Only two *Planctomycetota* species were identified in the tree, *Novopirellula aureliae* and *T. terrifontis*, but these species were not monophyletic with the anammox bacteria. The topology of the tree inferred from NXR-B resembled the tree topology obtained with NXR-A. Also, in this tree, *Nitrospirota* formed a sister clade to the anammox bacteria, but no members from *Nitrospinota* could be identified. Similar to the NXR-A phylogeny, *Halobacteriales* formed two paraphyletic clades with other species nested between them (Fig. S9b). Homologs to NXR-C were identified in species also containing NXR-A and NXR-B, but many nodes in the tree inferred from this subunit were weakly supported (Fig. S9c).

### The N-Terminal Sequences of the Anammox Enzymes Target a Reporter Protein into the Periplasmic Space of *Escherichia coli*

To test the hypothesis that the enzymes in the anammox pathway are first targeted into the periplasmic space, rather than directly into the anammoxosome, we examined whether the N-terminal sequences of anammox enzymes are able to target a reporter protein into the periplasm of *E. coli*. For this purpose, we used the alkaline phosphatase (PhoA) protein assay (Manoil 1991), which has been used previously to study protein localization in *E. coli* (Rapp et al. 2004; Daley et al. 2005). The PhoA protein has the unique property of giving a measurable fluorescent signal when reacting with *p*-nitrophenyl phosphate when the protein has its correct 3-dimensional folding, which happens only after transport into the periplasmic space (Manoil and Beckwith 1986).

We designed plasmids with synthetic gene constructs in which the N-terminal sequences of HDH and the γ-subunit of HZS from “*Ca.* Kuenenia stuttgartiensis” strain MBR1 were fused to the *phoA* gene lacking its own native signal sequence (Table S12). For comparison, the signal sequences of two periplasmic proteins in “*Ca.* Kuenenia stuttgartiensis” strain MBR1, a flagellar P-ring protein and an outer member lipoprotein-sorting protein, were also fused to the *phoA* gene (Table S12). Plasmids containing the synthetic gene constructs were transformed into *Escherichia coli* strain cc118, which has its chromosomally encoded *phoA* gene inactivated. As positive controls, we used plasmids containing the *phoA* gene with its native signal sequence as well as a fusion of the *lepB* and the *phoA* genes. As negative controls, we used a plasmid that contained the *phoA* gene without a signal sequence as well as *E. coli* cc118 cells without a plasmid. The results of the PhoA assays showed that plasmids containing the N-terminal sequences of the genes for HDH, the γ-subunit of HZS and the flagellar P-ring protein in “*Ca*. Kuenenia stuttgartiensis” yielded equally high or higher alkaline phosphatase activities as the positive controls, whereas no signals were detected for the negative controls (Table 1, Table S13). We conclude that the N-terminal sequences of HDH and the γ-subunit of HZS successfully targeted proteins into the periplasmic space of *E. coli*, which suggests that these enzymes are transported from the cytoplasm into the periplasm also in the cells of the anammox bacteria.

**Table 1:** Arbitrary activity units of the alkaline phosphatase (PhoA) assay

Sample type	Plasmid	Fusion constructs^a^	Activity units^b^
Anammoxosome	+	Hydrazine dehydrogenase (SP)	247
Anammoxosome	+	Hydrazine synthase (γ) (SP)	165
Periplasm	+	Flagellar P-ring (SP)	540
Periplasm	+	Lipoprotein-sorting protein (SP)	19
Positive control	+	Signal peptidase I (protein)	279
Positive control	+	Alkaline phosphatase (native)	69
Negative control	+	…	ND^c^
Negative control	–	…	ND^c^

^a^The signal peptide sequences (SP) or the full-length gene sequence of the enzyme (protein) were fused to the *phoA* gene. The native gene for alkaline phosphatase was used as a positive control.

^b^Activity units represent the average values of 3 biological samples with 3 replicates/sample.

^c^Estimated activity units below zero. ND = not detected.

## Discussion

The anammox pathway is restricted to a monophyletic group of bacteria in the *Planctomycetota*. While the biochemical characterization of this pathway is well studied, our understanding of its origin and evolution is patchy because of the lack of identifiable homologs to one of the key enzymes, HZS. We reasoned that a better understanding of the origin of the enzymes involved in the anammox pathway may give insights into how the anammoxosome has evolved to become the only energy-producing organelle known so far in bacteria.

For the study, we selected a set of genomes from the anammox bacteria so as to cover their known diversity, while ensuring a balanced dataset with regard to the number of taxa included per family. This set included high-quality genomes obtained from enrichment cultures of strains assigned to the families “*Ca*. Brocadia” and “*Ca*. Scalindua”, as well as MAGs obtained from environmental samples and assigned to either of these two families or to an earlier diverging family for which no enrichment cultures are available. The assembly of genomes from environmental samples is notoriously difficult, especially if the sample contains multiple closely related genomes. Not only may a gene not detected be a false negative, but genes located on different contigs in the same MAG may not necessarily be derived from the same strain, but could represent a mixture of genes from different strains. The MAGs that were selected for this study had been assembled into <200 contigs, whereas the MAGs that were discarded consisted of up to a thousand contigs. Thus, while we consider the selected set of genomes and MAGs to be of sufficient quality for the analyses, adding more MAGs would only risk introducing noise into the analyses in the form of sequencing errors, misassembled contigs and contaminating sequences from other bacteria.

Starting out from this genome dataset, we have revised and extended the evolutionary analysis of the key enzymes in the anammox reaction, focusing mostly on the highly expressed subunits of the HZS complex while also re-examining the origin of the other enzymes in the pathway. We first searched for putative homologs to the subunits of the HZS complex using both sequence-based and structure-based methods. Our survey indicated that very distantly related homologs to the α-subunit could be identified in a few obligate anaerobic bacteria, such as *A. giovannonii, T. terrifontis*, and *N. artificiosorum* which belong to the phylum *Planctomycetota*, and *S. fumaroxidans*, a sulfate-reducing bacterium from the phylum *Desulfobacterota*. Like the α-subunit of the HZS complex in the anammox bacteria, most of the putative homologs contained two heme-binding sites. However, key residues of importance for the enzymatic activity of the α-subunit of the HZS complex could not be identified. This suggests that the homologs are unable to catalyze the synthesis of hydrazine from NO and ammonium, although they are described in the PDB database as “HZS α-domain proteins”. Importantly for the purpose of this discussion, the results suggest that the anammox pathway for energy production is restricted to the anammox bacteria.

In contrast to the few homologs identified to the α-subunit of the HZS complex, our survey indicated that distantly related homologs to the fused βγ-subunit were highly prevalent in environmental bacteria, although only few hits were found when either the β or the γ subunit was used individually as the queries. Interestingly, we noted that the genes for putative homologs to the α-subunit tended to be located in the vicinity of genes showing weak sequence similarity to the fused βγ-subunits. The identified homologs in species other than the anammox bacteria contained two internal heme-binding sites plus an additional eight heme-binding sites at their N-terminal ends, suggesting that the ancestral enzymes may have been a fusion protein that contained up to ten heme-binding sites. However, given that the majority of modern anammox bacterial species contain two distinct genes for these two subunits, we hypothesize that a fission event occurred in the common ancestor of the anammox bacteria, but that the two genes fused again within the “*Ca.* Scalinduaceae” family.

We reasoned that studies of the expansion of the HAO protein family in the anammox bacteria may provide insights into when and how the transition between reductive and oxidative functions occurred. Oxidative HAOs share structural similarities and form trimers or multiples of trimers (Maalcke et al. 2014; Akram et al. 2019). The monomers are connected via a unique cross-link between the Tyrosine residue in one subunit and the heme 4 active site of another subunit, called the P_460_ prosthetic group (Caranto et al. 2016). Consistently, the Tyrosine residue was identified in the oxidative enzymes HDH and HOX in the anammox bacteria and in the HAOs of the AOBs. Conversely, it has been predicted that HAOs that lack this unique cross-link, such as HAOr and HAO3, perform reductive functions (Kartal and Keltjens 2016), as was also recently confirmed in catalytic and spectroscopic studies of HAOr (Ferousi et al. 2021).

To study when the transitions occurred, we inferred the sequences of the ancestral HAO enzymes and mapped the occurrence of the Tyrosine residue onto the nodes in the phylogenetic tree based on the HAO proteins, using the same nomenclature for the paralogous HAO families (HAO1 to HAO10) as in Okubo et al. 2021. The diversification pattern of these families in our phylogeny was largely consistent with the tree topology obtained previously in Okubo et al. 2021, although we included a broader diversity of outgroup genomes. The results of the ancestral reconstruction suggested that the conversion of a reductive enzyme with Histidine at the key site in the protein to an oxidative enzyme with Tyrosine at this site occurred at the ancestral node of the major anammox clade, which contains as many as nine HAO enzymes from the anammox bacteria. The analyses also indicated two more recent transitions within the HAO6 clade, but only in some species in “*Ca*. Brocadiaceae”. Finally, the analyses provided indications of a separate transition from a reductive (His) to an oxidative (Tyr) HAO enzyme within the phylum *Desulfobacterota*.

The phylogenies also indicated recent gene exchanges of HAOs between the anammox bacteria and members of the *Desulfobacterota* (Fig. S8). The single lineages HAO5A, HAO5B and HAO6 along with a few other bacterial and archaeal species were embedded within very large clades that contained HAO enzymes from many species assigned to the phylum *Desulfobacterota*, including for example *Desulfuromonas acetoxidans*, which is used in microbial fuel cells due to its ability to produce electricity and has as many as 47 putative, multiheme *c*-type cytochromes (Alves et al. 2011). Thus, there is a high abundance and huge diversity of multiheme cytochrome *c* proteins for electron transfer in environmental bacteria that share a similar anaerobic lifestyle but are otherwise unrelated to the anammox bacteria.

Based on the results of the ancestral sequence reconstruction, we suggest that the evolution of the HAOs in the anammox bacteria occurred by a three-step process. The transition from (i) a reductive to an oxidative HAOs was an early event that preceded (ii) a series of gene duplication events, after which (iii) the common ancestor of the anammox bacteria diversified into different lineages. This scenario is consistent with the idea that the last common ancestor of the modern anammox bacteria can be traced back to around the GOE when oxygen and nitrite levels increased in the atmosphere (Liao et al. 2022), while many of the enzymes emerged earlier, in anoxic environments. Furthermore, the identification of the homologs to the HZS complex strongly suggests that the potential for evolving the anammox pathway might have existed elsewhere, yet no other bacterial group have evolved energy-producing organelles. Thus, other aspects of the biology of those bacterial species might not have supported such a process. We believe that the origin of the anammoxosome was such a key biological event, without which the energy-producing anammox reaction could not have evolved.

The anammoxosome has a single membrane layer rich in ladderane phospholipids (Sinninghe Damsté et al. 2002, 2004, 2005; Boumann et al. 2006; Nouri and Tantillo 2006; Rattray et al. 2008), which represent “some of the most structurally exotic lipids known” (Moss et al. 2018). It has been hypothesized that the ladderane membranes may have prevented the diffusion of the toxic compound hydrazine (Kartal et al. 2013) or enabled protons (Jetten et al. 2009) or other reactive species to be trapped inside the organelle (Nouri and Tantillo 2012). However, experimental studies based on a hydrazine transmembrane diffusion assay showed that the ladderane membranes were permeable to hydrazine (Moss et al. 2018), arguing against the hypothesis that the organelle evolved to protect the cytoplasm and the periplasm from this toxic compound. Rather, it was observed that the proton/hydroxide equilibrium was 5 to 10 times slower compared to normal membranes, suggesting that the role of the ladderane lipids may rather be to prevent the breakdown of the proton motive force (Moss et al. 2018). Thus, the acquisition of enzymes in the anammox pathway combined with the presence of intracellular membranes with ladderane lipids may have provided the ancestral anammox bacterial cell with a competitive edge that could not be matched by any other bacterial cell.

An intriguing question to ask is whether the anammoxosome originated from inside the bacterial cell or via fusion with another bacterial cell. The anammox bacteria belong to the *Planctomycetota,* a bacterial phylum that is well known for its variety of highly complex intracellular network structures (Fuerst 2005; Lee et al. 2009; Fuerst and Sagulenko 2011; Pinos et al. 2016) and it has recently been shown that some species of the phylum are able to ingest other bacteria by phagocytosis (Shiratori et al. 2019; Wiegand et al. 2019). This raises the possibility that the anamoxosome might have originated by phagocytosis of another bacterial cell, in analogy to the bacterial origin of mitochondria and chloroplasts in the eukaryotic cell. However, the results presented here and elsewhere (Lücker et al. 2010, 2013; Kitzinger et al. 2018; Hu et al. 2019; Liao et al. 2022) suggest that the enzymes in the anammox pathway were not acquired from a single donor, but rather assembled from a variety of obligate, anaerobic bacteria. For example, the HAOs showed sequence similarity to proteins in members of the *Desulfobacterota*, the NXR subunits to proteins in *Nitrospina* and *Nitrospira*, and homologs to the subunits of the HZS complex are present in anaerobic species in the *Planctomycetota* and *Desulfobacterota.* Thus, the most plausible scenario is that the ancestral enzymes were acquired by horizontal gene transfer, whereas the organelle itself originated from invaginations of the inner membrane of the anammox bacterial cell. Favoring such a hypothesis is also that the ATP synthase complex is facing the cytoplasm, whereas the ATP is synthesized inside the mitochondria and chloroplasts, respectively.

In parallel with the evolution of the anammoxosome, systems to ensure that the enzymes were targeted into the anammoxosome must have evolved. In analogy to the mitochondrial targeting peptides, it has been hypothesized that enzymes involved in the anammox pathway contain unique anammoxosome targeting sequences, however no such signal sequences could be identified by predictive machine learning approaches (Medema et al. 2010). By fusing the N-terminal sequences of HDH and the γ-subunit of HZS to a marker gene (*phoA*) and expressing the construct in *E. coli*, we were able to shown that the N-terminal sequences of the anammox enzymes were able to target the PhoA protein into the periplasm. This strongly suggests that the anammox enzymes have evolved from periplasmic proteins, and that the modern enzymes are targeted to the periplasm prior to being transferred into the anammoxosome, a process that is possibly mediated by membrane vesicles.

Taken together, our study suggests that enzymes involved in the anammox reaction evolved from periplasmic, multiheme-binding enzymes in ancestral obligate anaerobic bacteria by sub-functionalization. These findings are inconsistent with the hypothesis that the key enzyme complex in the pathway, HZS, originated by de novo birth (Liao et al. 2022). However, we also found no evidence to suggest that the anammox reaction is more widespread than currently appreciated, as was recently suggested by Suarez et al. 2023. Rather, our study suggests that although other anaerobic bacteria may have had the potential to evolve enzymes capable of performing the anammox reaction, they may not have been able to host the process due to the lack of organelles with membranes containing ladderane lipids and thereby unable to make this process a source of energy. Coupling studies on the evolution of the anammox pathway, as in this study, with cell biological research on the emergence of intracellular organelles and transport systems will be needed to fully understand the origin and evolution of the enigmatic anammox bacteria.

## Methods

### Datasets

A dataset was constructed using all taxa under the class “*Ca.* Brocadiia” in the NCBI taxonomy as of November 2022 (*n* = 227), under the class *Brocadiae* in the GTDB taxonomy v.207 (Chaumeil et al. 2022; Parks et al. 2022) (*n* = 38), and additional taxa covering all major clades of the PVC superphylum (*n* = 58) (Odelgard et al. 2024). Genomes for the selected taxa were downloaded from GenBank. Prokka v.1.14.6 (Seemann 2014) was used to annotate taxa without annotation provided in GenBank and also for selected taxa missing key anammox enzymes in their annotation. The proteins for the members of the “*Ca.* Brocadiia” were clustered into protein families using OrthoFinder v.2.5.4 (Emms and Kelly 2019) with default settings.

### Identification of Homologs to Enzymes in the Anammox Pathway

A Diamond database was constructed for the proteins from the species in the extended dataset and the additional taxa from the PVC superphylum. Proteins from “*Candidatus* Kuenenia stuttgartiensis” that have previously been identified in the anammoxosome and the anammox pathway were used as queries against this database using Diamond v.2.0.15 (Buchfink et al. 2015) with the --ultra-sensitive argument and report hits with the *E*-value lower than 10^−6^.

A GTDB Diamond database was also built from the proteins of representative species in the GTDB v207. Proteomes were obtained from NCBI RefSeq/GenBank, and for genomes without submitted annotation in NCBI, the Prodigal derived proteomes provided by GTDB were used. Proteins involved in the anammox reaction were used as queries in searches against this database using Diamond v.2.0.15 with the following settings; --very-sensitive, --*E*-value < 10^−6^, --max-target-seqs 2000, --subject-cover 50, --query-coverage 50, --bitscore 80. Additional HZS-A homologs were identified by conducting an HMM search using hmmsearch from HMMER v.3.3.2 (Eddy 2011) with the HZS-α middle domain Pfam profile (PF18582) to the proteomes from RefSeq containing a hit to HZS-βγ.

The experimentally determined structure of the HZS complex in “*Ca.* Kuenenia stuttgartiensis” (PDB accession 5C2V) (Dietl et al. 2015) was downloaded from the Protein Data Bank (PDB), and the predicted structure of the βγ subunits of the HZS complex in “*Ca.* Scalindua japonica” from the AlphaFold Protein Structure Database (Jumper et al. 2021; Varadi et al. 2022, 2024). Both protein structures were used as queries in a protein structure searches using Foldseek (standard parameters) (van Kempen et al. 2024).

### Structure Alignments

The predicted structure of the proteins that yielded hits to the α and βγ subunits of HZS complex were downloaded from the AlphaFold database. The structures were aligned against the predicted structures of HZS-α and HZS-βγ from “*Ca.* Scalindua japonica” using the TMalign software. For the structural comparison to the homolog from *Syntrophobacter fumaroxidans,* the alignment was done in PyMOL 2.5.5 (The PyMOL Molecular Graphics System, Version 2.0 Schrödinger, LLC.) against the structure of αβγ subunits of HZS complex in “*Ca.* Kuenenia stuttgartiensis” (PDB accession 5C2 V).

### Phylogenetic Inferences and Ancestral Sequence Reconstruction

An HMM profile for bacterial RpoB (COG0085) was obtained from EggNOG v.5.0 (Huerta-Cepas et al. 2019) and used as a query with hmmsearch from HMMER v.3.3.2 (Eddy 2011) to identify RpoB in the proteomes (best hit, *E* < 10^−30^). The RpoB protein sequences from *Escherichia coli* K12 and *Bacillus subtilis* strain 168 were included to root the phylogeny. The RpoB sequences were aligned with MAFFT L-INS-i v.7.471 (Katoh and Standley 2013) and the alignment was trimmed with trimAl v.1.4.1 (Capella-Gutiérrez et al. 2009) using the -automated1 option. A phylogeny was computed with IQ-TREE v.2.2.0 (Minh et al. 2020) under the LG + G4 + F substitution model with 1,000 ultrafast bootstrap replicates (Hoang et al. 2018) and 1,000 SH-aLRT replicates (Guindon et al. 2010). Long-branching taxa and taxa classified as “*Ca.* Brocadiia” but located outside of the known diversity of anammox were excluded, and the phylogeny was recalculated using the same method.

The phylogenies for HZS, HAO, and NXR was calculated based on hits derived from RefSeq based on the search against GTDB. For the phylogeny of the HAO-like proteins, sequences from the ONR protein family were added as an outgroup (Soares et al. 2022). MSAs were calculated with MAFFT using the L-INS-I strategy. MSAs were trimmed with trimAl v1.4.1 using the -automated1 option for all phylogenies except for the phylogeny of the HAO-like proteins where the -gt 0.5 option was used. Initial phylogenies were calculated with FastTree v2.1.10 (Price et al. 2010), and subsequent manual removal of long-branching taxa was performed. Next, alignment and trimming were performed using the same approach on the cleaned dataset, and phylogenies were calculated with IQ-Tree v2.2.0 under the best-fitting model (Kalyaanamoorthy et al. 2017) selected from LG and WAG. Support values for the branches were calculated using 1,000 ultrafast bootstrap replicates and 1,000 SH-aLRT replicates. For NXR-A and NXR-B, phylogenies with fewer taxa were created by extracting the clade containing the anammox proteins and recalculating the alignment and the phylogenies for this clade. Phylogenies were visualized and plotted with FigTree (https://github.com/rambaut/figtree/). MSAs of HZS-α and HZS-βγ were visualized with ESPript 3 (Robert and Gouet 2014). The computed HAO alignment and phylogenetic tree were used in IQ-Tree v2.2.0 (--asr-min 0.95). The same substitution model, as selected during the computation of the phylogenetic tree (WAG + I + I + R10), was used for ancestral sequence reconstruction. The output was analyzed to extract the model's prediction of the presence or absence of the tyrosine cross-link at each node using an in-house script (see Data Availability) and visualized using iTOL (Letunic and Bork 2024).

### Genome Synteny Visualization

The genome synteny visualizations were created with pyGenomeViz (https://github.com/moshi4/pyGenomeViz).

### Plasmid Constructions

The pHA-1 plasmid (Sääf et al. 1999) was used as a template for the construction of the PhoA fusion plasmids. Synthetic gene constructs with the selected set of signal peptides fused to the 5´end of the *phoA* gene were ordered from Thermo Fisher. The constructs included a positive and a negative control, which contained the *phoA* gene sequence with and without its signal sequence. Three sets of primers were designed to amplify the synthetic gene constructs as well as two segments of the plasmid containing the origin of replication, the ampicillin resistance gene and the arabinose induction system. Gradient PCR was used for the amplifications and the PCR products were gel purified prior to assembly by Gibson assembly (Gibson et al. 2009) according to standard protocol (New England Biolabs, E5510S). In addition, an already existing plasmid with the *lepB* gene fused to the *phoA* gene (Daley et al. 2005) was used as a positive control. The plasmids were transformed into *E. coli* strain 10 beta (New England Biolabs, C3020K). Ampicillin resistant colonies were isolated and the plasmids were purified using PureYield mini prep kit (Promega, A1223) according to standard protocols. All plasmids were sequenced by Oxford nanopore technology at a minimum of 250×to confirm that the PCR products had been assembled correctly.

### Activity Measurements of PhoA Fusion Proteins

For the *phoA* assay, the pHA-1 plasmids with the different gene constructs were transformed into *E. coli* c118. Cells from an overnight culture of *E. coli* cc118 harboring the plasmid were re-inoculated and the expression of the gene constructs were induced with 8 µl of 20% arabinose. Cells were grown until mid-exponential phase where 1 ml of the cell cultures were harvested and 4 µl of 200 mM iodoacetamide (to inactivate the cytoplasmic activity of PhoA) (Derman and Beckwith 1995) in 10 mM Tris-HCL, pH 8.0 were added and the solutions were incubated for 5 min at 37 °C. The bacterial cells were centrifuged and the pellets were washed in 1 ml of wash-buffer (10 mM Tris-HCL, pH 8.0, 10 mM MgSO_4_, 1 mM iodoacetamide). After centrifugation, the pellets were resuspended in 0.8 ml of resuspension buffer (1 M Tris-HCL, pH 8.0, 1 mM iodoacetamide), from which 100 µl were extracted for OD_600_ measurements and 100 µl were added to 0.9 ml of activity buffer (1 M Tris-HCL, pH 8.0, 0.1 mM ZnCl_2_, 1 mM iodoacetamide). 4 µl of 0.1% SDS and 4 µl of chloroform were added and the solutions were incubated for 5 min at 37 °C with shaking to lyse the cells. The solutions were placed on ice for 5 min after which 100 µl 2% *p*-nitrophenyl phosphate substrate was added followed by 90 min incubation at 37 °C. Lastly, 100 µl of cells were taken to measure OD_405_ and OD_550_ in the plate reader. Arbitrary activity units were calculated as described below, where OD600 is the cell density at the start of the assay.


$$A=(OD_{405}-(1.75\times OD_{550}))1000/OD_{600}$$

The calculations of activity units were based on the formula presented in Manoil 1991 with the modification that the time of the assay (90 min) and the volume of the samples (0.1 ml), which were the same in all experiments, were not included.

## Supplementary Material

evaf244_Supplementary_Data

## Data Availability

Code and analysis pipelines supporting this work are available at https://github.com/emilhaegglund/anammxox_pathway_evo_publ and https://github.com/alejimgon/asr-nf. Sequence alignments, ancestral reconstruction states and plasmid sequences are provided in the SciLifeLab repository (https://doi.org/10.17044/scilifelab.c.8014474).

## References

[evaf244-B1] Akram M, et al A 192-heme electron transfer network in the hydrazine dehydrogenase complex. Sci Adv. 2019:5:eaav4310. 10.1126/sciadv.aav4310. 31001586 PMC6469936

[evaf244-B2] Ali M, et al Physiological characterization of anaerobic ammonium oxidizing bacterium ‘*Candidatus* Jettenia caeni’. Environ Microbiol. 2015:17:2172–2189. 10.1111/1462-2920.12674. 25367004

[evaf244-B3] Ali M, et al Draft genome sequence of the anaerobic ammonium-oxidizing bacterium “*Candidatus* Brocadia sp. 40”. Genome Announc. 2016:4:e01377-16. 10.1128/genomeA.01377-16. 27932661 PMC5146453

[evaf244-B4] Ali M, Shaw DR, Albertsen M, Saikaly PE. Comparative genome-centric analysis of freshwater and marine ANAMMOX cultures suggests functional redundancy in nitrogen removal processes. Front Microbiol. 2020:11:1–16. 10.3389/fmicb.2020.01637. 32733431 PMC7358590

[evaf244-B5] Alves AS, Paquete CM, Fonseca BM, Louro RO. Exploration of the ‘cytochromome’ of *Desulfuromonas acetoxidans*, a marine bacterium capable of powering microbial fuel cells. Metallomics. 2011:3:349. 10.1039/c0mt00084a. 21298162

[evaf244-B6] Boumann HA, et al Ladderane phospholipids in anammox bacteria comprise phosphocholine and phosphoethanolamine headgroups. FEMS Microbiol Lett. 2006:258:297–304. 10.1111/j.1574-6968.2006.00233.x. 16640588

[evaf244-B7] Broda E . Two kinds of lithotrophs missing in nature. Z Allg Mikrobiol. 1977:17:491–493. 10.1002/jobm.19770170611. 930125

[evaf244-B8] Buchfink B, Xie C, Huson DH. Fast and sensitive protein alignment using DIAMOND. Nat Methods. 2015:12:59–60. 10.1038/nmeth.3176. 25402007

[evaf244-B9] Capella-Gutiérrez S, Silla-Martínez JM, Gabaldón T. Trimal: a tool for automated alignment trimming in large-scale phylogenetic analyses. Bioinformatics. 2009:25:1972–1973. 10.1093/bioinformatics/btp348. 19505945 PMC2712344

[evaf244-B10] Caranto JD, Vilbert AC, Lancaster KM. *Nitrosomonas europaea* cytochrome P460 is a direct link between nitrification and nitrous oxide emission. Proc Natl Acad Sci U S A. 2016:113:14704–14709. 10.1073/pnas.1611051113. 27856762 PMC5187719

[evaf244-B11] Chaumeil PA, Mussig AJ, Hugenholtz P, Parks DH. GTDB-Tk v2: memory friendly classification with the genome taxonomy database. Bioinformatics. 2022:38:5315–5316. 10.1093/bioinformatics/btac672. 36218463 PMC9710552

[evaf244-B12] Chicano TM, et al Structural and functional characterization of the intracellular filament-forming nitrite oxidoreductase multiprotein complex. Nat Microbiol. 2021:6:1129–1139. 10.1038/s41564-021-00934-8. 34267357 PMC8387239

[evaf244-B13] Daley DO, et al Global topology analysis of the *Escherichia coli* inner membrane proteome. Science. 2005:308:1321–1323. 10.1126/science.1109730. 15919996

[evaf244-B14] de Almeida NM, et al Immunogold localization of key metabolic enzymes in the anammoxosome and on the tubule-like structures of Kuenenia stuttgartiensis. J Bacteriol. 2015:197:2432–2441. 10.1128/JB.00186-15. 25962914 PMC4524196

[evaf244-B15] Derman AI, Beckwith J. *Escherichia coli* alkaline phosphatase localized to the cytoplasm slowly acquires enzymatic activity in cells whose growth has been suspended: a caution for gene fusion studies. J Bacteriol. 1995:177:3764–3770. 10.1128/jb.177.13.3764-3770.1995. 7601842 PMC177094

[evaf244-B16] Dietl A, et al The inner workings of the hydrazine synthase multiprotein complex. Nature. 2015:527:394–397. 10.1038/nature15517. 26479033

[evaf244-B17] Ding C, Adrian L. Comparative genomics in “*Candidatus* Kuenenia stuttgartiensis” reveal high genomic plasticity in the overall genome structure, CRISPR loci and surface proteins. BMC Genomics. 2020:21:851. 10.1186/s12864-020-07242-1. 33261555 PMC7709395

[evaf244-B18] Eddy SR . Accelerated profile HMM searches. PLoS Comput Biol. 2011:7:e1002195. 10.1371/journal.pcbi.1002195. 22039361 PMC3197634

[evaf244-B19] Emms DM, Kelly S. OrthoFinder: phylogenetic orthology inference for comparative genomics. Genome Biol. 2019:20:238. 10.1186/s13059-019-1832-y. 31727128 PMC6857279

[evaf244-B20] Ferousi C, et al Characterization of a nitrite-reducing octaheme hydroxylamine oxidoreductase that lacks the tyrosine cross-link. J Biol Chem. 2021:296:100476. 10.1016/j.jbc.2021.100476. 33652023 PMC8042395

[evaf244-B21] Frank J, et al Resolving the complete genome of *Kuenenia stuttgartiensis* from a membrane bioreactor enrichment using Single-Molecule Real-Time sequencing. Sci Rep. 2018:8:4580. 10.1038/s41598-018-23053-7. 29545612 PMC5854607

[evaf244-B22] Fuerst JA . Intracellular compartmentation in Planctomycetes. Annu Rev Microbiol. 2005:59:299–328. 10.1146/annurev.micro.59.030804.121258. 15910279

[evaf244-B23] Fuerst JA, Sagulenko E. Beyond the bacterium: planctomycetes challenge our concepts of microbial structure and function. Nat Rev Microbiol. 2011:9:403–413. 10.1038/nrmicro2578. 21572457

[evaf244-B24] Gibson DG, et al Enzymatic assembly of DNA molecules up to several hundred kilobases. Nat Methods. 2009:6:343–345. 10.1038/NMETH.1318. 19363495

[evaf244-B25] Guindon S, et al New algorithms and methods to estimate maximum-likelihood phylogenies: assessing the performance of PhyML 3.0. Syst Biol. 2010:59:307–321. 10.1093/sysbio/syq010. 20525638

[evaf244-B26] Hira D, et al Anammox organism KSU-1 expresses a NirK-type copper-containing nitrite reductase instead of a NirS-type with cytochrome *cd*_1_. FEBS Lett. 2012:586:1658–1663. 10.1016/j.febslet.2012.04.041. 22673575

[evaf244-B27] Hoang DT, Chernomor O, von Haeseler A, Minh BQ, Vinh LS. UFBoot2: improving the ultrafast bootstrap approximation. Mol Biol Evol. 2018:35:518–522. 10.1093/molbev/msx281. 29077904 PMC5850222

[evaf244-B28] Hu Z, Wessels HJCT, van Alen T, Jetten MSM, Kartal B. Nitric oxide-dependent anaerobic ammonium oxidation. Nat Commun. 2019:10:1244. 10.1038/s41467-019-09268-w. 30886150 PMC6423088

[evaf244-B29] Huerta-Cepas J, et al eggNOG 5.0: a hierarchical, functionally and phylogenetically annotated orthology resource based on 5090 organisms and 2502 viruses. Nucleic Acids Res. 2019:47:D309–D314. 10.1093/nar/gky1085. 30418610 PMC6324079

[evaf244-B30] Jetten MSM, et al Biochemistry and molecular biology of anammox bacteria. Crit Rev Biochem Mol Biol. 2009:44:65–84. 10.1080/10409230902722783. 19247843

[evaf244-B31] Jumper J, et al Highly accurate protein structure prediction with AlphaFold. Nature. 2021:596:583–589. 10.1038/s41586-021-03819-2. 34265844 PMC8371605

[evaf244-B32] Kalyaanamoorthy S, Minh BQ, Wong TKF, von Haeseler A, Jermiin LS. ModelFinder: fast model selection for accurate phylogenetic estimates. Nat Methods. 2017:14:587–589. 10.1038/nmeth.4285. 28481363 PMC5453245

[evaf244-B33] Kartal B, et al How to make a living from anaerobic ammonium oxidation. FEMS Microbiol Rev. 2013:37:428–461. 10.1111/1574-6976.12014. 23210799

[evaf244-B34] Kartal B, Keltjens JT. Anammox biochemistry: a tale of heme c proteins. Trends Biochem Sci. 2016:41:998–1011. 10.1016/j.tibs.2016.08.015. 27669648

[evaf244-B35] Katoh K, Standley DM. MAFFT multiple sequence alignment software version 7: improvements in performance and usability. Mol Biol Evol. 2013:30:772–780. 10.1093/molbev/mst010. 23329690 PMC3603318

[evaf244-B36] Kitzinger K, et al Characterization of the first “Candidatus nitrotoga” isolate reveals metabolic versatility and separate evolution of widespread nitrite-oxidizing bacteria. mBio. 2018:9:e01186-18. 10.1128/mBio.01186-18. 29991589 PMC6050957

[evaf244-B37] Kuenen JG . Anammox bacteria: from discovery to application. Nat Rev Microbiol. 2008:6:320–326. 10.1038/nrmicro1857. 18340342

[evaf244-B38] Lam P, Kuypers MMM. Microbial nitrogen cycling processes in oxygen minimum zones. Ann Rev Mar Sci. 2011:3:317–345. 10.1146/annurev-marine-120709-142814. 21329208

[evaf244-B39] Lee K-C, Webb RI, Fuerst JA. The cell cycle of the planctomycete *Gemmata obscuriglobus* with respect to cell compartmentalization. BMC Cell Biol. 2009:10:4. 10.1186/1471-2121-10-4. 19144151 PMC2656463

[evaf244-B40] Letunic I, Bork P. Interactive Tree of Life (iTOL) v6: recent updates to the phylogenetic tree display and annotation tool. Nucleic Acids Res. 2024:52:W78–W82. 10.1093/nar/gkae268. 38613393 PMC11223838

[evaf244-B41] Liao T, Wang S, Stüeken EE, Luo H. Phylogenomic evidence for the origin of obligate anaerobic anammox bacteria around the great oxidation event Ursula Battistuzzi. Mol Biol Evol. 2022:39:msac170. 10.1093/molbev/msac170. 35920138 PMC9387917

[evaf244-B42] Lücker S, et al A *Nitrospira* metagenome illuminates the physiology and evolution of globally important nitrite-oxidizing bacteria. Proc Natl Acad Sci U S A. 2010:107:13479–13484. 10.1073/pnas.1003860107. 20624973 PMC2922143

[evaf244-B43] Lücker S, Nowka B, Rattei T, Spieck E, Daims H. The genome of *Nitrospina gracilis* illuminates the metabolism and evolution of the major marine nitrite oxidizer. Front Microbiol. 2013:4:27. 10.3389/fmicb.2013.00027. 23439773 PMC3578206

[evaf244-B44] Maalcke WJ, et al Structural basis of biological NO generation by octaheme oxidoreductases. J Biol Chem. 2014:289:1228–1242. 10.1074/jbc.M113.525147. 24302732 PMC3894310

[evaf244-B45] Maalcke WJ, et al Characterization of anammox hydrazine dehydrogenase, a key N_2_-producing enzyme in the global nitrogen cycle. J Biol Chem. 2016:291:17077–17092. 10.1074/jbc.M116.735530. 27317665 PMC5016112

[evaf244-B46] Manoil C . Analysis of membrane protein topology using alkaline phosphatase and β-galactosidase gene fusions. Methods Cell Biol. 1991:34:61–75. 10.1016/S0091-679X(08)61676-3. 1943817

[evaf244-B47] Manoil C, Beckwith J. A genetic approach to analyzing membrane protein topology. Science. 1986:233:1403–1408. 10.1126/science.3529391. 3529391

[evaf244-B48] Medema MH, et al A predicted physicochemically distinct sub-proteome associated with the intracellular organelle of the anammox bacterium *Kuenenia stuttgartiensis*. BMC Genomics. 2010:11:299. 10.1186/1471-2164-11-299. 20459862 PMC2881027

[evaf244-B49] Minh BQ, et al IQ-TREE 2: new models and efficient methods for phylogenetic inference in the genomic era. Mol Biol Evol. 2020:37:1530–1534. 10.1093/molbev/msaa015. 32011700 PMC7182206

[evaf244-B50] Moss FR, et al Ladderane phospholipids form a densely packed membrane with normal hydrazine and anomalously low proton/hydroxide permeability. Proc Natl Acad Sci U S A. 2018:115:9098–9103. 10.1073/pnas.1810706115. 30150407 PMC6140541

[evaf244-B51] Mulder A . Anaerobic ammonium oxidation discovered in a denitrifying fluidized bed reactor. FEMS Microbiol Ecol. 1995:16:177–183. 10.1016/0168-6496(94)00081-7.

[evaf244-B52] Narita Y, et al Enrichment and physiological characterization of an anaerobic ammonium-oxidizing bacterium ‘*Candidatus* Brocadia sapporoensis’. Syst Appl Microbiol. 2017:40:448–457. 10.1016/j.syapm.2017.07.004. 28869058

[evaf244-B53] Neumann S, et al Isolation and characterization of a prokaryotic cell organelle from the anammox bacterium *Kuenenia stuttgartiensis*. Mol Microbiol. 2014:94:794–802. 10.1111/mmi.12816. 25287816

[evaf244-B54] Nouri DH, Tantillo DJ. They came from the deep: syntheses, applications, and biology of ladderanes. Curr Org Chem. 2006:10:2055–2074. 10.2174/138527206778742678.

[evaf244-B55] Nouri DH, Tantillo DJ. Attack of radicals and protons on ladderane lipids: quantum chemical calculations and biological implications. Org Biomol Chem. 2012:10:5514. 10.1039/c2ob25717c. 22699391

[evaf244-B56] Odelgard A, Hägglund E, Guy L, Andersson SGE. Phylogeny and expansion of serine/threonine kinases in phagocytotic bacteria in the phylum planctomycetota. Genome Biol Evol. 2024:16:evae068. 10.1093/GBE/EVAE068. 38547507 PMC11032199

[evaf244-B57] Okubo T, et al The physiological potential of anammox bacteria as revealed by their core genome structure. DNA Res. 2021:28:dsaa028. 10.1093/dnares/dsaa028. 33367889 PMC7814187

[evaf244-B58] Oren A . Anammox revisited: thermodynamic considerations in early studies of the microbial nitrogen cycle. FEMS Microbiol Lett. 2015:362:fnv114. 10.1093/femsle/fnv114. 26174999

[evaf244-B59] Oren A . Candidatus List No. 4: lists of names of prokaryotic Candidatus taxa. Int J Syst Evol Microbiol. 2022:72:005545. 10.1099/ijsem.0.005545. 35100104

[evaf244-B60] Oshiki M, et al Genetic diversity of marine anaerobic ammonium-oxidizing bacteria as revealed by genomic and proteomic analyses of ‘*Candidatus* Scalindua japonica’. Environ Microbiol Rep. 2017:9:550–561. 10.1111/1758-2229.12586. 28892310

[evaf244-B61] Oshiki M, et al Metagenomic analysis of five phylogenetically distant anammox bacterial enrichment cultures. Microbes Environ. 2022:37:ME22017. 10.1264/jsme2.ME22017. 35811137 PMC9530715

[evaf244-B62] Oshiki M, Satoh H, Okabe S. Ecology and physiology of anaerobic ammonium oxidizing bacteria. Environ Microbiol. 2016:18:2784–2796. 10.1111/1462-2920.13134. 26616750

[evaf244-B63] Oshiki M, Shinyako-Hata K, Satoh H, Okabe S. Draft genome sequence of an anaerobic ammonium-oxidizing bacterium, “*Candidatus* Brocadia sinica”. Genome Announc. 2015:3:e00267-15. 10.1128/genomeA.00267-15. 25883286 PMC4400429

[evaf244-B64] Park H, Brotto AC, van Loosdrecht MCM, Chandran K. Discovery and metagenomic analysis of an anammox bacterial enrichment related to *Candidatus* “Brocadia caroliniensis” in a full-scale glycerol-fed nitritation-denitritation separate centrate treatment process. Water Res. 2017:111:265–273. 10.1016/j.watres.2017.01.011. 28088723

[evaf244-B65] Parks DH, et al GTDB: an ongoing census of bacterial and archaeal diversity through a phylogenetically consistent, rank normalized and complete genome-based taxonomy. Nucleic Acids Res. 2022:50:D785–D794. 10.1093/nar/gkab776. 34520557 PMC8728215

[evaf244-B66] Pinos S, Pontarotti P, Raoult D, Baudoin JP, Pagnier I. Compartmentalization in PVC super-phylum: evolution and impact. Biol Direct. 2016:11:38. 10.1186/s13062-016-0144-3. 27507008 PMC4977879

[evaf244-B67] Price MN, Dehal PS, Arkin AP. FastTree 2—approximately maximum-likelihood trees for large alignments. PLoS One. 2010:5:e9490. 10.1371/journal.pone.0009490. 20224823 PMC2835736

[evaf244-B68] Rapp M, et al Experimentally based topology models for *E. coli* inner membrane proteins. Protein Sci. 2004:13:937–945. 10.1110/ps.03553804. 15044727 PMC2280059

[evaf244-B69] Rattray JE, et al Ladderane lipid distribution in four genera of anammox bacteria. Arch Microbiol. 2008:190:51–66. 10.1007/s00203-008-0364-8. 18385981

[evaf244-B70] Robert X, Gouet P. Deciphering key features in protein structures with the new ENDscript server. Nucleic Acids Res. 2014:42:W320–W324. 10.1093/nar/gku316. 24753421 PMC4086106

[evaf244-B71] Sääf A, Johansson M, Wallin E, von Heijne G. Divergent evolution of membrane protein topology: the *Escherichia coli* RnfA and RnfE homologues. Proc Natl Acad Sci U S A. 1999:96:8540–8544. 10.1073/pnas.96.15.8540. 10411911 PMC17552

[evaf244-B72] Seemann T . Prokka: rapid prokaryotic genome annotation. Bioinformatics. 2014:30:2068–2069. 10.1093/bioinformatics/btu153. 24642063

[evaf244-B73] Shiratori T, Suzuki S, Kakizawa Y, Ishida K. Phagocytosis-like cell engulfment by a planctomycete bacterium. Nat Commun. 2019:10:5529. 10.1038/s41467-019-13499-2. 31827088 PMC6906331

[evaf244-B74] Sinninghe Damsté JS, et al Linearly concatenated cyclobutane lipids form a dense bacterial membrane. Nature. 2002:419:708–712. 10.1038/nature01128. 12384695

[evaf244-B75] Sinninghe Damsté JS, et al A mixed ladderane/n-alkyl glycerol diether membrane lipid in an anaerobic ammonium-oxidizing bacterium. Chem Commun. 2004:22:2590–2591. 10.1039/B409806D. 15543294

[evaf244-B76] Sinninghe Damsté JS, Rijpstra WIC, Geenevasen JAJ, Strous M, Jetten MSM. Structural identification of ladderane and other membrane lipids of planctomycetes capable of anaerobic ammonium oxidation (anammox). FEBS J. 2005:272:4270–4283. 10.1111/j.1742-4658.2005.04842.x. 16098207

[evaf244-B77] Soares R, Costa NL, Paquete CM, Andreini C, Louro RO. A new paradigm of multiheme cytochrome evolution by grafting and pruning protein modules. Mol Biol Evol. 2022:39:msac139. 10.1093/molbev/msac139. 35714268 PMC9250108

[evaf244-B78] Sonthiphand P, Hall MW, Neufeld JD. Biogeography of anaerobic ammonia-oxidizing (anammox) bacteria. Front Microbiol. 2014:5:399. 10.3389/fmicb.2014.00399. 25147546 PMC4123730

[evaf244-B79] Speth DR, et al Comparative genomics of two independently enriched “*Candidatus* kuenenia stuttgartiensis” anammox bacteria. Front Microbiol. 2012:3:307. 10.3389/fmicb.2012.00307. 22934093 PMC3423927

[evaf244-B80] Speth DR, et al Draft genome sequence of anammox bacterium “*Candidatus* Scalindua brodae,” obtained using differential coverage binning of sequencing data from two reactor enrichments. Genome Announc. 2015:3:e01415-14. 10.1128/genomeA.01415-14. 25573945 PMC4290996

[evaf244-B81] Speth DR, et al Draft genome of *Scalindua rubra*, obtained from the interface above the discovery deep brine in the Red Sea, sheds light on potential salt adaptation strategies in anammox bacteria. Microb Ecol. 2017:74:1–5. 10.1007/s00248-017-0929-7. 28074246 PMC5486813

[evaf244-B82] Speth DR, in ‘t Zandt MH, Guerrero-Cruz S, Dutilh BE, Jetten MSM. Genome-based microbial ecology of anammox granules in a full-scale wastewater treatment system. Nat Commun. 2016:7:11172. 10.1038/ncomms11172. 27029554 PMC4821891

[evaf244-B83] Stein LY, Klotz MG. The nitrogen cycle. Curr Biol. 2016:26:R94–R98. 10.1016/j.cub.2015.12.021. 26859274

[evaf244-B84] Strous M, et al Deciphering the evolution and metabolism of an anammox bacterium from a community genome. Nature. 2006:440:790–794. 10.1038/nature04647. 16598256

[evaf244-B85] Suarez C, et al Metagenomic evidence of a novel family of anammox bacteria in a subsea environment. Environ Microbiol. 2022:24:2348–2360. 10.1111/1462-2920.16006. 35415863 PMC9325076

[evaf244-B86] Suarez C, et al Novel and unusual genes for nitrogen and metal cycling in *Planctomycetota*—and KSB1-affiliated metagenome-assembled genomes reconstructed from a marine subsea tunnel. FEMS Microbiol Lett. 2023:370:fnad049. 10.1093/femsle/fnad049. 37291701 PMC10732223

[evaf244-B87] van de Graaf AA, et al Anaerobic oxidation of ammonium is a biologically mediated process. Appl Environ Microbiol. 1995:61:1246–1251. 10.1128/aem.61.4.1246-1251.1995. 7747947 PMC167380

[evaf244-B88] Van de Graaf AA, Mulder A, Slijkhuis H, Robertson LA, Kuenen JG. Anoxic ammonium oxidation. Proceedings of the 5th European Congress on Biotechnology. 15th ed. Vol 1; 1990; Copenhagen, Munksgaard International Publisher. p. 381–391.

[evaf244-B89] van Kempen M, et al Fast and accurate protein structure search with Foldseek. Nat Biotechnol. 2024:42:243–246. 10.1038/s41587-023-01773-0. 37156916 PMC10869269

[evaf244-B91] van Niftrik LA, et al The anammoxosome: an intracytoplasmic compartment in anammox bacteria. FEMS Microbiol Lett. 2004:233:7–13. 10.1016/j.femsle.2004.01.044. 15098544

[evaf244-B90] Van Niftrik L, et al Cell division ring, a new cell division protein and vertical inheritance of a bacterial organelle in anammox planrtomycetes. Mol Microbiol. 2009:73:1009–1019. 10.1111/j.1365-2958.2009.06841.x. 19708922

[evaf244-B92] Varadi M, et al AlphaFold Protein Structure Database: massively expanding the structural coverage of protein-sequence space with high-accuracy models. Nucleic Acids Res. 2022:50:D439–D444. 10.1093/nar/gkab1061. 34791371 PMC8728224

[evaf244-B93] Varadi M, et al AlphaFold Protein Structure Database in 2024: providing structure coverage for over 214 million protein sequences. Nucleic Acids Res. 2024:52:D368–D375. 10.1093/nar/gkad1011. 37933859 PMC10767828

[evaf244-B94] Wiegand S, et al The novel shapeshifting bacterial phylum Saltatorellota. bioRxiv 817700. 2019. 10.1101/817700.

[evaf244-B95] Zhao R, et al Geochemical transition zone powering microbial growth in subsurface sediments. Proc Natl Acad Sci U S A. 2020:117:32617–32626. 10.1073/pnas.2005917117. 33288718 PMC7768721

[evaf244-B96] Zhao R, Biddle JF, Jørgensen SL. Introducing *Candidatus* Bathyanammoxibiaceae, a family of bacteria with the anammox potential present in both marine and terrestrial environments. ISME Com. 2022:2:42. 10.1038/s43705-022-00125-4. PMC9723696 37938673

