# Supplementary material for: Origin and Evolution of Key Enzymes in the Anammox Pathway Revisited
Source: Genome Biol Evol. 2025 Dec 18;18(1):evaf244. doi: 10.1093/gbe/evaf244 (PMC12758564; doi:10.1093/gbe/evaf244)

Origin and evolution of key enzymes in the anammox pathway revisited

Emil Hägglund[1], Alejandro Jiménez-González[1], Lionel Guy[2] and Siv G. E. Andersson[1]

SUPPLEMENTARY LEGENDS

Supplementary Figures S1-S9

Supplementary Tables S1-S11

SUPPLEMENTARY TABLE REFERENCES

Supplementary Table S1 References

SUPPLEMENTARY FIGURES

Supplementary Figures S1-S9

Supplementary Figures

Figure S1. Maximum likelihood phylogeny of RNA polymerase subunit beta encoded by genes in (**A**) genomes classified as "Candidatus Brocadiia" and "Candidatus Brocadiae" according to the NCBI taxonomy and the GTDB taxonomy v207, respectively, together with 58 additional taxa from the PVC superphylum, (**B**) the same genomes as in (**A**) from which erroneously assigned taxa has been removed, (**C**) the high-quality and (**D**) the extended genome datasets. Taxa marked in orange and red in the phylogeny presented in (B) correspond to the taxa in the high-quality and extended genome datasets, respectively. Phylogenies were calculated with IQ-Tree v2.2.0 under the LG+G4+F substitution model. SH-aLRT and Ultrafast bootstrap support values are displayed on the branches. Sequence alignments are provided in the SciLifeLab repository (https://doi.org/10.17044/scilifelab.c.8014474).

Figure S2. Phyletic distribution pattern of key enzymes in the anammox pathway encoded by genes in the extended genome dataset and additional species from *Planctomycetota*, *Verrucumicrobiota*, and *Chlamydiota*. Species are coloured according to taxonomic affiliations. Colours in boxes show the percentage identity of the alignment according to the local BLAST searches. HZS-A, HZS-B and HZS-C = Subunits of hydrazine synthase complex; HAOr, HOX = Enzymes in the hydroxylamine oxidoreductase family; HDH = Hydrazine dehydrogenase; NXR-A, NXR-B, NXR-C, NXR-T = Subunits of the nitrite oxidoreductase complex; ATPA, ATPB, ATPD, ATPG = Subunits of the F-ATP synthase complex.

Figure S3. Overview of the hits obtained using the α-, β- and γ-subunits from "*Ca.* Kuenenia stuttgartiensis" and the α- and βγ-subunits from "*Ca.* Scalinduae" as queries in searches using Diamond Blastp against (**A**) representative species in GTDB v207 and (**B**) a subset of species from RefSeq. Distribution of the number of hits with a given percent identity, bit-score, and alignment length are shown.

Figure S4. Maximum likelihood phylogenies of the hydrazine synthase (**A**) α-subunit and (**B**) βγ-subunit encoded by genes in the high-quality genome dataset of the anammox bacteria and their putative homologs in other species. The gene order structure for the putative homologs to the (**C**) α- and (**D**) βγ-subunits of hydrazine synthase is shown. SH-aLRT and Ultrafast bootstrap support values are displayed on the branches. Sequence alignments are provided in the SciLifeLab repository (https://doi.org/10.17044/scilifelab.c.8014474).

Figure S5. Maximum likelihood phylogenies of the hydrazine synthase (**A**) α-subunit and (**B**) βγ-subunit encoded by genes in a few selected anammox bacteria and their putative homologs encoded by co-localized genes in other species. Pairwise amino acid sequence identities of the hydrazine synthase (**C**) α- and (**D**) βγ-subunits and their putative homologs encoded by co-localized genes in other species. SH-aLRT and Ultrafast bootstrap support values are displayed on the branches. Sequence alignments are provided in the SciLifeLab repository (https://doi.org/10.17044/scilifelab.c.8014474).

Figure S6. Comparison of structural similarity of the putative homologs to the hydrazine synthase α-subunit in "*Ca*. Kuenenia stuttgartiensis" (orange) and the βγ-subunits in *Ca*. Scalinduae (blue) to (**A**) representative species in GTDB v207 and (**B**) a subset of these species in RefSeq. The TM-scores are shown for each pairwise comparison. The TM-scores for the comparison of the hydrazine subunits in the anammox species to the putative homolog in *S. fumaroxidans* are marked with circles.

Figure S7. Comparison of the sequence similarity of the putative homologs to the hydrazine synthase subunits in the anammox bacteria. (**A**) Multiple sequence alignment of HZS-α and homologs in clade I, II, and III. **(B)** Multiple sequence alignment of HZS-βγ and homologs in clade I, II, and III.

Figure S8. Maximum likelihood phylogenies of octaheme proteins, including the hydroxylamine oxidoreductase-like proteins from the anammox bacteria encoded by genes in (**A**) the high-quality and (**B**) the extended datasets. SH-aLRT and Ultrafast bootstrap support values are displayed on the branches. (**C**) Ancestral sequence reconstruction of the tyrosine cross-link in the octaheme proteins. Sequence alignments are provided in the SciLifeLab repository (https://doi.org/10.17044/scilifelab.c.8014474).

Figure S9. Maximum likelihood phylogenies of nitrite oxidoreductase subunits, including proteins from the anammox bacteria encoded by genes in the extended genome dataset. The phylogenies are shown for (**A**) NXR-A, (**B**) NXR-B, (**C**) NXR-C, (**D**) NXR-D. SH-aLRT and Ultrafast bootstrap support values are displayed on the branches. The trees are unrooted. Sequence alignments are provided in the SciLifeLab repository (https://doi.org/10.17044/scilifelab.c.8014474).

Supplementary Tables

Table S1. Anammox genomes and MAGs included in this study.

Table S2. Taxa and proteins included in the phyletic distribution pattern shown in Figure S2, including sequence identity values to the query sequences.

Table S3. Summary of hits obtained in the BLAST search against RefSeq using the HZS subunits from anammox bacteria as the queries, summarized in Figure S3.

Table S4. Taxa, gene IDs, length, and annotation of proteins included in (**A**) HZS-α phylogeny shown in Figure S4A and (**B**) in HZS-βγ phylogeny shown in Figure S4B, including manually fused subunits of β and γ.

Table S5. Taxa, gene IDs, length, and annotation of proteins included in (**A**) HZS-α phylogeny shown in Figure S5A and (**B**) in HZS-βγ phylogeny shown in Figure S5B.

Table S6. Summary of hits obtained in the FoldSeek search using (**A**) the single α, β and y subunits of HZS from "*Ca*. Kuenenia stuttgartiensis" (5C2V) and (**B**) the fused by subunits of HZS from "*Ca*. Scalindua japonica".

Table S7. TM scores for the α and βγ subunits of hydrazine synthase to putative homologs in other bacteria shown in Figure S6.

Table S8. Taxa, gene IDs, gene lengths, and annotations of proteins included in the HAO phylogenies shown in Figure S7A.

Table S9. Taxa, gene IDs, gene lengths, and annotations of proteins included in the HAO phylogenies shown in Figure S7B.

Table S10. Data underlying the ancestral sequence reconstruction are provided in the SciLifeLab repository (https://doi.org/10.17044/scilifelab.c.8014474).

Table S11. Taxa, gene IDs, gene lengths, and annotations of proteins in the NXR phylogenies shown in Figure S8A-D.

Table S12. Gene IDs, Protein IDs, and sequences of the signal sequences used for the synthetic gene constructs.

Table S13. OD-values and arbitrary activity units in the phoA assay for each sample in the PhoA assay.

Table S1 - References

Ali M, Shaw DR, Albertsen M, Saikaly PE. 2020. Comparative Genome-Centric Analysis of Freshwater and Marine ANAMMOX Cultures Suggests Functional Redundancy in Nitrogen Removal Processes. *Front. Microbiol.* 11:1637.

Anantharaman K, Brown CT, Hug LA, Sharon I, Castelle CJ, Probst AJ, Thomas BC, Singh A, Wilkins MJ, Karaoz U, et al. 2016. Thousands of microbial genomes shed light on interconnected biogeochemical processes in an aquifer system. *Nat. Commun.* 7:13219.

Frank J, Lücker S, Vossen RHAM, Jetten MSM, Hall RJ, Op den Camp HJM, Anvar SY. 2018. Resolving the complete genome of Kuenenia stuttgartiensis from a membrane bioreactor enrichment using Single-Molecule Real-Time sequencing. *Sci. Rep.* 8:4580.

He C, Keren R, Whittaker ML, Farag IF, Doudna JA, Cate JHD, Banfield JF. 2021. Genome-resolved metagenomics reveals site-specific diversity of episymbiotic CPR bacteria and DPANN archaea in groundwater ecosystems. *Nat Microbiol* 6:354–365.

Kallistova A, Nikolaev Y, Grachev V, Beletsky A, Gruzdev E, Kadnikov V, Dorofeev A, Berestovskaya J, Pelevina A, Zekker I, et al. 2022. New Insight Into the Interspecies Shift of Anammox Bacteria Ca. “Brocadia” and Ca. “Jettenia” in Reactors Fed With Formate and Folate. *Front. Microbiol.* [Internet] 12. Available from: https://www.frontiersin.org/articles/10.3389/fmicb.2021.802201

Lin H, Ascher DB, Myung Y, Lamborg CH, Hallam SJ, Gionfriddo CM, Holt KE, Moreau JW. 2021. Mercury methylation by metabolically versatile and cosmopolitan marine bacteria. *ISME J.* 15:1810–1825.

Michoud G, Ngugi DK, Barozzi A, Merlino G, Calleja ML, Delgado-Huertas A, Morán XAG, Daffonchio D. 2021. Fine-scale metabolic discontinuity in a stratified prokaryote microbiome of a Red Sea deep halocline. *ISME J.* 15:2351–2365.

Okubo T, Toyoda A, Fukuhara K, Uchiyama I, Harigaya Y, Kuroiwa M, Suzuki T, Murakami Y, Suwa Y, Takami H. 2021. The physiological potential of anammox bacteria as revealed by their core genome structure. *DNA Res.* [Internet] 28. Available from: http://dx.doi.org/10.1093/dnares/dsaa028

Oshiki M, Mizuto K, Kimura Z-I, Kindaichi T, Satoh H, Okabe S. 2017. Genetic diversity of marine anaerobic ammonium-oxidizing bacteria as revealed by genomic and proteomic analyses of “Candidatus Scalindua japonica.” *Environ. Microbiol. Rep.* 9:550–561.

Oshiki M, Shinyako-Hata K, Satoh H, Okabe S. 2015. Draft Genome Sequence of an Anaerobic Ammonium-Oxidizing Bacterium, “Candidatus Brocadia sinica.” *Genome Announc.* [Internet] 3. Available from: http://dx.doi.org/10.1128/genomeA.00267-15

Suarez C, Dalcin Martins P, Jetten MSM, Karačić S, Wilén BM, Modin O, Hagelia P, Hermansson M, Persson F. 2022. Metagenomic evidence of a novel family of anammox bacteria in a subsea environment. *Environ. Microbiol.* 24:2348–2360.

Wiegand S, Jogler M, Boedeker C, Pinto D, Vollmers J, Rivas-Marín E, Kohn T, Peeters SH, Heuer A, Rast P, et al. 2020. Cultivation and functional characterization of 79 planctomycetes uncovers their unique biology. *Nat Microbiol* 5:126–140.

Yang Y, Lu Z, Azari M, Kartal B, Du H, Cai M, Herbold CW, Ding X, Denecke M, Li X, et al. 2022. Discovery of a new genus of anaerobic ammonium oxidizing bacteria with a mechanism for oxygen tolerance. *Water Res.* 226:119165.

Zhao R, Biddle JF, Jørgensen SL. 2022. Introducing Candidatus Bathyanammoxibiaceae, a family of bacteria with the anammox potential present in both marine and terrestrial environments. *ISME Communications* 2:1–9.

Zhao R, Mogollón JM, Abby SS, Schleper C, Biddle JF, Roerdink DL, Thorseth IH, Jørgensen SL. 2020. Geochemical transition zone powering microbial growth in subsurface sediments. *Proc. Natl. Acad. Sci. U. S. A.* 117:32617–32626.

Supplementary Figure S1A

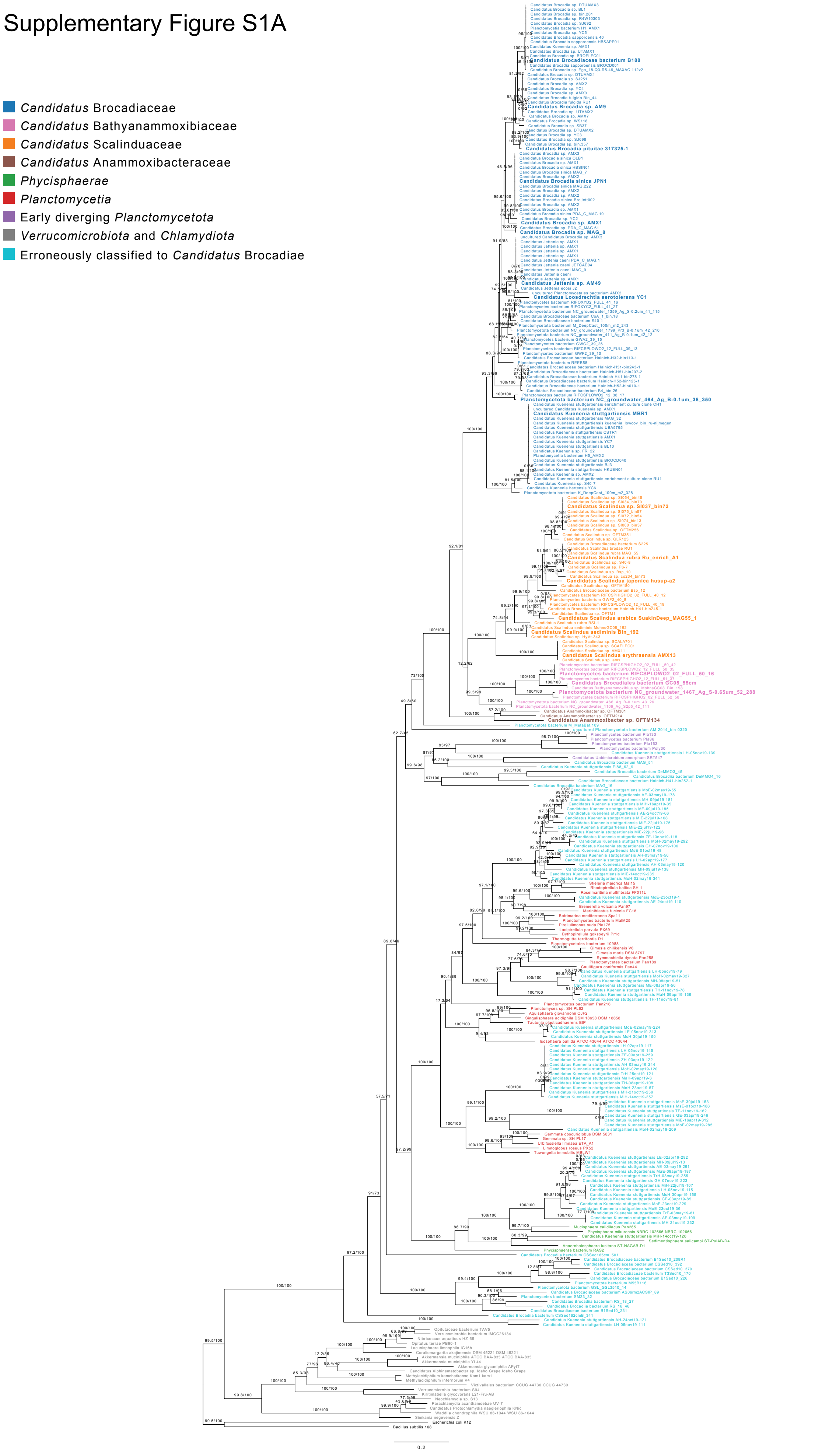

Supplementary Figure S1B

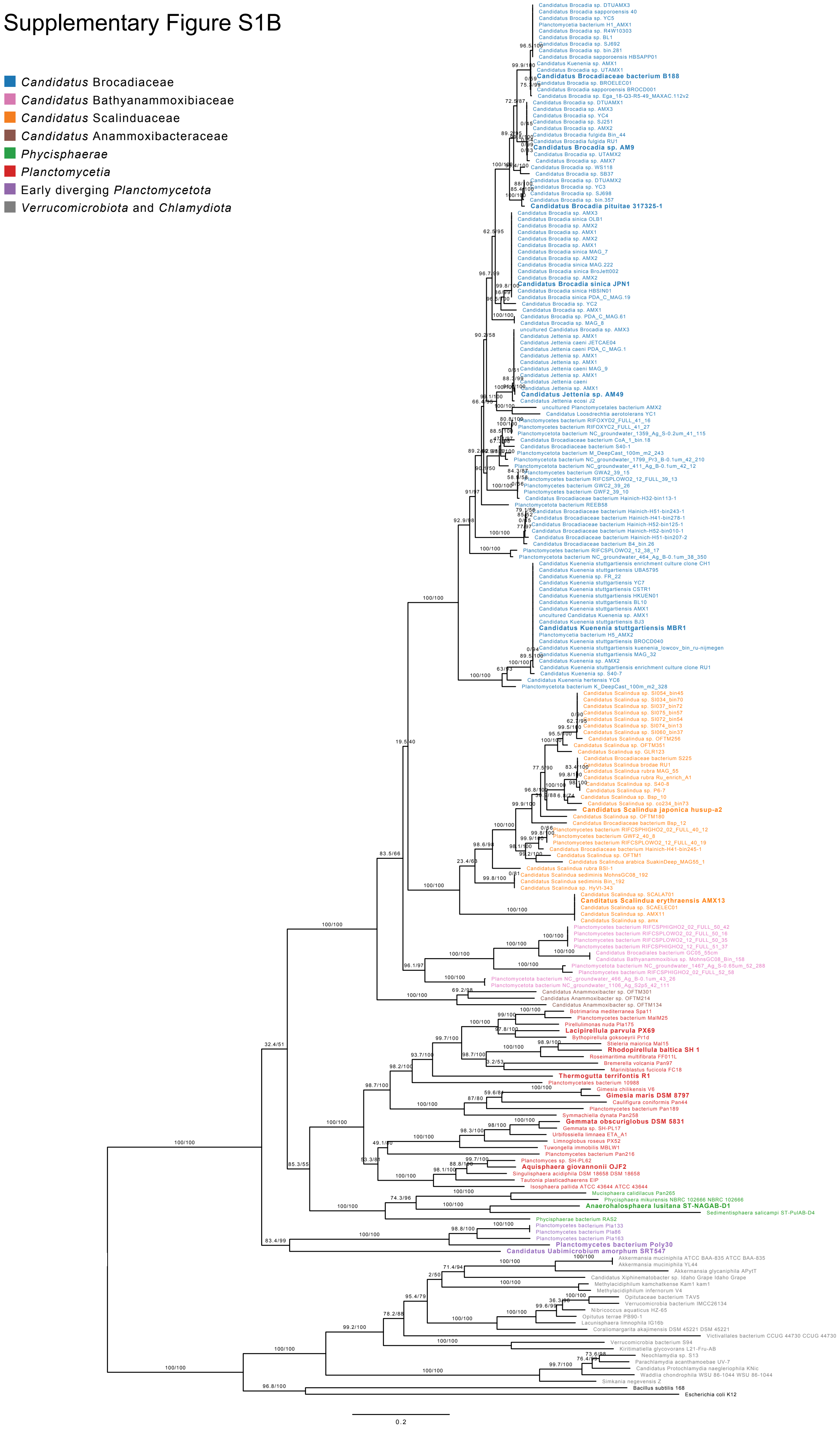

Supplementary Figure S1C

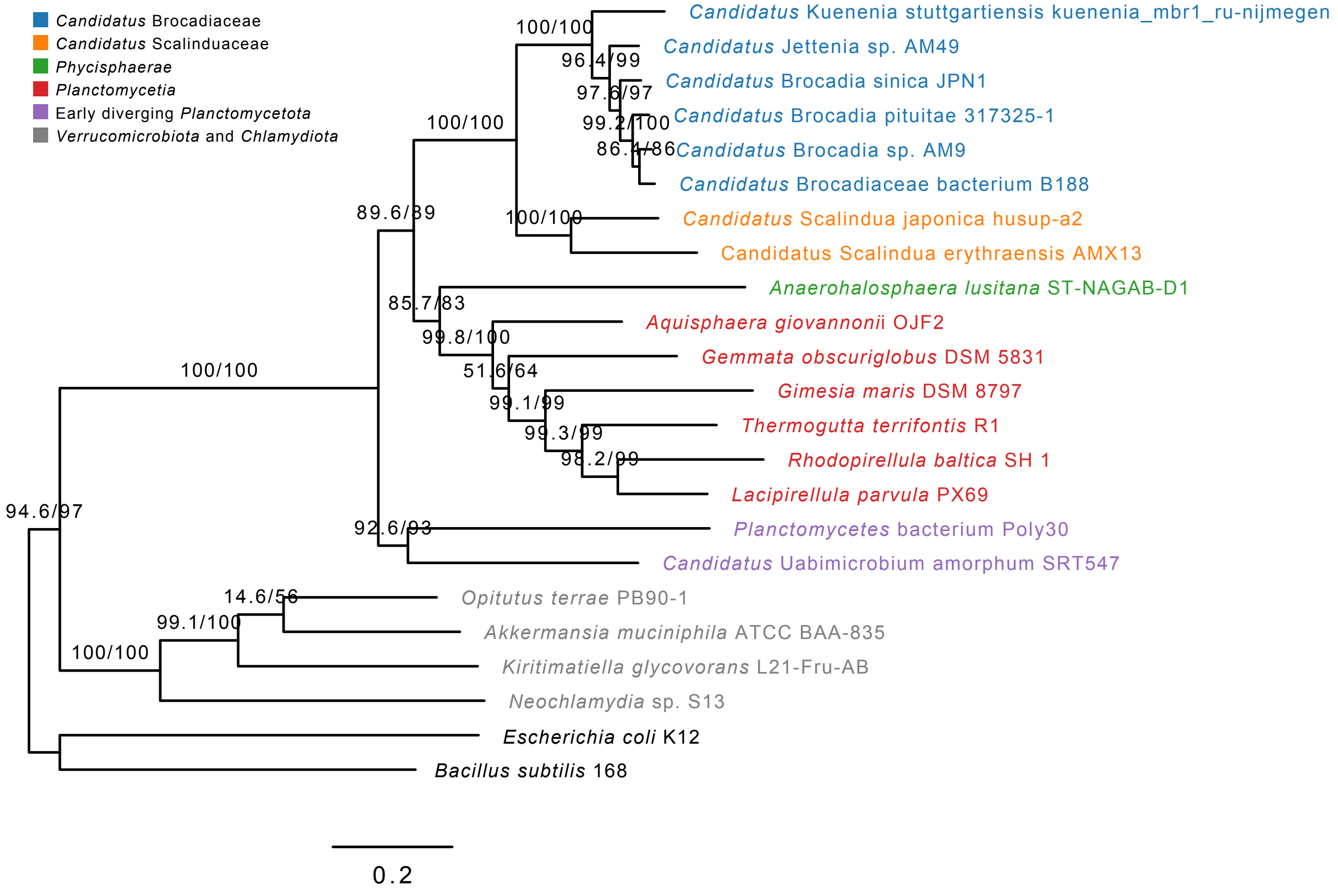

Supplementary Figure S1D

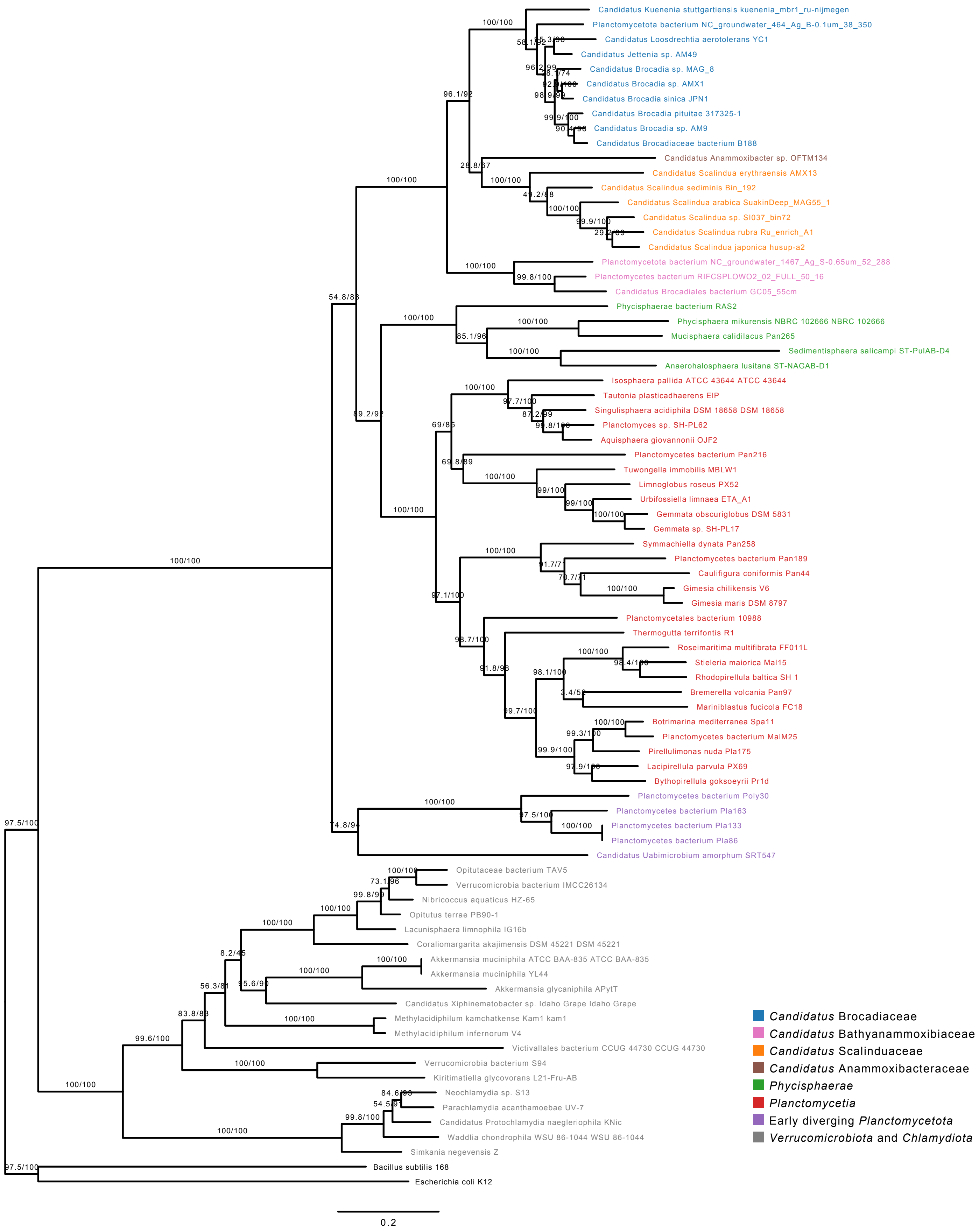

Supplementary Figure S2

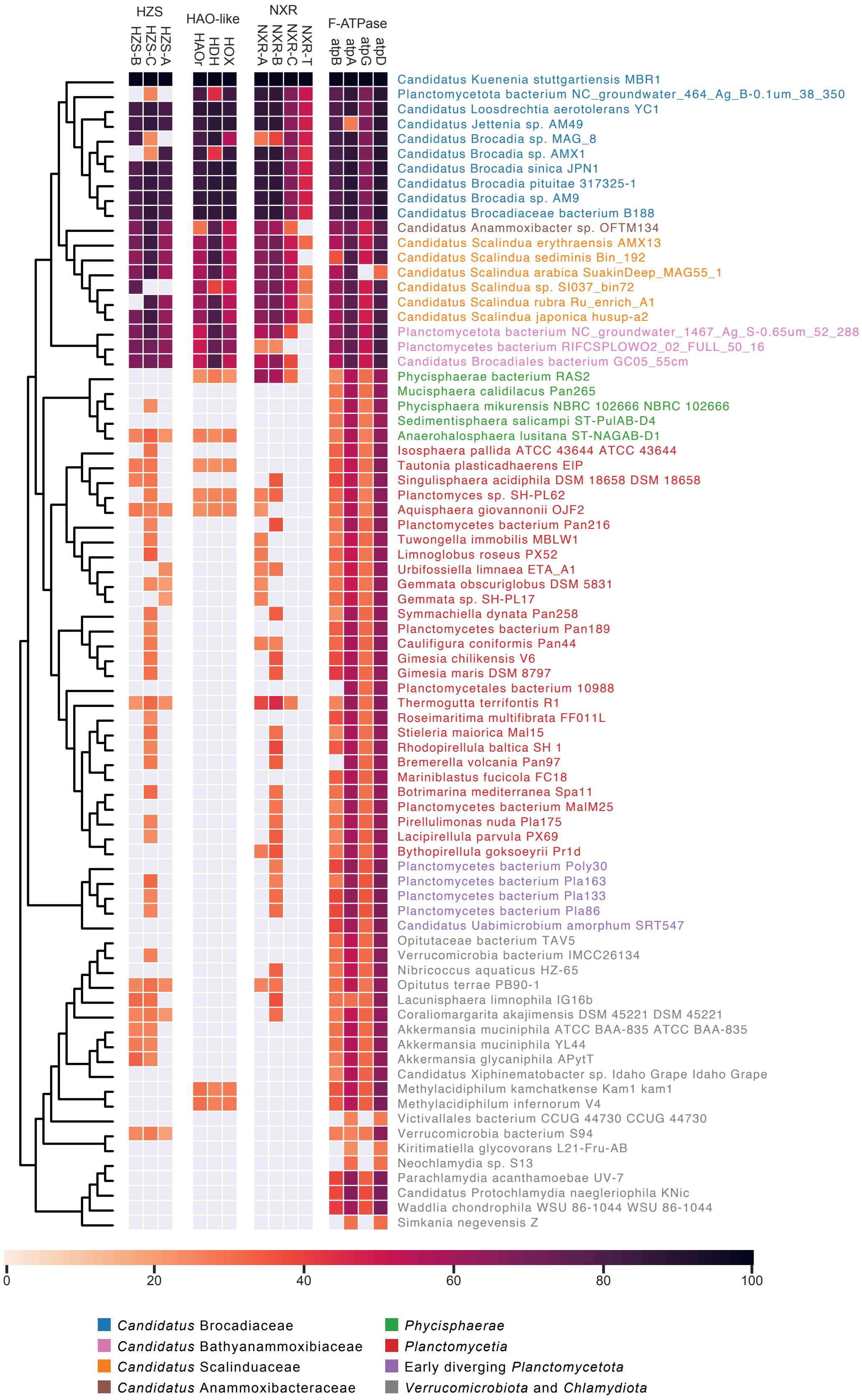

Supplementary Figure S3

A

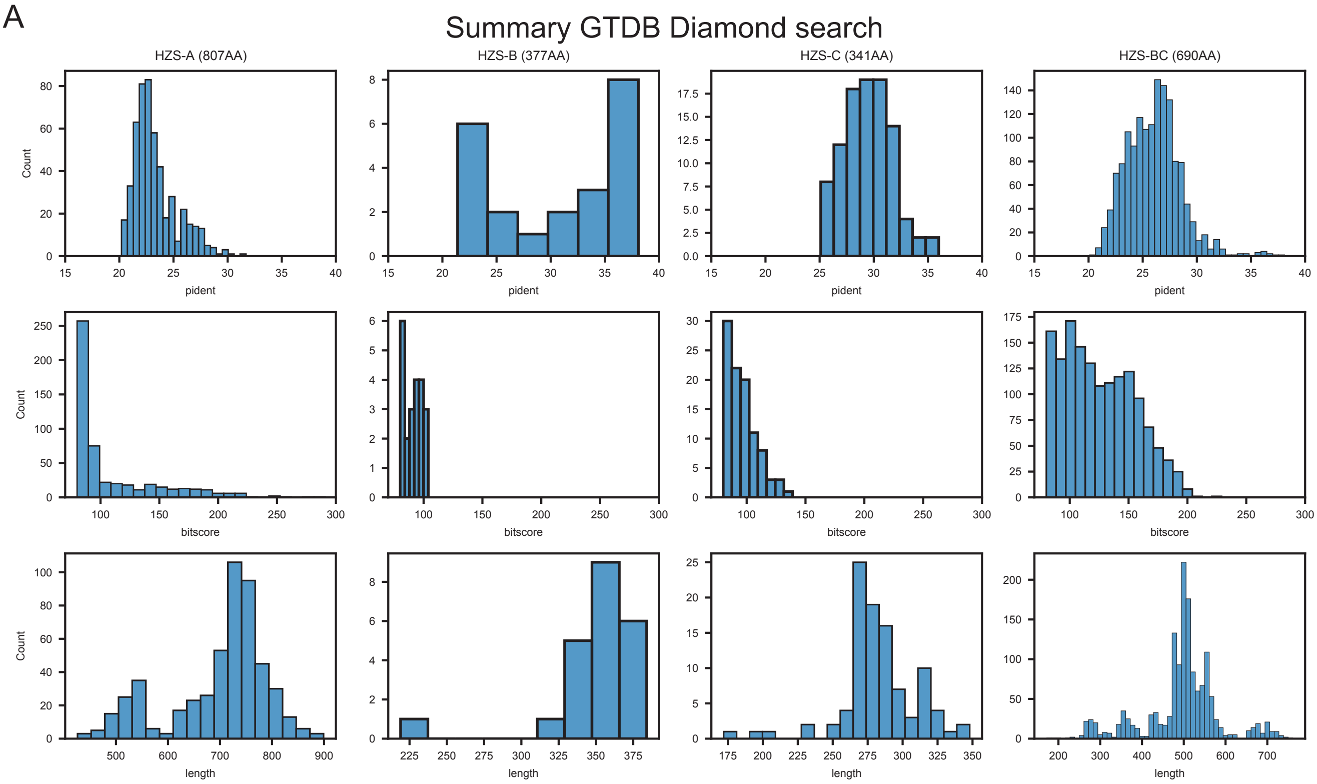

B

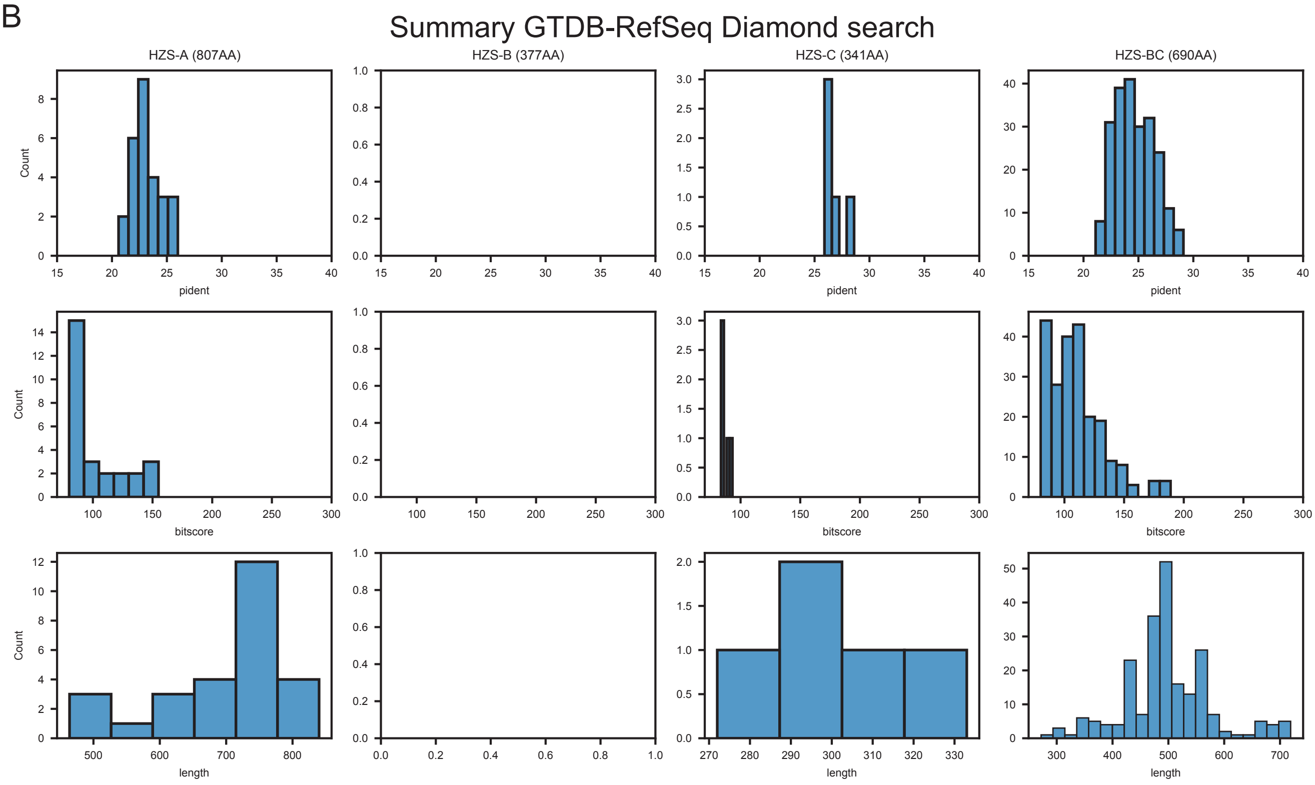

Supplementary Figure S4A

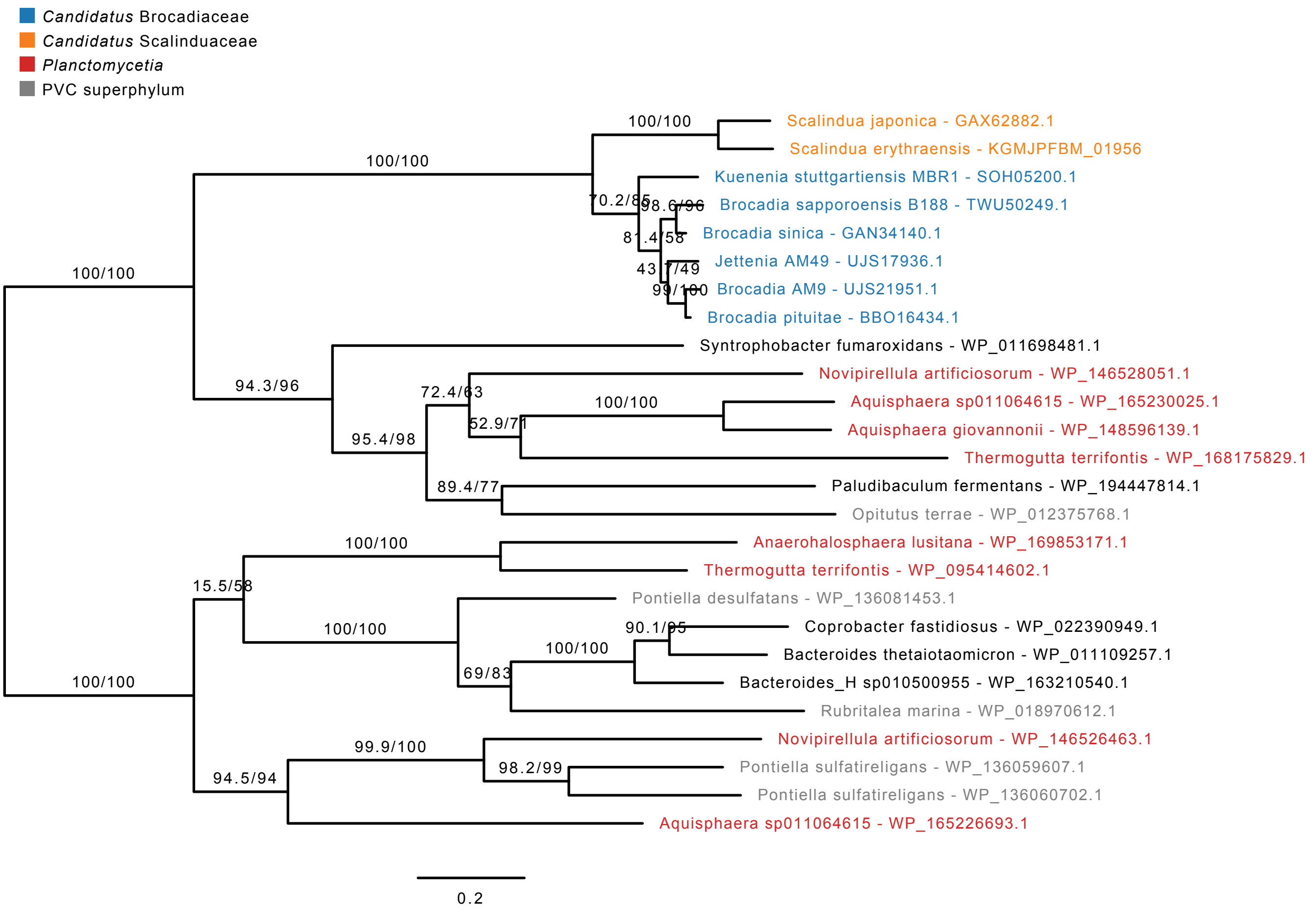

Supplementary Figure S4B

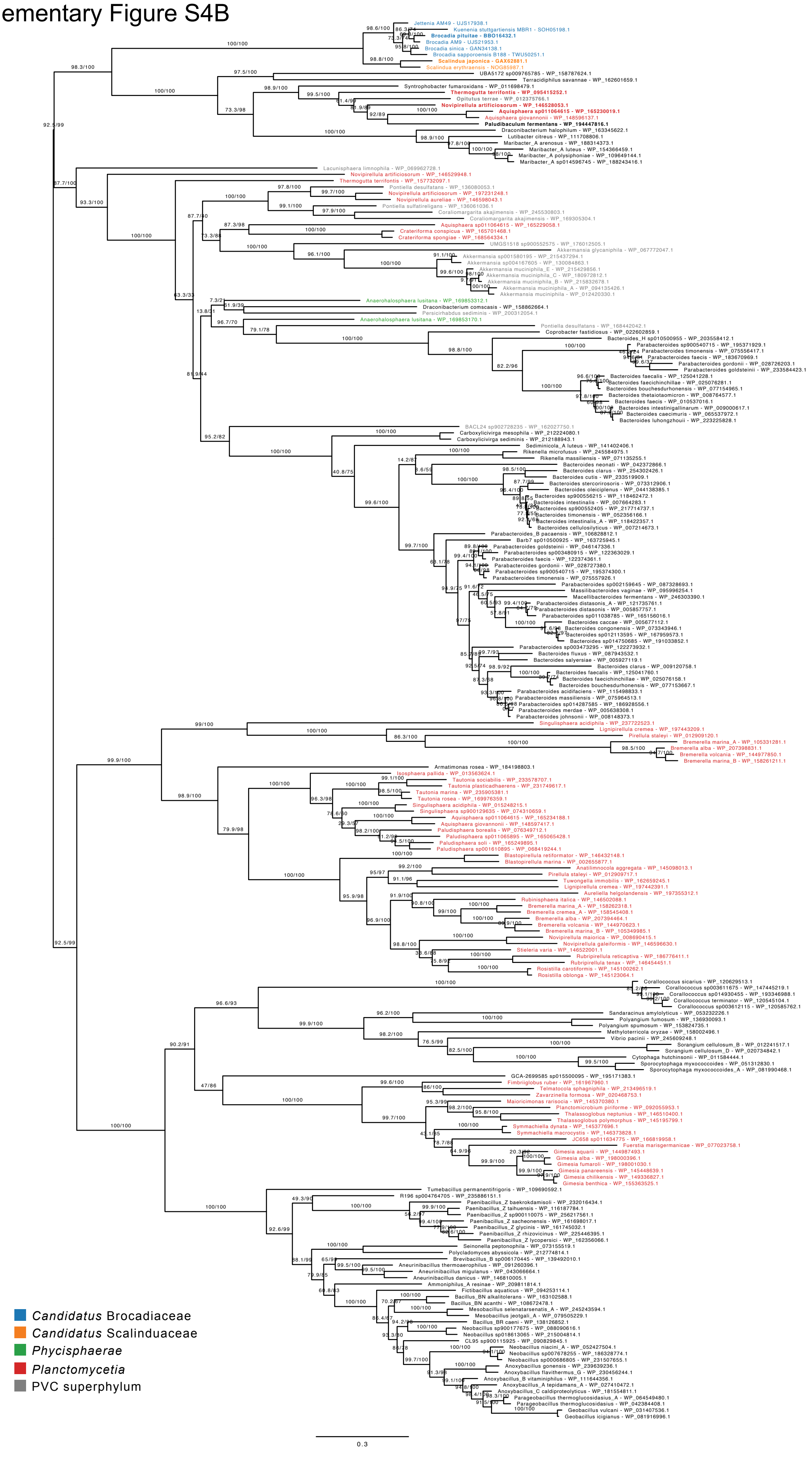

Supplementary Figure S4C

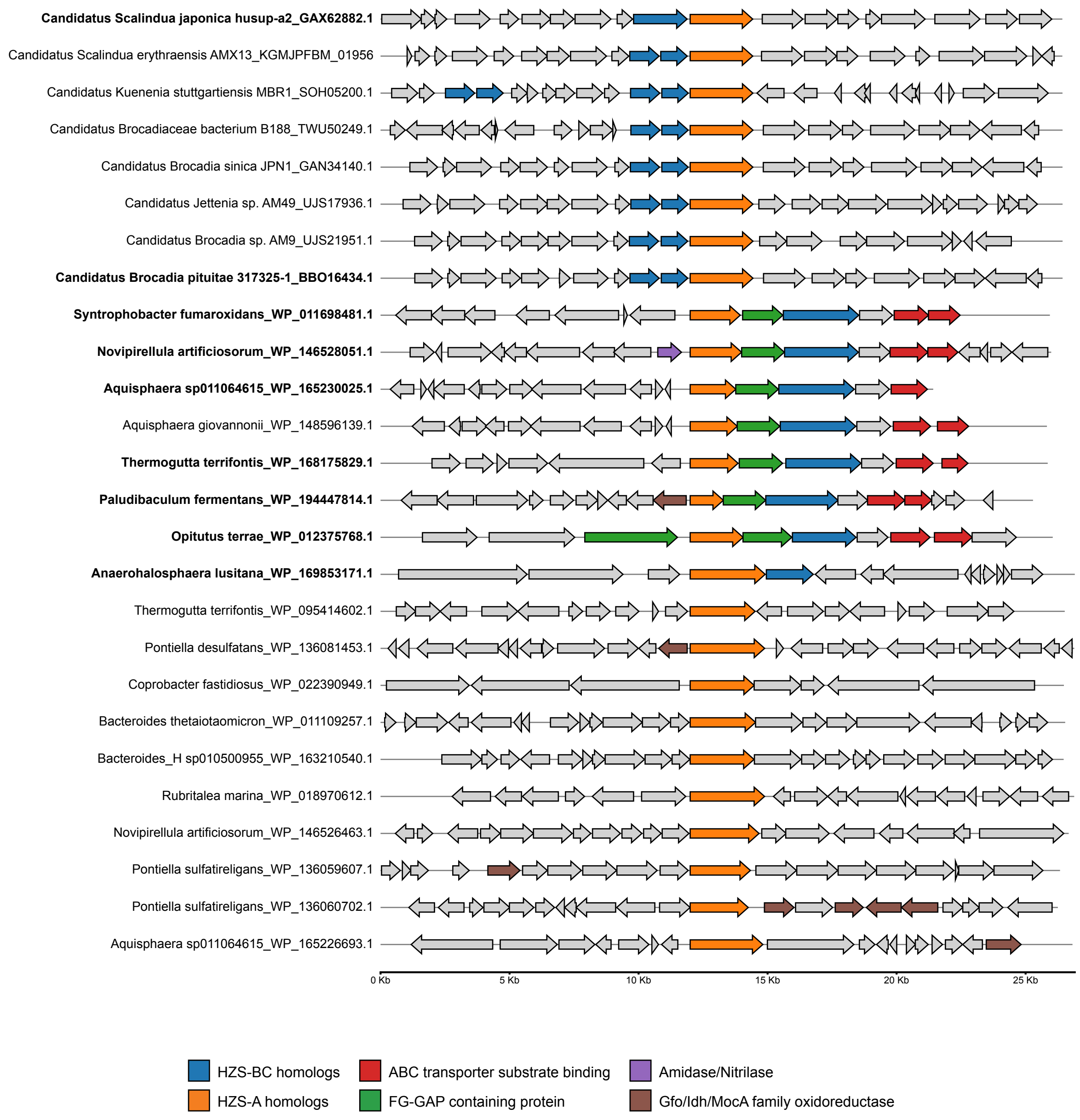

Supplementary Figure S4D

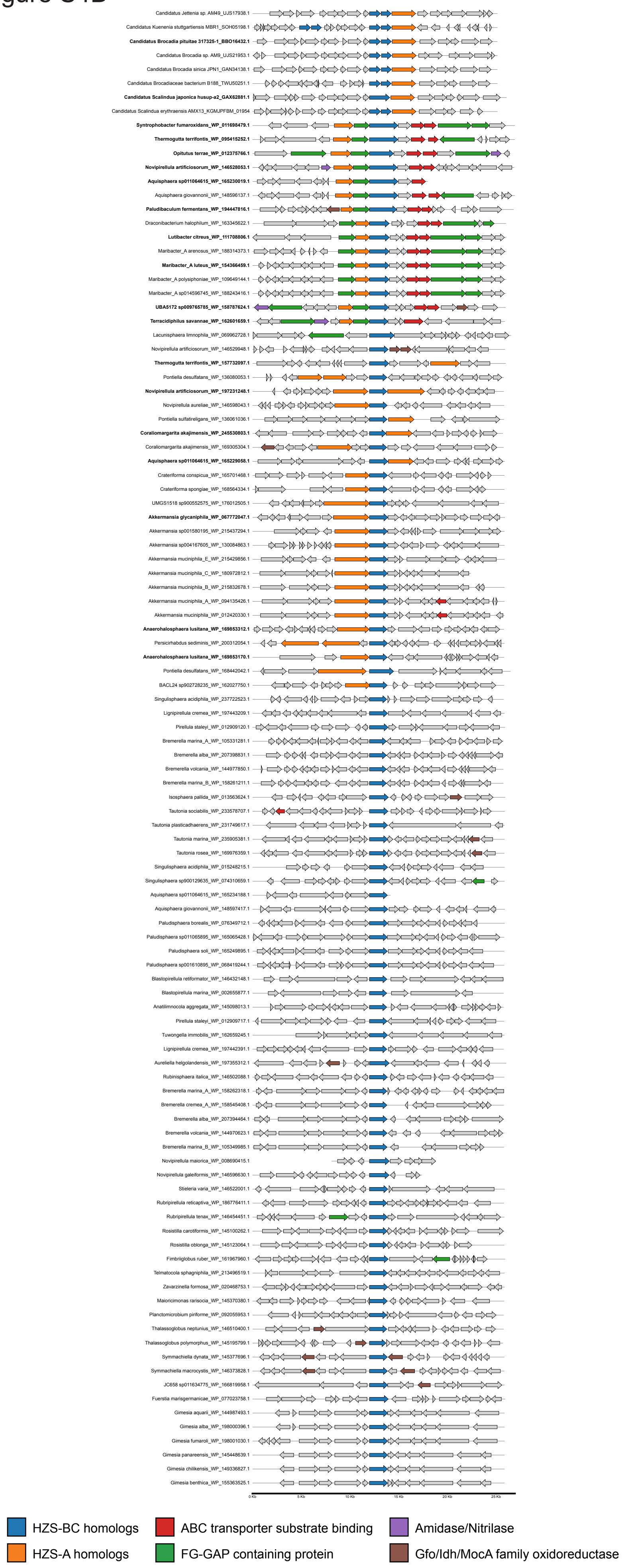

Supplementary Figure S5A

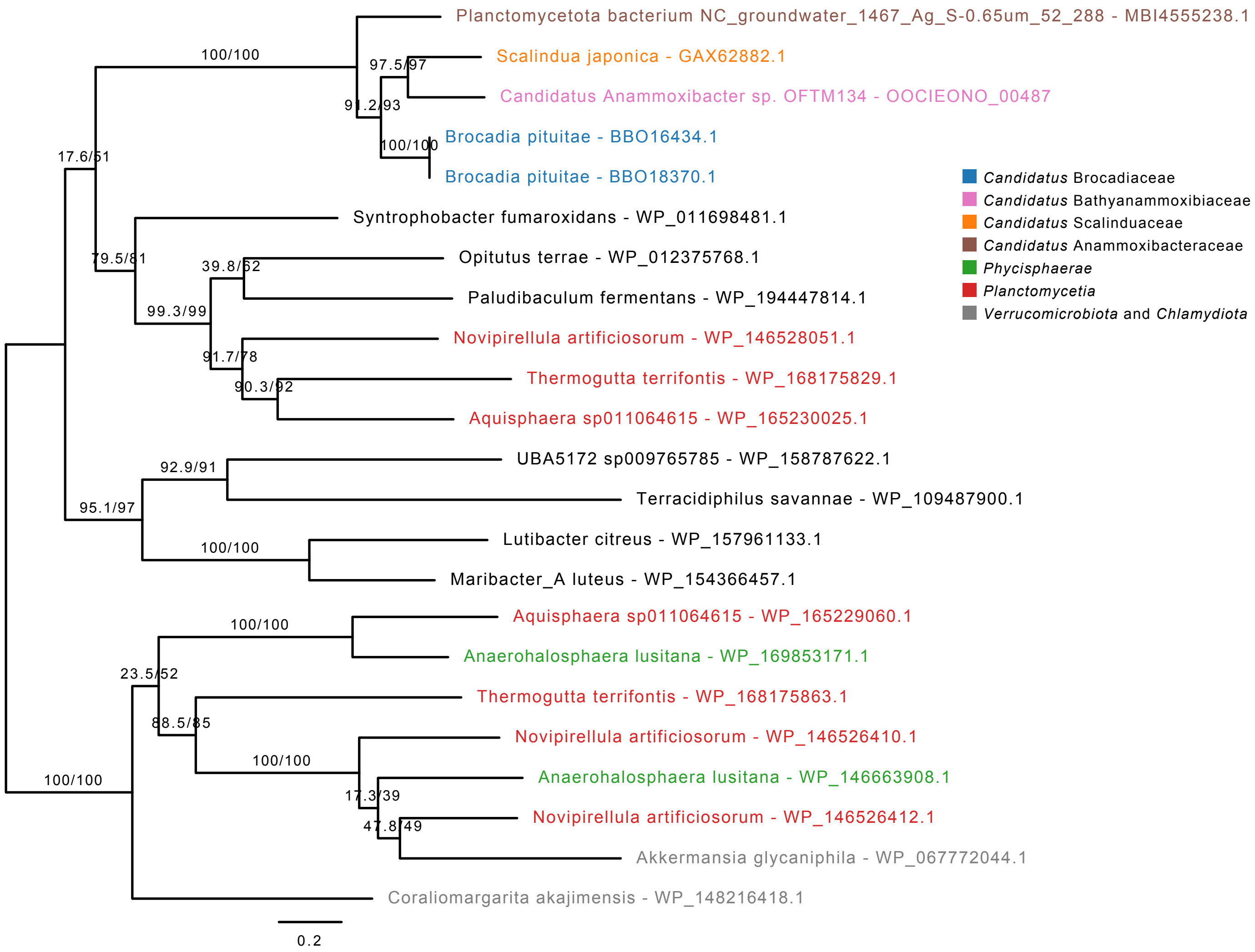

Supplementary Figure S5B

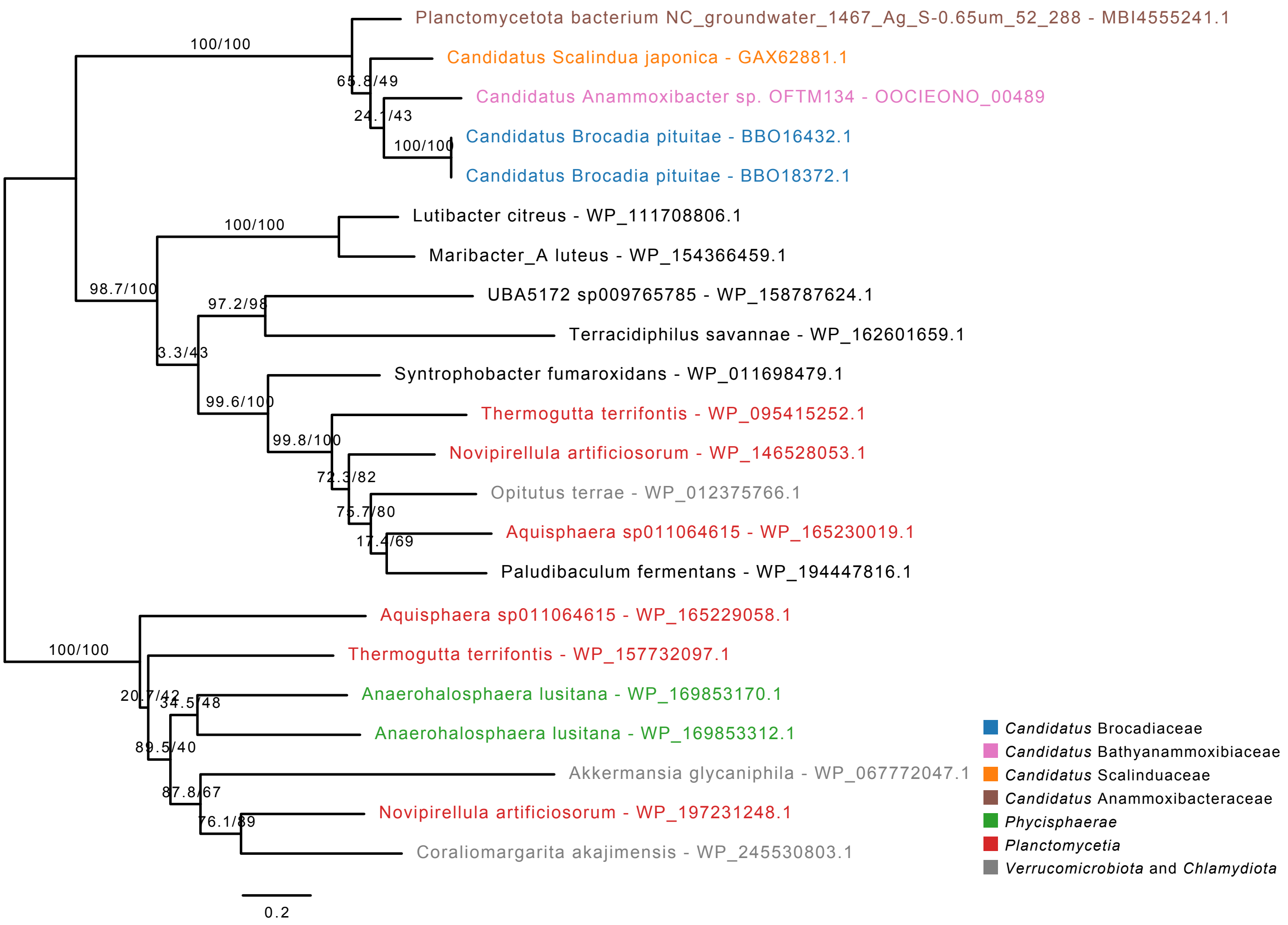

Supplementary Figure S5

C

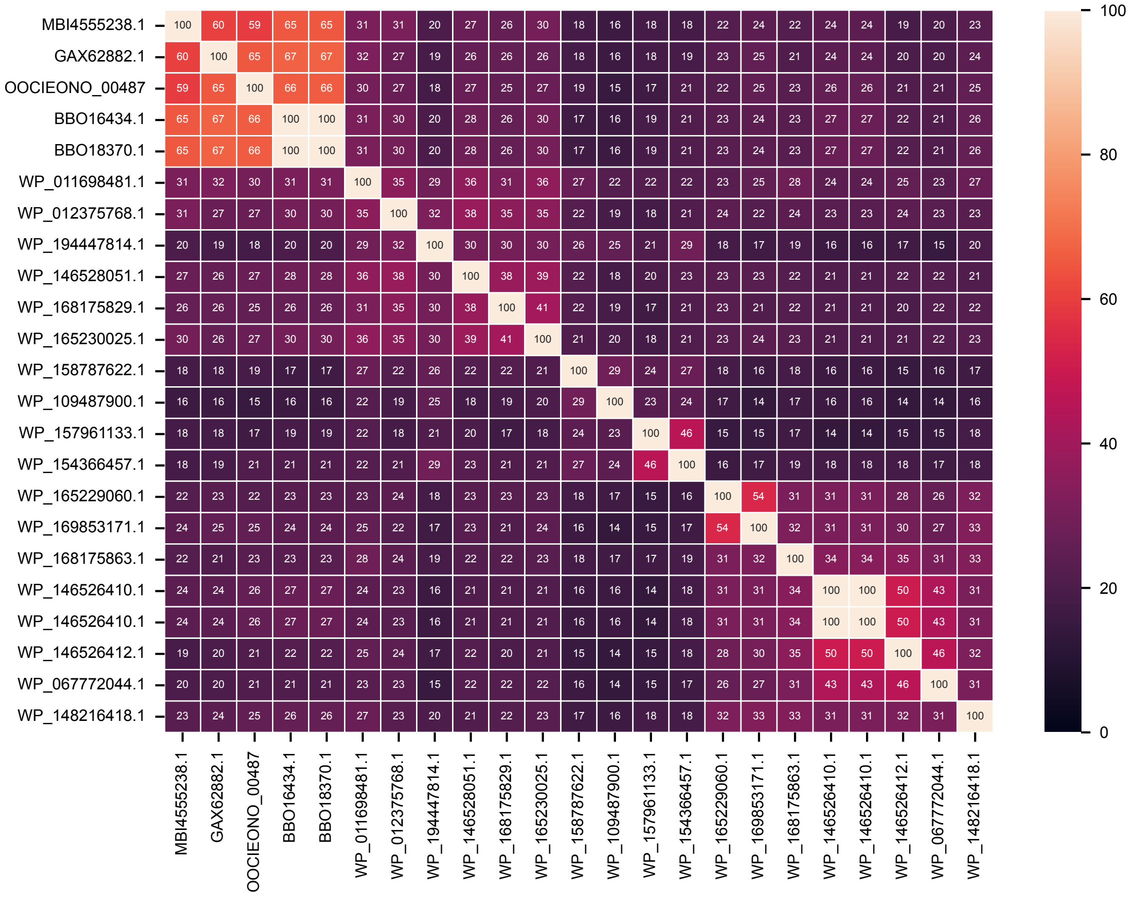

D

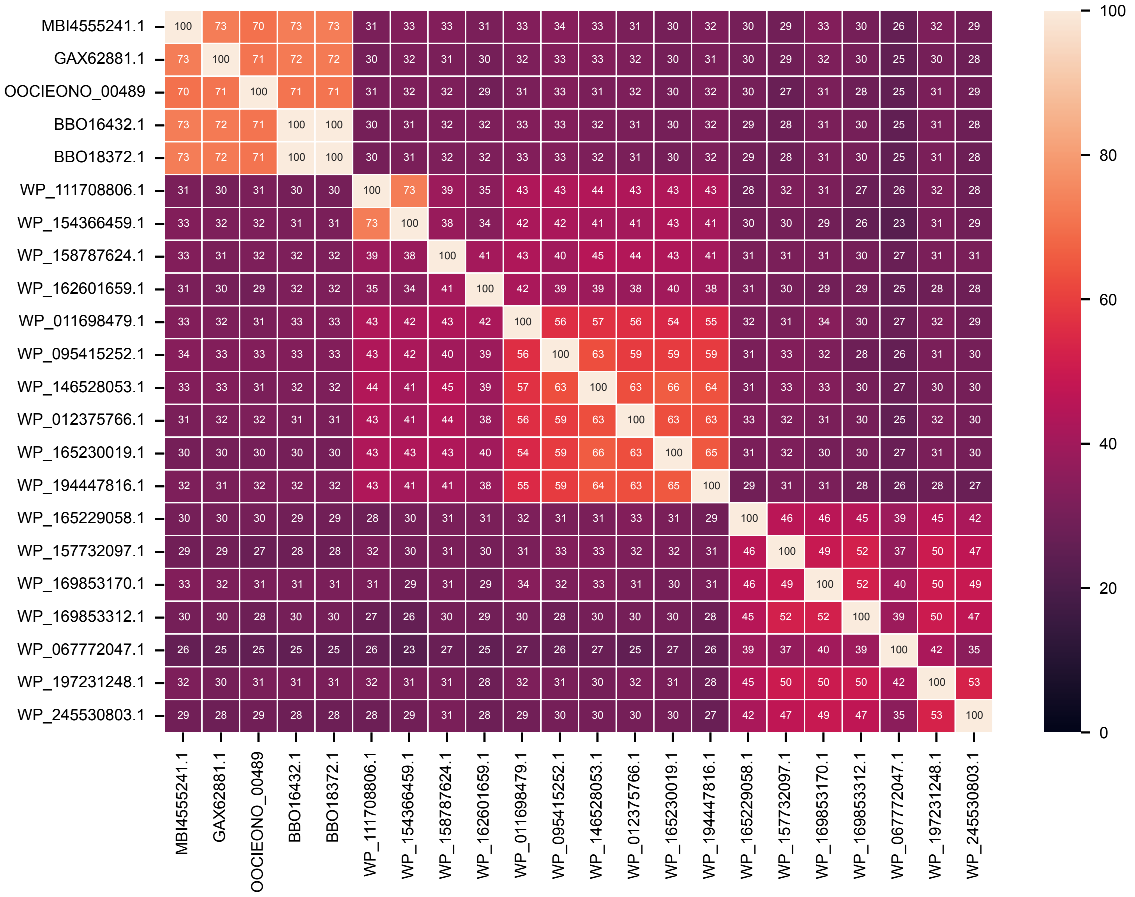

Supplementary Figure S6

A

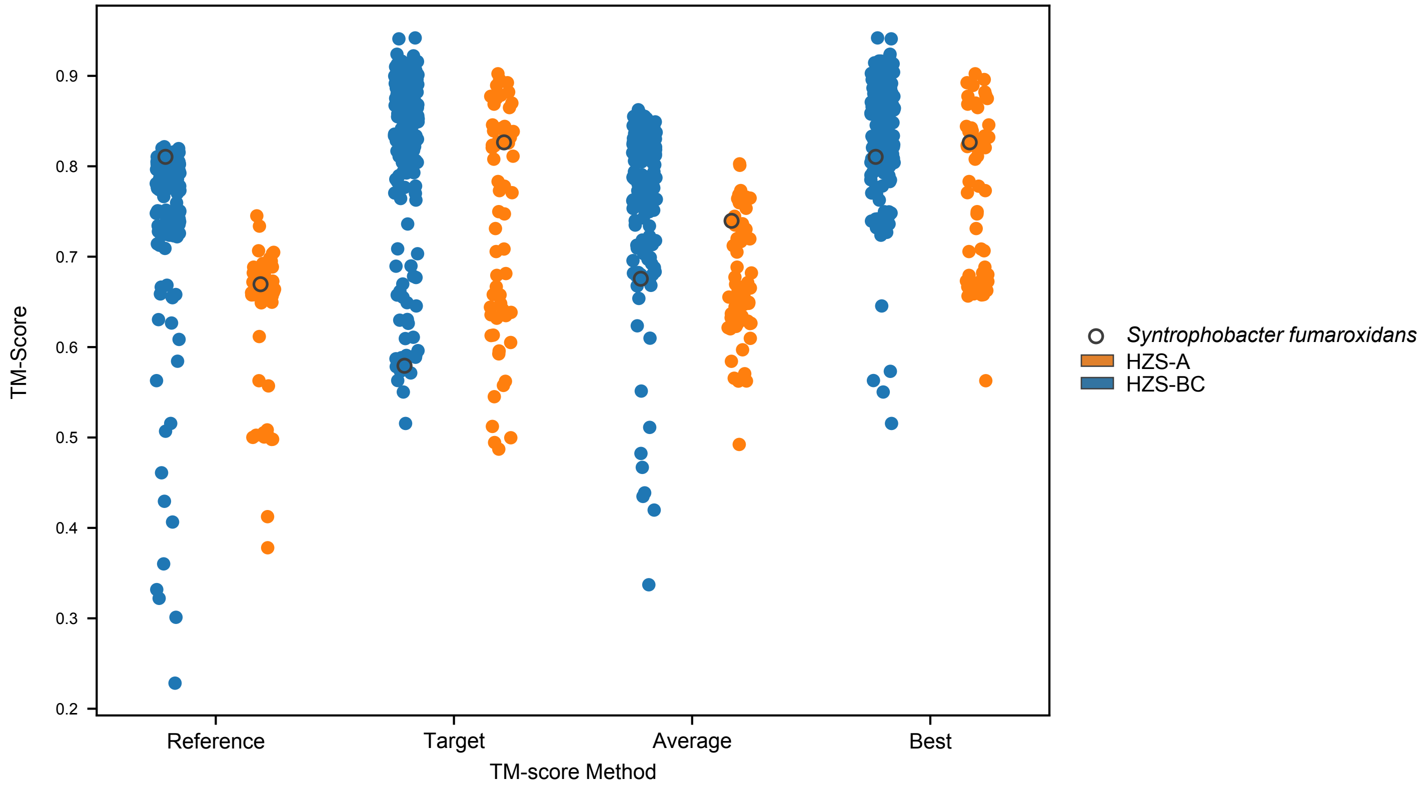

B

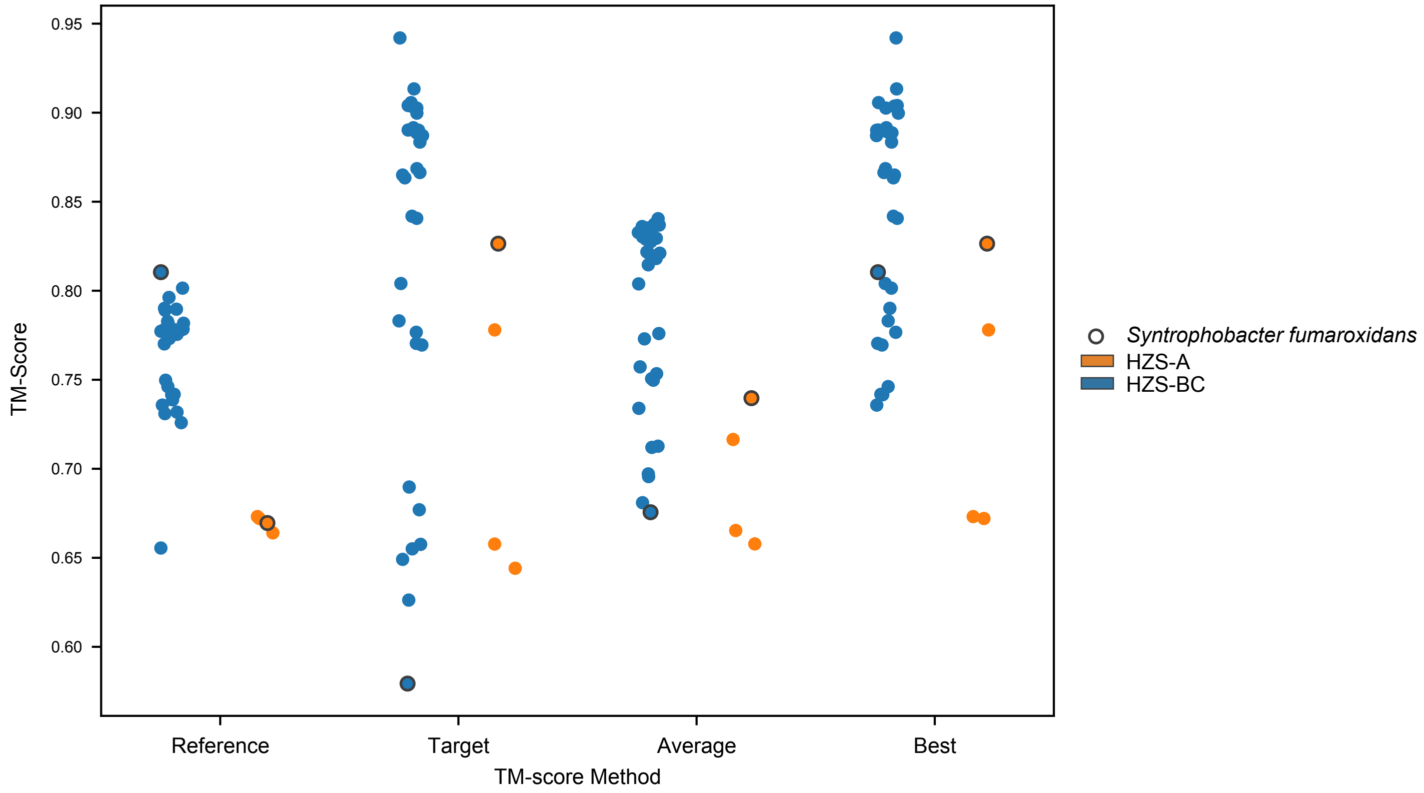

Supplementary Figure S7A

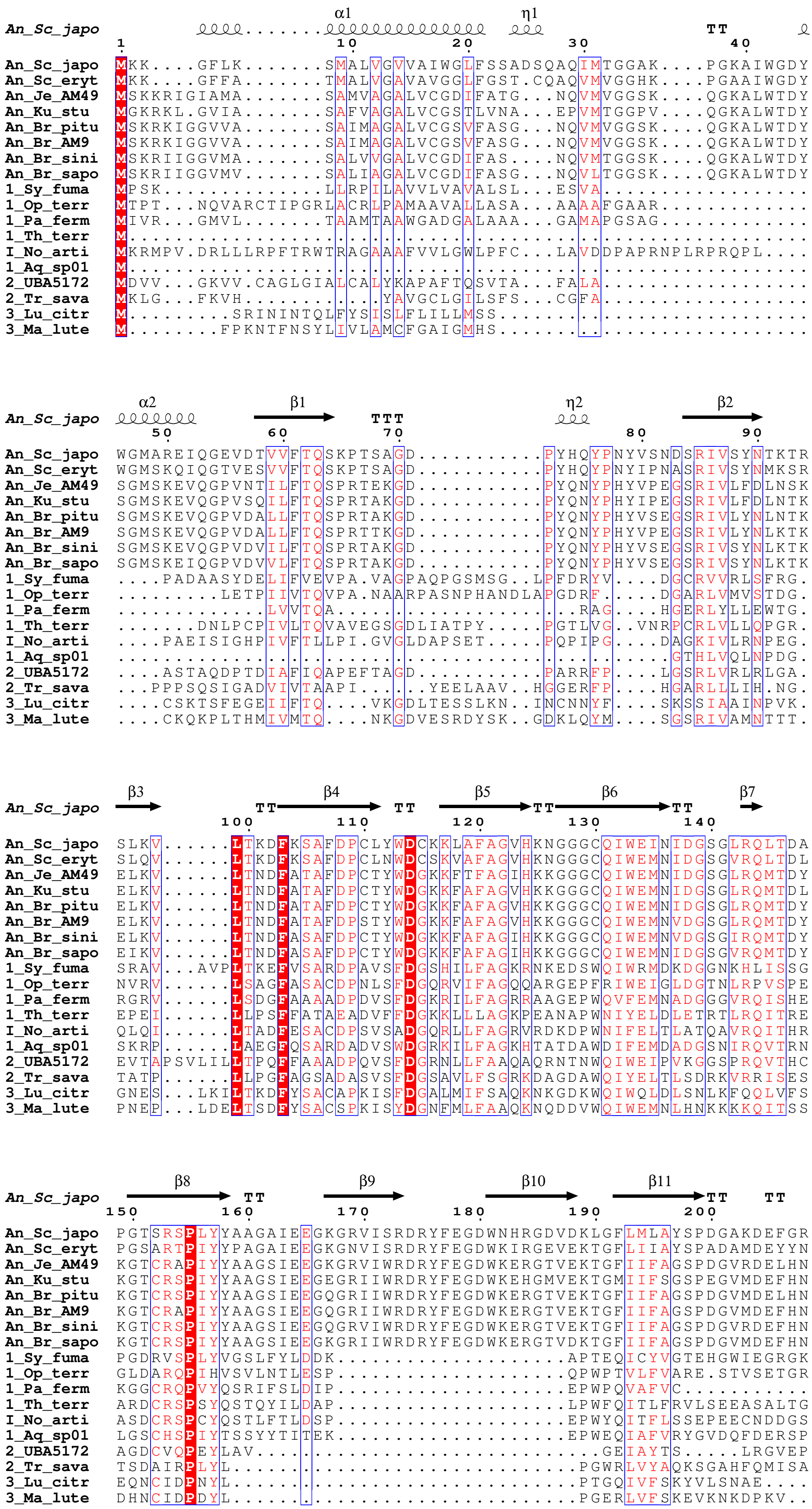

```
                 β12            α3            β13 η3  β14          β15
An_Sc_japo                                                                TT
              210         220         230         240         250         260
An_Sc_japo    DHNFDIYRLDPQ.GGKSMDRICGHLLVGLTMPNVDTVMDKITYNASSNFDPTLTRDGNIM
An_Sc_eryt    NYSYSLYRLDPQGGGKMMDRICGHKLMGIDMPSTNSVVDRINYNVSSDFDPALTRDGNIM
An_Je_AM49    PYVYNLFRLDTQ.GGHVTERITGHVLSGIEFPSLNTTIDQITYNLSSNFDPWLTPDGNIL
An_Ku_stu     PYAYNLYRLDTQ.GGKIIQRITGHVLSGIEFPHLNTTIDQITYNLSSNFDPWLTPDGNIL
An_Br_pitu    PYAYNLFRLDTQ.GGHVTERITGHVLSGIEFPSINTTIDQITYNISSNFDPTTTPDGNIL
An_Br_AM9     PYAYNLFRLDTQ.GGHVTERITGHVLSGIEFPSINTTIDQITYNISSNFDPTTTPDGNIL
An_Br_sini    PYAYNLFRLDTQ.GGHVTERITGHVLSGIEFPHLNTSIDQITYNVSSNYDPALTPDGNIL
An_Br_sapo    PYAYNLFRLDTQ.GGHVMERITGHVLSGIEFPNLNTTIDQITYNVSSNYDPTLTPDGNIL
1_Sy_fuma     APTLALYAANMD.GS...................KARRIAYNPFSDFDPDVLPDGRLV
1_Op_terr     PSVSNLYDVKLD.GT...................ELRRLTYNPNYNLDPVQMWDGRVI
1_Pa_ferm     ..GGALYSSKLD.GS...................DVQRLTYAGQSDTGPAMLPEGRMI
1_Th_terr     TAVSSLFSCRMD.GS...................HFRRLTVSHAPDLPGTLLPDGRIV
I_No_arti     SPAAHLYSCKLD.GT...................TIRRLTFNLSSDLDPWLMSDGRLL
1_Aq_sp01     NPATALWTCKLD.GS...................YPQRITYNLSSDLDPAILPDGRIA
2_UBA5172     ARTSEVQICRND.GS...................EAHPITFGPGRYEVEAVLRSGRLL
2_Tr_sava     RLADTEAIQEIE.GKGV...............PTAFPISFAAVSAIPSDVMADGRIL
3_Lu_citr     NGGYALFTCNFD.GS...................ELHQITFNPHSYFSSIVLKDGRLL
3_Ma_lute     DTEFTLFSCNLD.GS...................NLAQVTYDPSTYFASTVLSEGRVI
```

```
              β16                β17             β18             β19              β20
An_Sc_japo                             TT              TT   TT          TT  ...
              270         280         290         300         310         320
An_Sc_japo    FSSTQGNGTHNNSKGSTCLLVDNWDGSYPRHIYGNEVDEQPNDPKVSAREASDGY...VY
An_Sc_eryt    FSSTQANGPRNGGKGSICLVVDNWDGSYPRHIYGNEANEQADTAKVQAREAVDGN...IY
An_Je_AM49    FSSVQANGTRAEGKGRVMLCVDNWDGAYPRPIYGNCDGEIGGSSGRSQSKVTFGD.RKLV
An_Ku_stu     FSSVQANGSRAGGEGRVMICVDNWDGAYPRPIYGNCDGEIGGTSGRSQAKITFGD.RKIV
An_Br_pitu    FSSTQANGSRAGGKGRVMLCVDNWDGAYPRPIYGNCDDEIGGACGKSQAKITFGE.RKLV
An_Br_AM9     FSSTQANGSRAGGKGRVMLCVDNWDGAYPRPIYGNCEGEVGGASGRSQAKITFGD.RRLV
An_Br_sini    FSSVQANGSRAEGKGRVLLEVDNWDGAYPRAIYGNCPDEIGGACGRSQAKITFGD.RKLV
An_Br_sapo    FSSVQANGSRAEGKGRVLLEVDNWDGAYPRAIYGNCPDEIGGACGRSQAKITFGD.RKLV
1_Sy_fuma     YSSRQPTEPGSGMREMTQLFAVNIDGSDAMAYAGDS..EPPSHMEMARVDWKGSR...VY
1_Op_terr     YAAERYANDASAEPGRVGLYAIHIEGADMERYGG....ELGRRIQRTPCPTADGL...LV
1_Pa_ferm     YSSGGR...........LFGINLDGTDYALFTG....GLNAR.......TAAAVGRKVV
1_Th_terr     FASFRSSYPHDPQV.RLDLLAVNTDGTDPANFLP....GIGARYKLMPTVIPHGLLGKLV
I_No_arti     HASWQRARLDHGNSGYMGLFAVNIDGIDFSRYGQ....QAGKRFKRMPCVGDHGL...VL
1_Aq_sp01     YASWRRADFQDGTAGRLALETLNTDGTDRAPAV.....RRDDVARRMPCVTPAGD...LI
2_UBA5172     LSAEAPLVDVPSVNQARSLYLVDPDGSGLMLLRQD...GTAQAIRSGAVELTDGT...IL
2_Tr_sava     FESGFPL....GEGGTPELYLAYSDGSGVESYRCDH.PTKELDARYGGHQLTSGD...VV
3_Lu_citr     AISKQYF....PEDKKGVLMISRPDGSKQDVFYKS...SNTYNLISIGCETDKGE...IL
3_Ma_lute     AIGKELW....PEEKPANFMIMRPDGTKKELFYQM...EQDHYITSGATEMNNGR...IL
```

```
                               β21           η4 β22          β23          β24
An_Sc_japo           TT                                            TT
                 330         340         350         360         370
An_Sc_japo    YIEALDCNS.GIGNLARVSWTTPHSKTQSRLNH.DGRLYRSPHPMPDGRLMVSSAER...
An_Sc_eryt    YIEALDYDS.GVGNLAKVSWTTPHAKTQSRINR.DGRLYRSPFPLPDGRLMVSSSER...
An_Je_AM49    FVESPYMNW.GVGQLAAVSWDAPFNKTYERLSKDEGGLYRSPYPLPDDRMLVSYAQR...
An_Ku_stu     YVESPYMNW.GVGQLAAVSWDAPFNKTYEKLTGKDGGLYRSPYPLPDDRMLVSYAER...
An_Br_pitu    YVESPYMNW.GVGQLASVSWDAPYNKTYERLTKDDGGLYRSPYPLPDDRMLVSYAER...
An_Br_AM9     YVESPYMNW.GVGQLASVSWDAPYNKTYERLTKDDGGLYRSPYPLPDDRMLVSYAER...
An_Br_sini    YVESPYMNW.GVGQLAAVSWDAPYNKTYERLTKDDGGLYRSPYPLPDDRMLVSYAER...
An_Br_sapo    FVESPYMNW.GVGQLAAVSWDAPYNKTYERLTKDDGGLYRSPYPLPDDRMLVSYAER...
1_Sy_fuma     FIESRVHTWPGGGALAFVSQRRPYH.SRAVLGANEKGFFHSPCPLPDGGLLVSHRANR.P
1_Op_terr     FVESDQPTADGSGQLACVEQRRPHL.TYRSLTNDADFVYAHPSPLGGNRVLVAQRPHR.A
1_Pa_ferm     FVEA.......AGQLSMVTQERPLH.SHSALTAAADGVFAAPAALGEGSVVVAKKGP...
1_Th_terr     FIESDELTPDGAGQIACVDLRRPLR.TYQRLTEDRAWVYHSPCALPDGRLLVSRRPRT.G
I_No_arti     FVESDDRRRDGSGCLGSVTERRPLH.SYQSLTTPADGRYHSPSPLPGGKFLVSHRAAGDS
1_Aq_sp01     FVEPHDRSN.GPGRLAGVSLRRPLH.TYMVLTGPRDGLFHSPAPLPDGSLVVEWFPPKDG
2_UBA5172     FMQRNAASA.SEEHLAWI...HPGA.LQATAIESAHSGYASANALDHGTLLVSRRAP...
2_Tr_sava     FTH........GARLARFS..SPMA.QEEGIPAPPADYAGRIAEAGSGDWLFSVRKSV..
3_Lu_citr     FIEQNTSTE..EYRIVSVNYSNPLN.TRVELSKTIKGSFHFVNSKNDTEFVTSFKPSS..
3_Ma_lute     FVEANSNNQ...KDLISLSYNRPLH.SEENLTSEIKGDFHAISPYHDNKVLSSYRPEN..
```

```
                     β25  η5   β26         β27                β28  β29
An_Sc_japo                            TT                        TT       TTT
              380         390         400         410         420         430
An_Sc_japo    .QDFGIYYFCADKGTVSELVYDDPEWNDHQPQPVYPRYKPRWINSFTAGAEFGVTTVTYQ
An_Sc_eryt    .QDFGLFWFDHSKGTVSEKVYDDPEWNDHQPQPVYPRYKPRWINSFTAGKEFGVTTVTYQ
An_Je_AM49    .GDFGIYWFDCKNGRAGEVVHNDPEWNDHQPAPVYIKYKPRWINTFTAGKDFGVTTVTYQ
An_Ku_stu     .GDFGIYWFNFSKCAAGDKVYDDPNWNDHQPAPVYVKYKPRWINTFTAGKNFGVTVVTYQ
An_Br_pitu    .GDFGIYWFDCKNGKAGELVHNDPEWNDHQPAPIYVKYKPRWINTFTAGKNFGVTTVTYQ
An_Br_AM9     .GDFGIYWFDFKNGKAGELVHNDPEWNDHQPAPIYVKYRPRWINTFTAGKNFGVTTVTYQ
An_Br_sini    .GDFGIYWFDCKNGKAGELVYNDPEWNDHQPAPVYIKYKPRWINTFTAGKNFGVTTVTYQ
An_Br_sapo    .GDFGIYWFDCKNGKAGELVYNDPEWNEHQPAPVYVKYRPRWINTFTAGKNFGVTVVTYQ
1_Sy_fuma     GAVFGIHRIDPGTGKLLKAVYSSPGRHCFGTRILAPHPAAKGRSSVVGFR........Y.
1_Op_terr     KATWGIVAFDATTGR.SESVFDMGDFDEVQAVLVKPRPSPDGHSTVVVSK..........
1_Pa_ferm     .AGWGLWRLELATRK.QTPLYDSPEFDELQAKVVGPHEQPDGRGTVVETE..........
1_Th_terr     GSSFALGIFDLEARA.WTPLFDDPRYHELLAKPVAPRLLPDGRSSGVDDN..........
I_No_arti     TSSYGIYRYDPNQGR.RERLVDDPAVHEVQARVIRPSQRADGRSTVVDDL..........
1_Aq_sp01     GGTPGLVRLDLAART.IEPILLPVEGKAIQPKAIAPRPRPDGRSSVVSPE..........
2_UBA5172     GHRFDLYQIFPNSRGRDQLLYRDAHASSVQAVLIAPHTVALTYSSILHPD..........
2_Tr_sava     TDSFALKLWRPGSPG.LRSFFAQTGSNLIDPVLVAARNRPRRHPSALHDW..........
3_Lu_citr     NDRYGIYEYNSIKNAIEKEIYTNSDFNIIDVVLVEKRELPRKLPSEINLN..........
3_Ma_lute     ALHFALYEFDVESRELGRPIYEDGENNILEAVFVQKKQRPRKLPSEVDLN..........
```

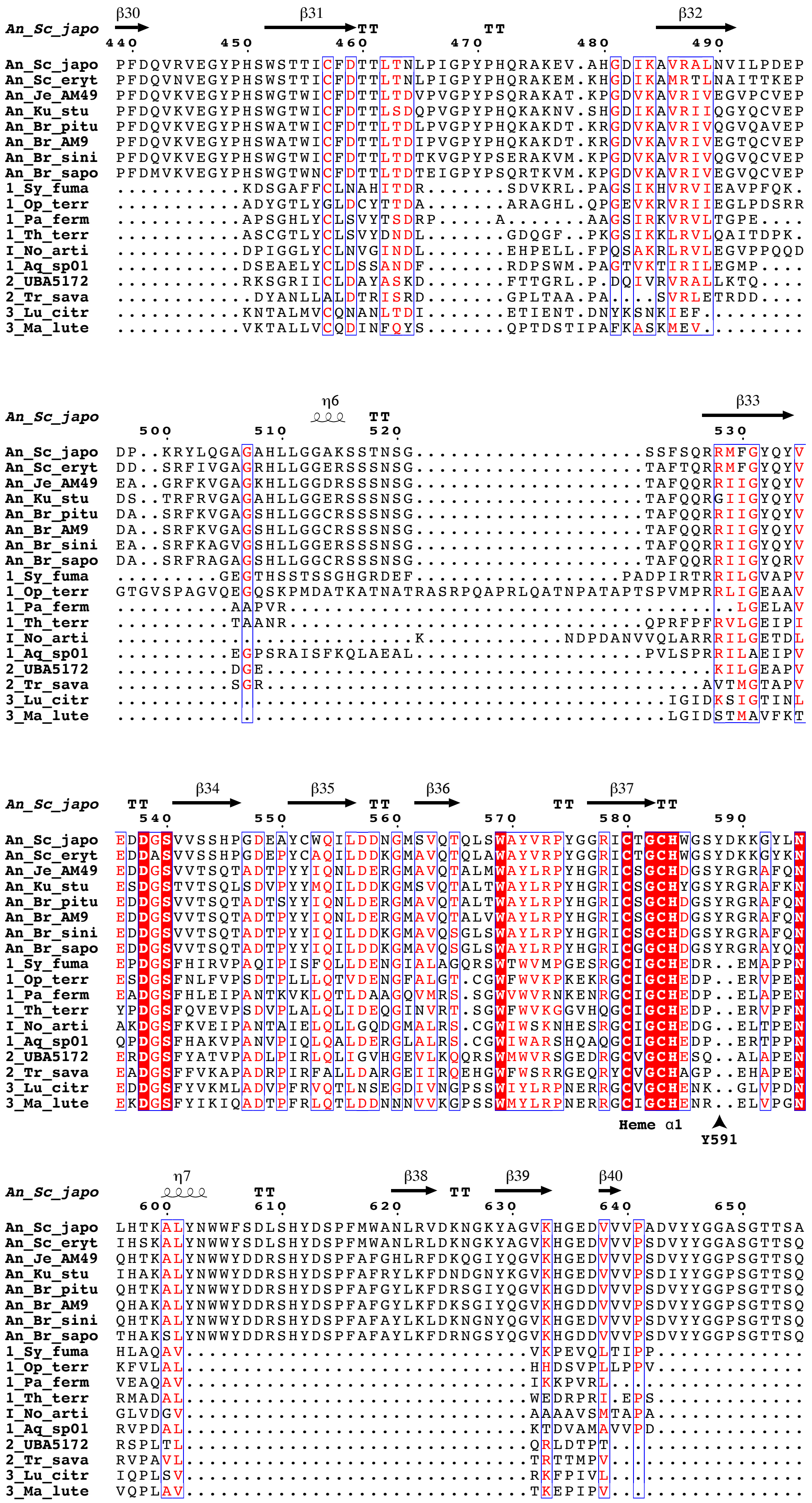

An_Sc_japo β30 β31 TT TT β32
440 450 460 470 480 490
An_Sc_japo PFDQVRVEGYPHSWSTTICFDTTLTNLPIGPYPHQRAKEV.AHGDIKAVRALNVILPDEP
An_Sc_eryt PFDQVNVEGYPHSWSTTICFDTTLTNLPIGPYPHQRAKEM.KHGDIKAMRTLNAITTKEP
An_Je_AM49 PFDQVKVEGYPHSWGTWICFDTTLTDVPVGPYPSQRAKAT.KPGDVKAVRIVEGVPCVEP
An_Ku_stu PFDQVKVEGYPHSWGTWICFDTTLSDQPVGPYPHQKAKNV.SHGDIKAVRIIQGYQCVEP
An_Br_pitu PFDQVKVEGYPHSWATWICFDTTLTDLPVGPYPHQKAKDT.KRGDVKAVRIVQGVQAVEP
An_Br_AM9 PFDQVKVEGYPHSWATWICFDTTLTDIPVGPYPHQKAKDT.KRGDVKAVRIVEGTQCVEP
An_Br_sini PFDQVKVEGYPHSWGTWICFDTTLTDTKVGPYPSERAKVM.KPGDVKAVRIVQGVQCVEP
An_Br_sapo PFDMVKVEGYPHSWGTWNCFDTTLTDTEIGPYPSQRTKVM.KPGDVKAVRIVEGVQCVEP
1_Sy_fuma ...........KDSGAFFCLNAHITDR.......SDVKRL.PAGSIKHVRVIEAVPFQK.
1_Op_terr ...........ADYGTLYCLDCYTTDA.......ARAGHL.QPGEVKRVRIIEGLPDSRR
1_Pa_ferm ...........APSGHLYCLSVYTSDRP.....A.......AAGSIRKVRVLTGPE....
1_Th_terr ...........ASCGTLYCLSVYDNDL.......GDQGF..PKGSIKKLRVLQAITDPK.
I_No_arti ...........DPIGGLYCLNVGINDL.......EHPELL.FPQSAKRLRVLEGVPPQQD
1_Aq_sp01 ...........DSEAELYCLDSSANDF.......RDPSWM.PAGTVKTIRILEGMP....
2_UBA5172 ...........RKSGRIICLDAYASKD.......FTTGRL.P.DQIVRVRALLKTQ....
2_Tr_sava ............DYANLLALDTRISRD.......GPLTAA.PA....SVRLETRDD....
3_Lu_citr ...........KNTALMVCQNANLTDI.......ETIENT.DNYKSNKIEF.........
3_Ma_lute ...........VKTALLVCQDINFQYS.......QPTDSTIPAFKASKMEV.........
An_Sc_japo η6 TT β33
500 510 520 530
An_Sc_japo DP..KRYLQGAGAHLLGGAKSSTNSG....................SSFSQRRMFGYQYV
An_Sc_eryt DD..SRFIVGAGRHLLGGERSSSNSG....................TAFTQRRMFGYQYV
An_Je_AM49 EA..GRFKVGAGKHLLGGDRSSSNSG....................TAFQQRRIIGYQYV
An_Ku_stu DS..TRFRVGAGAHLLGGERSSSNSG....................TAFQQRGIIGYQYV
An_Br_pitu DA..SRFKVGAGSHLLGGCRSSSNSG....................TAFQQRRIIGYQYV
An_Br_AM9 DA..SRFKVGAGSHLLGGCRSSSNSG....................TAFQQRRIIGYQYV
An_Br_sini EA..SRFKAGVGSHLLGGERSSSNSG....................TAFQQRRIIGYQYV
An_Br_sapo DA..SRFRAGAGSHLLGGCRSSSNSG....................TAFQQRRIIGYQRV
1_Sy_fuma .........GEGTHSSTSSGHGRDEF..................PADPIRTRRILGVAPV
1_Op_terr GTGVSPAGVQEGQSKPMDATKATNATRASRPQAPRLQATNPATAPTSPVMPRRLIGEAAV
1_Pa_ferm ....AAPVR.......................................LGELAV
1_Th_terr ....TAANR...............................QPRFPFRVLGEIPI
I_No_arti ....K............NDPDANVVQLARRRILGETDL
1_Aq_sp01 ..........EGPSRAISFKQLAEAL....................PVLSPRRILAEIPV
2_UBA5172 ....DGE.......................................KILGEAPV
2_Tr_sava ....SGR......................................AVTMGTAPV
3_Lu_citr ....IGIDKSIGTINL
3_Ma_lute ....LGIDSTMAVFKT
An_Sc_japo TT β34 β35 TT β36 TT β37 TT
540 550 560 570 580 590
An_Sc_japo EDDGSVVSSHPGDEAYCWQILDDNGMSVQTQLSWAYVRPYGGRICTGCHWGSYDKKGYLN
An_Sc_eryt EDDASVVSSHPGDEPYCAQILDDKGMAVQTQLAWAYVRPYGGRICTGCHWGSYDKKGYKN
An_Je_AM49 EDDGSVVTSQTADTPYYIQNLDERGMAVQTALMWAYLRPYHGRICSGCHDGSYRGRAFQN
An_Ku_stu ESDGSTVTSQLSDVPYYMQILDDKGMSVQTALTWAYLRPYHGRICSGCHYGSYRGRAFKN
An_Br_pitu EDDGSVVTSQTADTSYYIQNLDERGMAVQTALTWAYLRPYHGRICSGCHDGSYRGRAFQN
An_Br_AM9 EDDGSVVTSQTADTPYYIQNLDERGMAVQTALVWAYLRPYHGRICSGCHDGSYRGRAFQN
An_Br_sini EDDGSVVTSQTADTPYYIQILDDKGMAVQSGLSWAYLRPYHGRICSGCHDGSYRGRAFQN
An_Br_sapo EDDGSVVTSQTADTPYYIQILDDKGMAVQSGLSWAYLRPYHGRICGGCHDGSYRGRAYQN
1_Sy_fuma EPDGSFHIRVPAQIPISFQLLDENGIALAGQRSWTWVMPGESRGCIGCHEDR..EMAPPN
1_Op_terr ESDGSFNLFVPSDTPLLLQTVDENGFALGT.CGWFWVKPKEKRGCIGCHEDP..ERVPEN
1_Pa_ferm EADGSFHLEIPANTKVKLQTLDAAGQVMRS.SGWVWVRNKENRGCIGCHEDP..ELAPEN
1_Th_terr YPDGSFQVEVPSDVPLALQLIDEQGINVRT.SGWFWVKGGVHQGCIGCHEDP..ERVPFN
I_No_arti AKDGSFKVEIPANTAIELQLLGQDGMALRS.CGWIWSKNHESRGCIGCHEDG..ELTPEN
1_Aq_sp01 QPDGSFHAKVPANVPIQLQALDERGLALRS.CGWIWARSHQAQGCIGCHEDP..ERTPPN
2_UBA5172 ERDGSFYATVPADLPIRLQLIGVHGEVLKQQRSWMWVRSGEDRGCVGCHESQ..ALAPEN
2_Tr_sava EADGSFFVKAPADRPIRFALLDARGEIIRQEHGWFWSRRGEQRYCVGCHAGP..EHAPEN
3_Lu_citr EDDGSFYVKMLADVPFRVQTLNSEGDIVNGPSSWIYLRPNERRGCVGCHENK..GLVPDN
3_Ma_lute EKDGSFYIKIQADTPFRLQTLDDNNNVVKGPSSWMYLRPNERRGCIGCHENR..ELVPGN
Heme α1
Y591
An_Sc_japo η7 TT β38 TT β39 β40
600 610 620 630 640 650
An_Sc_japo LHTKALYNWWFSDLSHYDSPFMWANLRVDKNGKYAGVKHGEDVVVPADVYYGGASGTTSA
An_Sc_eryt IHSKALYNWWYSDLSHYDSPFMWANLRLDKNGKYAGVKHGEDVVVPSDVYYGGASGTTSQ
An_Je_AM49 QHTKALYNWWYDDRSHYDSPFAFGHLRFDKQGIYQGVKHGEDVVVPSDVYYGGPSGTTSQ
An_Ku_stu IHAKALYNWWYDDRSHYDSPFAFRYLKFDNDGNYKGVKHGEDVVVPSDIYYGGPSGTTSQ
An_Br_pitu QHTKALYNWWYDDRSHYDSPFAFGYLKFDRSGIYQGVKHGDDVVVPSDVYYGGPSGTTSQ
An_Br_AM9 QHAKALYNWWYDDRSHYDSPFAFGYLKFDKSGIYQGVKHGDDVVVPSDVYYGGPSGTTSQ
An_Br_sini QHTKALYNWWYDDRSHYDSPFAFAYLKLDKNGNYQGVKHGEDVVVPSDVYYGGPSGTTSQ
An_Br_sapo THAKSLYNWWYDDRSHYDSPFAFAYLKFDRNGSYQGVKHGDDVVVPSDVYYGGPSGTTSQ
1_Sy_fuma HLAQAV..............................VKPEVQLTIPP.............
1_Op_terr KFVLAL..............................HHDSVPLLPPV.............
1_Pa_ferm VEAQAV..............................IKKPVRL.................
1_Th_terr RMADAL..............................WEDRPRI.EPS.............
I_No_arti GLVDGV..............................AAAAVSMTAPA.............
1_Aq_sp01 RVPDAL..............................KTDVAMAVVPD.............
2_UBA5172 RSPLTL..............................QRLDTPT.................
2_Tr_sava RVPAVL..............................TRTTMPV.................
3_Lu_citr IQPLSV..............................RKFPIVL.................
3_Ma_lute VQPLAV..............................TKEPIPV.................

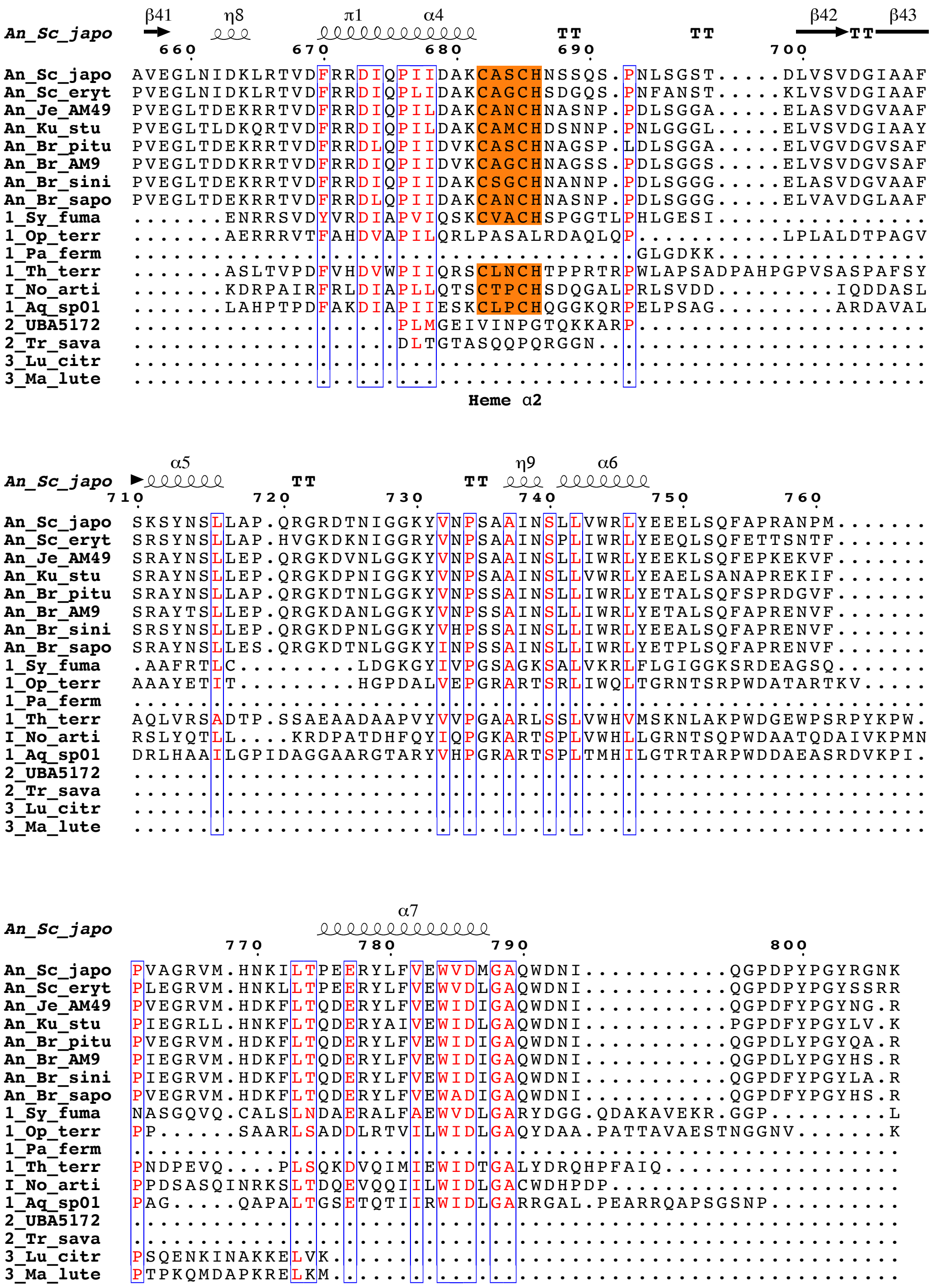

An_Sc_japo β41 η8 π1 α4 TT TT β42 TT β43
660 670 680 690 700
An_Sc_japo AVEGLNIDKLRTVDFRRDIQPIIDAKCASCHNSSQS.PNLSGST.....DLVSVDGIAAF
An_Sc_eryt PVEGLNIDKLRTVDFRRDIQPLIDAKCAGCHSDGQS.PNFANST.....KLVSVDGIAAF
An_Je_AM49 PVEGLTDEKRRTVDFRRDIQPILDAKCANCHNASNP.PDLSGGA.....ELASVDGVAAF
An_Ku_stu PVEGLTLDKQRTVDFRRDIQPILDAKCAMCHDSNNP.PNLGGGL.....ELVSVDGIAAY
An_Br_pitu PVEGLTDEKRRTVDFRRDLQPIIDVKCASCHNAGSP.LDLSGGA.....ELVGVDGVSAF
An_Br_AM9 PVEGLTDDKRRTVDFRRDIQPIIDVKCAGCHNAGSS.PDLSGGS.....ELVSVDGVSAF
An_Br_sini PVEGLTDEKRRTVDFRRDIQPIIDAKCSGCHNANNP.PDLSGGG.....ELASVDGVAAF
An_Br_sapo PVEGLTDEKRRTVDFRRDLQPIIDAKCANCHNASNP.PDLSGGG.....ELVAVDGLAAF
1_Sy_fuma .......ENRRSVDYVRDIAPVIQSKCVACHSPGGTLPHLGESI...............
1_Op_terr .......AERRRVTFAHDVAPILQRLPASALRDAQLQP...........LPLALDTPAGV
1_Pa_ferm ....GLGDKK................
1_Th_terr .......ASLTVPDFVHDVWPIIQRSCLNCHTPPRTRPWLAPSADPAHPGPVSASPAFSY
I_No_arti .......KDRPAIRFRLDIAPLLQTSCTPCHSDQGALPRLSVDD.........IQDDASL
1_Aq_sp01 .......LAHPTPDFAKDIAPIIESKCLPCHQGGKQRPELPSAG.........ARDAVAL
2_UBA5172 ..................PLMGEIVINPGTQKKARP.......................
2_Tr_sava ..................DLTGTASQQPQRGGN..........................
3_Lu_citr ....
3_Ma_lute ....
Heme α2
An_Sc_japo α5 TT TT η9 α6
710 720 730 740 750 760
An_Sc_japo SKSYNSLLAP.QRGRDTNIGGKYVNPSAAINSLLVWRLYEEELSQFAPRANPM.......
An_Sc_eryt SRSYNSLLAP.HVGKDKNIGGRYVNPSAAINSPLIWRLYEEQLSQFETTSNTF.......
An_Je_AM49 SRAYNSLLEP.QRGKDVNLGGKYVNPSAAINSLLIWRLYEEKLSQFEPKEKVF.......
An_Ku_stu SRAYNSLLEP.QRGKDPNIGGKYVNPSAAINSLLVWRLYEAELSANAPREKIF.......
An_Br_pitu SRAYNSLLAP.QRGKDTNLGGKYVNPSSAINSLLIWRLYETALSQFSPRDGVF.......
An_Br_AM9 SRAYTSLLEP.QRGKDANLGGKYVNPSSAINSLLIWRLYETALSQFAPRENVF.......
An_Br_sini SRSYNSLLEP.QRGKDPNLGGKYVHPSSAINSLLIWRLYEEALSQFAPRENVF.......
An_Br_sapo SRAYNSLLES.QRGKDTNLGGKYINPSSAINSLLIWRLYETPLSQFAPRENVF.......
1_Sy_fuma .AAFRTLC.........LDGKGYIVPGSAGKSALVKRLFLGIGGKSRDEAGSQ.......
1_Op_terr AAAYETIT.........HGPDALVEPGRARTSRLIWQLTGRNTSRPWDATARTKV.....
1_Pa_ferm ....
1_Th_terr AQLVRSADTP.SSAEAADAAPVYVVPGAARLSSLVWHVMSKNLAKPWDGEWPSRPYKPW.
I_No_arti RSLYQTLL....KRDPATDHFQYIQPGKARTSPLVWHLLGRNTSQPWDAATQDAIVKPMN
1_Aq_sp01 DRLHAAILGPIDAGGAARGTARYVHPGRARTSPLTMHILGTRTARPWDDAEASRDVKPI.
2_UBA5172 ....
2_Tr_sava ....
3_Lu_citr ....
3_Ma_lute ....
An_Sc_japo α7
770 780 790 800
An_Sc_japo PVAGRVM.HNKILTPEERYLFVEWVDMGAQWDNI...........QGPDPYPGYRGNK
An_Sc_eryt PLEGRVM.HNKLLTPEERYLFVEWVDLGAQWDNI...........QGPDPYPGYSSRR
An_Je_AM49 PVEGRVM.HDKFLTQDERYLFVEWIDIGAQWDNI...........QGPDFYPGYNG.R
An_Ku_stu PIEGRLL.HNKFLTQDERYAIVEWIDLGAQWDNI...........PGPDFYPGYLV.K
An_Br_pitu PVEGRVM.HDKFLTQDERYLFVEWIDIGAQWDNI...........QGPDLYPGYQA.R
An_Br_AM9 PIEGRVM.HDKFLTQDERYLFVEWIDIGAQWDNI...........QGPDLYPGYHS.R
An_Br_sini PIEGRVM.HDKFLTQDERYLFVEWIDIGAQWDNI...........QGPDFYPGYLA.R
An_Br_sapo PVEGRVM.HDKFLTQDERYLFVEWADIGAQWDNI...........QGPDFYPGYHS.R
1_Sy_fuma NASGQVQ.CALSLNDAERALFAEWVDLGARYDGG.QDAKAVEKR.GGP.........L
1_Op_terr PP......SAARLSADDLRTVILWIDLGAQYDAA.PATTAVAESTNGGNV.......K
1_Pa_ferm ....
1_Th_terr PNDPEVQ....PLSQKDVQIMIEWIDTGALYDRQHPFAIQ..................
I_No_arti PPDSASQINRKSLTDQEVQQIILWIDLGACWDHPDP......................
1_Aq_sp01 PAG.....QAPALTGSETQTIIRWIDLGARRGAL.PEARRQAPSGSNP..........
2_UBA5172 ....
2_Tr_sava ....
3_Lu_citr PSQENKINAKKELVK...
3_Ma_lute PTPKQMDAPKRELKM...

Supplementary Figure S7B

An_Sc_japo

```
                          1                   10        20
                          .                    .         .
An_Sc_japo   .............MKKKAW......ILGFLSAGILSLAL......................
An_Sc_eryt   .............MKKRSL......FLSILGAGLVSLLI......................
An_Je_AM49   .............MKGRGL.......IGALVIGVSVMVG......................
An_Ku_stu    .............MIRKGM.......IGAVMLGAAVAIS......................
An_Br_pitu   .............MKGRVW.......LGAAVVG.AVLSV......................
An_Br_AM9    .............MKGRGW.......IGAAVVG.AVLSV......................
An_Br_sini   .............MKERGW.......IGALVLG.AVMVG......................
An_Br_sapo   .............MKGRGW.......IGAVVLG.FVMAG......................
1_Sy_fuma    M............SRTSRIFQLS..LFPGLSLLLLFVLGASAARQGDPAEPPARAKVQAT
1_Op_terr    .............MRLTL.........NLLAALATFAVVP...........LTTRAADSYA
1_Pa_ferm    .............MR............PRTIAVLAAGLV................ALLGMG
1_No_arti    MS...........TRITT.........PTSLLILIWGVAA.................LSSA
1_Th_terr    MSTAGFRRT....LRSSGKGSSWVRRIPWILIGISVSVLA...............ARDSKA
1_Aq_sp01    M............IRGTNDKGFE...RPPIRPLLAIAAAS...........ILTWAWPMSV
2_UBA5172    MTTASKRSASRVRRRDEALLGIA...LLSVIIALIMFIAQ.....................
2_Tr_sava    MSL..........LRSGSARAAA...VTAVVLVCSWAIAV.....................
3_Lu_citr    .............MKKTRL....L.ILFGIILLLVVIIAY.....................
3_Ma_lute    .............MKSKKV....K.IFFFVIAFLGLIIFI.....................
```

An_Sc_japo

```
An_Sc_japo   .............................................................
An_Sc_eryt   .............................................................
An_Je_AM49   .............................................................
An_Ku_stu    .............................................................
An_Br_pitu   .............................................................
An_Br_AM9    .............................................................
An_Br_sini   .............................................................
An_Br_sapo   .............................................................
1_Sy_fuma    AASKAKRFYVGVASCRKCHDDPKGRNQFNVWYLLPHAKGYATLAMRESAQIAKLSGINID
1_Op_terr    NAPIKDPVYIGAEACAECHDNAATGAQYRKWRTTAHARAYENLSMPESQEITRLSGITEA
1_Pa_ferm    GAAPKKPVYVGARACGQCHDGAGMGNQYSKWLTTKHSKAYAVLSLPESVEITKRSGLRGQ
1_No_arti    NGQDKHPVYVGGAVCAECHSGPSMGHQFSKWLQSKHAQAYASLAKPEAREITELSGIPIE
1_Th_terr    APPNFQPVYVGVKVCAECHEGESLGDQCGIWAQSAHARAYAALHKPEARQIARLSGVAEE
1_Aq_sp01    QAQPRKPVFVGAKVCATCHDGPSMGHQTTLWMGTQHARAYASLAMPVARDIASISGVPVE
2_UBA5172    .............................................................
2_Tr_sava    .............................................................
3_Lu_citr    .............................................................
3_Ma_lute    .............................................................
                           Heme 1
```

An_Sc_japo

```
An_Sc_japo   .............................................................
An_Sc_eryt   .............................................................
An_Je_AM49   .............................................................
An_Ku_stu    .............................................................
An_Br_pitu   .............................................................
An_Br_AM9    .............................................................
An_Br_sini   .............................................................
An_Br_sapo   .............................................................
1_Sy_fuma    VLTSPICLGCHSTASDAENWERDKGFHLEDGVQCERCHGPGSEYSDPAIMADREAAVRAG
1_Op_terr    PLKSRMCLGCHATASDEEDWRRDDGFRLEDGMQCEMCHGPGSEYATKEIMKDREQAMKRG
1_Pa_ferm    PWKEPICLGCHSTASTAEDWEKDDAFRPEDGLQCESCHGPGSEYMSEEVMRNRAEAMKAG
1_No_arti    PQKSTLCLGCHTTGVRAEDWEKDPTFVAEDGIQCERCHGPGSEYANMDTMTDRRAAREAG
1_Th_terr    PDQSRICLGCHATAAEAEPWELDPTFSVREGVQCEKCHGPGSEYADAAIMRDRQKAMERG
1_Aq_sp01    PQKSRLCLGCHATGAEAESWETDETFSIRDGVQCEKCHGPGSEHVD.SMAPGKPKDLRIA
2_UBA5172    .............................................................
2_Tr_sava    .............................................................
3_Lu_citr    .............................................................
3_Ma_lute    .............................................................
                   Heme 2                     Heme 3
```

An_Sc_japo

```
An_Sc_japo   .............................................................
An_Sc_eryt   .............................................................
An_Je_AM49   .............................................................
An_Ku_stu    .............................................................
An_Br_pitu   .............................................................
An_Br_AM9    .............................................................
An_Br_sini   .............................................................
An_Br_sapo   .............................................................
A_Sy_fuma    LRIPDKDYCMNCHEEKGSHQSVLK.........VKPYVFETAIETISHKGRGGAVPETSK
A_Op_terr    LLMLTKDDCLRCHRPKGSHDMVLK........GRKPFNLDEAWQAIAHPIPKGKQ.....
A_Pa_ferm    LRLPNEDTCLGCHMEKGSHTAVNP.........NSTVDIKQAIPRIAHPLMKPSQLRAA.
A_No_arti    LMMPSGNDCMGCHRAKGSHDAVLG.........PNHFDLAQALQDIHHPTPDDWTVEAS.
A_Th_terr    LRMPGLEGCIYCHAPKESHQRVLG.........KSGLDIAKGWQSIKHPVPSNASLSPLE
A_Aq_sp01    LTNPLPADCMNCHKDKPSHTRMLPQKPARPNRTETPFDLVEALKAIAHPTPKDAKPAAI.
C_UBA5172    .............................................................
C_Tr_sava    .............................................................
B_Lu_citr    .............................................................
B_Ma_lute    .............................................................
                     Heme 3
```

```
An_Sc_japo

An_Sc_japo   ............................................................
An_Sc_eryt   ............................................................
An_Je_AM49   ............................................................
An_Ku_stu    ............................................................
An_Br_pitu   ............................................................
An_Br_AM9    ............................................................
An_Br_sini   ............................................................
An_Br_sapo   ............................................................
1_Sy_fuma    RSQPLLD....GPKYVGVMVCARCHNKSTTGFAFSKWRLSSHADACVTLGSDKARQIASG
1_Op_terr    ..............................................AVLAAA........
1_Pa_ferm    .PAPR......GGDFIGSSACGACHKGPESGHQWDVWRRSDHARAWAVLSTADGRRIADE
1_No_arti    MPTPDFDHASVDAMLTGSVTCAKCHEGPDQGQQFSKWTLSGHARSYATLSTPRAYEIAAE
1_Th_terr    RPAPPVDK..TQSALVGSAVCAECHDGARSGWQFSKWLESKHAGAYSCLGSTKGQQLAKK
1_Aq_sp01    GPPPLPTKEGATASYIGSHACAECHDAADKGSQFCKWRDTPHAKGYASLGTQDARDRAAK
2_UBA5172    RP..........................................................
2_Tr_sava    ............................................................
3_Lu_citr    ............................................................
3_Ma_lute    ............................................................
                                 Heme 5
```

```
An_Sc_japo

An_Sc_japo   ............................................................
An_Sc_eryt   ............................................................
An_Je_AM49   ............................................................
An_Ku_stu    ............................................................
An_Br_pitu   ............................................................
An_Br_AM9    ............................................................
An_Br_sini   ............................................................
An_Br_sapo   ............................................................
1_Sy_fuma    AGVEGDPRQAPACLKCHVTGQGEPEGPSLKTFEPMRGVQCESCHGPGSEYAREEV..MID
1_Op_terr    ...SASPPTSAQ................................................
1_Pa_ferm    MKVTGDPQLSKQCLGCHAV...........GAEANEGVGCEACHGAGKKYATEAV..MKD
1_No_arti    KGID.NPQENEDCLSCHTTAFHTPASGALDSFRLSEGVGCEACHGAGSEHVELAA..SAT
1_Th_terr    RGVEGDPRTATQCLKCHSTAYHVPAAAIRESFNLFEGVGCEACHGPGSQHVES....RRR
1_Aq_sp01    RGIKEDPQSSLECLKCHATAYSRESAGAAEGYSVLEGVGCEACHGPGSEHAEAAAALKER
2_UBA5172    ............................................................
2_Tr_sava    ............................................................
3_Lu_citr    .............................................PGRKLARAHV.....
3_Ma_lute    .............................................PGRMFVRHHV.....
                         Heme 6                      Heme 7
```

```
An_Sc_japo                                             TT     TT
                                                          30         40
An_Sc_japo   ...................................VQSAGAGFIQGT.....RVRTEHPS
An_Sc_eryt   ...................................SQSASAGFIQGT.....RVRTEHPS
An_Je_AM49   ...................................GGVASAGYIQGT.....HVKTDLPS
An_Ku_stu    ...................................GGVATAGYIQGT.....HVKTDLPG
An_Br_pitu   ...................................GGVSGAGFIQGT.....HVKTDLPG
An_Br_AM9    ...................................VGVSSAGFIQGT.....HVKTDLPG
An_Br_sini   ...................................GGVANAGFIQGT.....HVKTDMPS
An_Br_sapo   ...................................GGVASGGFIQGT.....HVKTDMPS
1_Sy_fuma    KAASRRAGLNDAPGETCLRCHNGFHGKTLEYESMWKAISHSKPDDAQ...NRQQAVEYKT
1_Op_terr    ....................................PTAAPATPPARPAPPLHVEPTYKN
1_Pa_ferm    PAAAKAAGLRIPSTTGCNHCHRETHGKTFDAAKAWTKIAHPTKVPPP.....PAEIRYKT
1_No_arti    SDDSILPRLPKATEQSCRVCHAGAHGKTFDFQTALNKIAHPKTPEAS.....RQPIRYKT
1_Th_terr    NLSQQDGSLRKPTRETCLGCHQSSPDKPFDFEQMMAKIAHPSQPDRS...TQNSEVVYKT
1_Aq_sp01    PKTFKTGLLSNP.KETCTACHGGTKEKPFALEEKWKAIAHPATPPAE.....HRVVRYKT
2_UBA5172    ...................................KPATAAEHTSSP.....EAAIRYLS
2_Tr_sava    ..............................WKDSGTVVSAATSRAQT.....GEVLEYAS
3_Lu_citr    ..NERTAVTDHP..LLCSSCHL........YTSQNKYVSK............FINEDYLS
3_Ma_lute    ..NQKTAVTDHP..LKCTSCHL........YTSKNKIVSN............LINEDYYS
                             Heme 8
```

```
               β1        β2        β3        β4        β5       β6
An_Sc_japo   ------>  TT  ------>  ------>  ------->  ------>  TT  ------>
                     50        60        70        80        90       100
An_Sc_japo   PFHVAVSPDGGQLALCNQSGHSVTFVDARSRKVTGEVPVQVQPEASVYTQDGRTLYVCNA
An_Sc_eryt   PFHVSVSPDGGTLFLANQSGHSVTFVDTRTRKVTGEVPVQVQPEATVCTQDGRTLYVCNA
An_Je_AM49   PFFVTTSPDGNTLFVVNQSGHSVTFVDTKSRKVVGEVAVQVQPEAAAPTPDGSFLYVCNA
An_Ku_stu    PFHITMSPDGSTLFISNQSGHSVTFVDARTQKVTGEVAVRVQPEASAVTPDGAFLYVCNA
An_Br_pitu   PFHITTSPDGGTLYICNQSGHSVTFVDARTQKVTGEVSVGVQPEASAMTPDNAFLFVCNA
An_Br_AM9    PFHITTSPDGGTLYICNQSGHSVTFVDARTQKVTGEVAVGVQPEAAATTPDNAFLYVCNA
An_Br_sini   PFFVTTSPDGGMLYICNQSGHSVTFVDAKTQKVVGEVAVQVQPEAAAPTPDNAFLYVCNA
An_Br_sapo   PFFITTSPDGGMLYICNQSGHSVTFVDAKTQKVVGEVAVGVQPEAAAPTPDNAFLYVCNA
1_Sy_fuma    PFNLAVTRDGRRLFVACEGSGSLIAVDTKKRAVIAETKVQNLPHGVSLSPDEERVFVSNR
1_Op_terr    PLNLALSPDNRELWVACEQAGTIAVVDTATRTKALEFQVGGQPTEIAFTPDGRRAFVSNR
1_Pa_ferm    PLNLTFRPHTAELWVTCESGNVVAIVDTVSRQKQAEIVVGNAPTDVAFTPDGKRAFVSNR
1_No_arti    PLNMAISPSGEELYVACEASDTVIVIDTETQKRVAEIAVGGQPHDVTFAPSGATAYVSNR
1_Th_terr    PVNIAVSPNFDIALVACEASNTVALLDLDTMTVRGEIPVGRQPHDVAFTPDGLYAFVSNR
1_Aq_sp01    PLRLAFRPGGKEVYVTCESAATVCVLDASSMAKVAEVAVGGQPTDVTFSPDGSLAYVTNR
2_UBA5172    PVALQLSPNGQRLYVVCEDSDAVLAVDTRSQRIVGRAEVGKRPKGIAISPDGNTLYVSDE
2_Tr_sava    PVEMLFSLSGRQLYVLCQGTGEVRVLDATTYAERKRIQVGRSPRGLSLSANGELLYVANS
3_Lu_citr    PFNLDIMNDGEKLLVVAQDANALLVVDTKSKKVLKNINVGIHPHSVIIGKEKFCYVSNQ
3_Ma_lute    PFNIAVSNDGKRLFVVAQDTGELLIVDAESKTVVQKVKVGVHPHSVILDKTNAFAYVSNQ
```

```
An_Sc_japo   η1  β7        β8          β9      TT  β10     TT  β11          
                      110         120         130         140         150         160
An_Sc_japo   ESDTVSVIDVSNKAVVKTIKVGDWPCSVKLSKDGSKAYVCCSGSMWNTVDIIDTAMNNKV
An_Sc_eryt   ESDTVSVVDVGNKAVIKTIKVGDWPCSIKLSNDGMKAYVCCSGSMWNSVDIIDTAMNNKI
An_Je_AM49   ESDSVSVIDIARKQVVKEIKVGDWPSGIKISKDGKTAYVACSGNMWNTVDVIDTGRMEKI
An_Ku_stu    ESDSVSVVDIQRKQEIKEIKVGDWPSGIKISPDGKTAYVACSGCMWNAIDVIDTGRMEKV
An_Br_pitu   ESDSVSVIDIARKQVVKEIKVGDWPSGIRITKDGRTAYVACSGCMWNTIDVIDTGKMEKT
An_Br_AM9    ESDSVSVIDVARKQVVKEIKVGDWPSGIKISRDGKTAYVACSGNMWNTVDVIDTGRMEKT
An_Br_sini   ESDSVSVIDIARKQAIKEIKVGDWPSGIKISKDGKTAYVACSGNLWNTIDVIDTGRMEKI
An_Br_sapo   ESDSVSIIDMARKQVVKEIKVGDWPSGVKISKDGKTAYVACSGCMWNTIDVIDTGRMEKI
1_Sy_fuma    GSDTVSVVDARTYKVVAAFSVGDEPHDLMSDVSGSTLYVANAGS..NDISVVSLREGREV
1_Op_terr    LDDTVSVVDVATHQVTATIEVGDEPHGVLVDREGKHLYVLNTSI..DNVSVIDLASLQEV
1_Pa_ferm    ESDTVSVIDTETRKVTSTLKVGDEPHGVLTDAQGRRLYVLNTSS..NDIWVFDVATLQFV
1_No_arti    LEDSVSEIDVRTRTVCSTFPVGDEPHGLLTDREGQTLFVLNTSS..GTVSVVDTQTRTIE
1_Th_terr    LDDSVSVVDIRTKKVVRTISVGDEPHGLLIDSQGRFLYVCNFLS..DDISVVDLKSFKVV
1_Aq_sp01    LDDSLSVIDTSARRVTATVPVGDEPHGVRTDPSGRTLFVLNTSS..DDISVLDAASLKEK
2_UBA5172    GSDAVTEIDAQTLTIRRTISVGWNPVGLVTDRAGITLYVANTLG..DDVSVIDLRTGTEK
2_Tr_sava    WDDTISVIDTKNLSVSATWPVSAEPSSVVADRTGRYIYVANRIS..NDVAVLDARTGMEE
3_Lu_citr    WSDNISVIHLKTLAVVDTIKTGNGPSGLSISNSGEYLYAVNTFG..SDVSVIDLVSKEEI
3_Ma_lute    WSDNISVVNLATSKVLDTLITGNGPSGLALSADGAKLLVVDTYG..SDLSVIDLDTKEEM
```

```
An_Sc_japo   β12         β13     TT  β14     T......T    β15          β16      TT
                      170         180         190         200         210
An_Sc_japo   GEIRTTDYGPRDIAISPDGKTGAVILDTT......GKINRGVDFVDLATGRVIEHRNIYQ
An_Sc_eryt   GEIRTSDYGPRDIAISPDGKTAAVVLDTT......GKINRCVDFIDLATGRVTETRVIPG
An_Je_AM49   RSIYTSDYGPRTLDISPDGKTLAVINDSV......GSINRSVNFIDVASGKVTEKRVIRE
An_Ku_stu    RSIYTSDYGPRMVEISPDGKTLVAILDTV......GSINRSVDFIDIASGRVVENRVIHE
An_Br_pitu   RSIYTSDYGPRTLDISPDGKTLAVINDTV......GSINRSVNFIDVDTGRVTEKRVIRE
An_Br_AM9    RAIYTSDYGPRTLDISPDGKTLAVINDTV......GSISRSVNFIDVDTSRVLEKRVIRE
An_Br_sini   RSIYTSDYGPRTLDISPDGKTIAVVNDTC......GSINRSVNFIDLATGKVTEKRVIRE
An_Br_sapo   RSIYTSDYGPRTLDMSPDGKTIAVVCDTV......GSINRSVNFIDLATGKVTEKRVIRD
1_Sy_fuma    KRL.SAGRGTWGMSRSPDNRLIYVTNNLSHFVKFRAPSRSEVTVIDTGTARVRNRIVIPE
1_Op_terr    KRL.SASRSPWDLALSPDGQTIVVTNVLSRYTGDRNPSLSELTVIDTARAVVKDRITVPG
1_Pa_ferm    KTL.SGGVGPWSLALSPNGKSIAVTSTFSNLTGFRKPLKSEITLIDTERAAVVDRPMVPG
1_No_arti    KTL.AASRNPWSLALSPDGSHVAITNNLSRFVPFRHPSQSEVTRIHTATHQIDQRDTVVG
1_Th_terr    KTL.SAGRGPWSLSLSPDGKWLVVTNIYSRFVPFRTEAQTELTILDTSTVQVVNRPVAHG
1_Aq_sp01    KRL.SASRFPWSLALSPDGGRMLVTNALSRFVKFREPSVSEITAIDPAREVIDDRPTAPG
2_UBA5172    KRL.HAGRFPEYVALSRDGSRVYVANLLARLGPPDQPPISELTVIDSATQQVTARLEVPG
2_Tr_sava    KRL.AAGRGASYITASPDASKLYVTHVYPNLTPLRTAPESEITVIDVRRAVVAERIALPY
3_Lu_citr    KRI.STGSDPTGIALSPKGKNLYVTSRRAKIAPYGDPLISELTLINDNTQRLTKQISVPS
3_Ma_lute    KRL.RTGSDPTGTALSPDGKKLYVTSRRSKIVPYGEPLVSDLTQIDATTLRVDEHKDVEE
```

```
An_Sc_japo       β17  T..T   β18         TT β19  η2     β20              TT
                   220         230         240         250         260
An_Sc_japo   SCNLRGVEYTP..DGKYVLVTMEQPKNWLPVCEAENAQIFSNNLAVVETK.......KGG
An_Sc_eryt   SANFRGVEYTP..DGQYVLFTMEQPKNWLPVCEAENAQIFSNNVAMVETK.......KGG
An_Je_AM49   SSNLRDVVYTP..DGQYIVVTYETPKNWLPVCEAENGQVFTNNIAVLETK.......PGG
An_Ku_stu    SSNLRDVVYTP..DGKYIAVTHQTPKNWLPVCEAENGQVFTNNVTIIETK.......AGG
An_Br_pitu   SANLRDVIYTP..DGQYVVVTYETPKNWLPVCEAENGQVFTNNIAVLETK.......AGG
An_Br_AM9    SSNLRDVVYTP..DGQYVVVTYETPKNWLPVCEAENGQVFTNNIAVLETK.......PGG
An_Br_sini   SSNLRDIVYTP..DGQYVVVTYETPKNWLPVCEAENGEVFTNNIAVLETK.......PGG
An_Br_sapo   SSNLRDIVYTP..DGQYVVVTYQSPKNWLPVCEAENGEVFTNNIAVLETK.......PGG
1_Sy_fuma    ANLVQGIDFSP..DGEFALVTLLRTKNLVPMVRVVQGWVITNGIGILWK........DG
1_Op_terr    ANLLEGIAWHP..SGEYAVFTELRTKNLVPMTRINHGWTITNGLGLLWA........DG
1_Pa_ferm    TNLMAGIAWHP..SGRFALATMNRTKNLVPMTRLMQGWTITNGLAVLWA........DG
1_No_arti    ANLMMGIDWHP..SGRFALATNNRSKNLVPMTRLLQGWTLTNGLAIIWA........DG
1_Th_terr    ANGLRGVAWDP..KQRFALFTLARHKNVIPITRLLQGWTITNGLGILWP........DG
1_Aq_sp01    ANMLQGIAWHP..SGAFALATLERTKNLIPMTRMVQGWTVTNGLAVIAA........DG
2_UBA5172    VLQLRHITELPAAAGGYLLIPFMRPKNLNPLVQIQQGGYLTHGMAVIRPSATSAGEARDY
2_Tr_sava    IAHGFHVTFSA..DGNLGAAAGLHPKNLPPLAHLEHGGAFADSITVFGSD.......VG
3_Lu_citr    AYLMENIAFTP..SSDLAIMTLTRPKNLVPSIQVEGGWMMTHGIGIVEQK.......EDG
3_Ma_lute    AYMMENITFTP..SGDLALMTLIRPKNLIPSIQVEGGWMMTHGIGVIDQN.......NEG
                                      HZS-β loop
```

```
An_Sc_japo   β21       TT    β22      TT  β23       β24    α1           α2
                  270         280         290         300         310         320
An_Sc_japo   KVASMPLDEHNNYDGNPYGVTVSPDGKYAFIGVRSMHRITILDLEKLLDIVVSSTQEE.L
An_Sc_eryt   KVAQFPLDELNNYDGNPYGIAICPEGKYVYIGVRGMHRVTILDLAKMTDIIQSSSQCE.L
An_Je_AM49   KVARLPLDELNNYDGNPYGLAMDPQGRYVYVGVRGMHRVTILDMNKALSIVRGNSQAE.L
An_Ku_stu    KVARLPLDDLNNYDGNPYGMAMDPKGKYLYIGVRGMHRVTILDMDKVLGLVRSSTQEE.L
An_Br_pitu   KVARLPLDELNNYDGNPYGLAMDPKGRYLYIGIRGMHRVTILDMGKVLNVVRGNSQSE.L
An_Br_AM9    KVARLPLDELNNYDGNPYGLAMDPKGRYLYIGVRGMHRVTILDMAKVLNAVRGNSQAE.L
An_Br_sini   KVARMPLDELNNYDGNPYGLAMDPKGRYLYIGVRGMHRVTILDMGKLLNIVRGNSQAE.L
An_Br_sapo   KVARLPLDDLNNYSGDPYGLAMAPKGRYWYIGIRGMHRVTILDINKVLGVVRENSQEE.L
1_Sy_fuma    RVDQLLLDEFDHHFADPTDVAITAKGRYAYVSGGGVNAVAAIDVERMKEVLNRASSEERE
1_Op_terr    TVDQVLLDQNDICFPDPTDIAITPDGRLAVVTSSSSDRVAVVDLQKLTALLKSAPPEERT
1_Pa_ferm    TVDQVMLDQPGESFSDATDVAITNDGRYALVTSSGTDRVAVVDCAKLERMLKAATPKDRE
1_No_arti    RIDQVLLDEPNLCFPDPTDVAITPDGKWALVTSSGSDRVAVVDIHRMIEMLENASDDERE
1_Th_terr    RIDQVLLDEPGECFPDPHDVVISPDGLQAFVTSAGNSRVAVVDLKKLEAVVRSYPDETRE
1_Aq_sp01    RVDQVLLDDPGESFPDPTDVAFTPDGTLAMVTSSGSDKVALIDVAKLRRVIDQATPKERE
2_UBA5172    QVSELLLDDIDHYYADGFGVASTPDGHWALVTASGSNIVSIVDTSRLRTLLLNSRPMHRL
2_Tr_sava    QPVELPLDELERYSVRPFGVAITPDKSRLFVTCGGSEEVTVIDVPRMLRYVHAHPGSH..
3_Lu_citr    KIIQLLLDEPNSYYPNPFDIVITPDGKKAFVSSSGVDYISVISMDSIKNILASTPAKK.L
3_Ma_lute    RIIQLLTDQPNAYYSDSFDIVVSPDGKRAYVSNAGADKITVMSVDSIRAIIKETPKEQ.L
```

```
An_Sc_japo        α3  β25      β26   TT   β27       β28
              330       340       350       360       370
An_Sc_japo  DDMKNDLTLMIDYLVDRVNVGLGPSSVVLSPDGKTLYTANYFTNSVSVIQV........
An_Sc_eryt  DAMKDDLTIMVDYLVTRVNVGLGPSSVALSPDGKICYAANYFSNNVSVIRTPLEMKKMF.
An_Je_AM49  DYLRDDLGLVRDYLVARVPVGLGPSSVCLSPDGKFCYAANYFSNNISVIKTPVDMRKGMI
An_Ku_stu   DYLRDDLGLVRDYLVARVPTGLGPSSVCLSPDGKFCYAANYFSNNVTVIRTAVDMKTGVV
An_Br_pitu  DYLRDDLGFVREYLVARVTTGLGPSSVCLSPDGKYCYAANYFSNNVSVIRTPVDMRSGIK
An_Br_AM9   DYLRDDLGFVRDYLVARVPTGLGPSSVCLSPDGKYCYAANYFSNNVSVIRTPVDMKSGIK
An_Br_sini  DYMRDDLGLVRDYLVARVPTGLGPSSVCLSPDGKLCYAANYFSNNVTVIRTPVNMKSSL.
An_Br_sapo  DYLRDDLGLARDYLVARVPTGLGPSSVCLSPDGKICYAANYFSNNVSVIRTPVDMKTSM.
1_Sy_fuma   SVLPNHLGVPNDYIVKRIEVGRGPRGLVASPDGRFVYVADALDDAVSIIDTA........
1_Op_terr   RVIPNHTGKPAEFVVAYIPVHTTPRGITCSADGATAFVASKLDDSVTVIDLR........
1_Pa_ferm   TVIPNHLGKSAEFLAGFVPTSTSPRGVTMGPDGRYAYVAGSLDDELTVIDLK........
1_No_arti   HVFPNHLGKPTEFVVASIPTGISPRGVVVSADGSTAYVANCLDDSVTVIDVA........
1_Th_terr   SILPNHLGPATEFVLKQIDVGHCPRGLALDKKRGLLLVANSLDDSIGIIDVQ........
1_Aq_sp01   EVLPNHLGKASEFVLARIPTGINPRGLAVAPDGKTAWVACALEDAISVIDIA........
2_UBA5172   E.LANRLDSAHKLVAARLLTGRNPTDVVTSADGRFAYIANRMDDTISVLDMT........
2_Tr_sava   ...ATDLSASANYVVARVKVGHDPRGALLARGGTKLLVANRLDDTMSVIDTR........
3_Lu_citr   KLYANTLGISKRFVIKRIKTGANPKGLKLSADGELLYIAENLEDKIGVINTK........
3_Ma_lute   KNIANNLGVSSRFVIDRISTGANPKGLALSPDGKKLYIAEQLEDKIGVLNTE........
                                                           Start HZSγ

An_Sc_japo                          β29              α4
                                  380       390       400       410
An_Sc_japo  .....................GEEPVVIATIKTGPDWEPLERGEPLTVPEVHYRVKHSPY
An_Sc_eryt  VMSLIATLCLFSLTATVKFADAGEAQVIATIQTGPEWEPLERGEPLTVPEVHYRVKHSPY
An_Je_AM49  KIGLVAALGIVGVVTAGEVM.AGTPQVIATIQTGPEWEPLPRGEPLTVPEVHYRVKHSPY
An_Ku_stu   KIGLVAALGVVGLISAGGVY.AGQPRVISTIQTGATWEPLGREEPLTVPEVHFRVKHSPF
An_Br_pitu  KIGLVAALGLFGVVTTGQLM.AGTPQVVATIQTGPEWEPLPRAEPLTVPEVHYRVKHSPY
An_Br_AM9   KIGLIAALGIMGVATTGQLM.AGTPQVVATIQTGPEWEPLPRAEPLTVPEVHYRVKHSPY
An_Br_sini  KIGLIAALGIAGVMTTGELM.AGTPQVVATIQTGPEWEPLPRGEPLTVPEVHYRVKHSPF
An_Br_sapo  KIGLIAALGIAGVVTTGEIM.AGTPQVIATIQTGPEWEPLPRGEPLTVPEVHYRVKHSPF
1_Sy_fuma   .......................KQERVGVIDLG.......................GPK
1_Op_terr   .......................QLAVAARVDLG.......................GPS
1_Pa_ferm   .......................TLQPAGRISLG.......................GPK
1_No_arti   .......................SRTATATIDLG.......................GPK
1_Th_terr   .......................SLELVKTIDLG.......................GPK
1_Aq_sp01   .......................ARKEIRRIDLG.......................GPK
2_UBA5172   .......................RLQLAATIDLG.......................GPK
2_Tr_sava   .......................TNRVTSTVPLA.......................GPR
3_Lu_citr   .......................TLEKEQSIDLG.......................GPT
3_Ma_lute   .......................TLKMETAIDLG.......................GPT
                 Signal Peptide

             η3      α5         TT      α6        β30  TT  TT   β31
An_Sc_japo      420       430       440       450       460       470
An_Sc_japo  KSELVRYGQFLFNEASWTLQGEYSCASCHYERGQTTGTIWDLGDEGWG.SWKNTKYIRGG
An_Sc_eryt  KSELVRYGQFQFNENSWTLQGEYSCASCHYERGQTTGLIWDLGDEGWG.SWKNTKYIRGG
An_Je_AM49  KSELVRYGQFLFNDASWGLQGEYACASCHYERGQTTGLIWDLGDEGWG.SWKNTKYIRGG
An_Ku_stu   KSELVRYGQFQFNDAAWSLQGSYSCASCHYERGQTTGLIWDLGDEGWG.SWKNTKYIRGG
An_Br_pitu  KSELVRYGQFQFNDASWNLQGEYSCASCHYERGQTTGLIWDLGDEGWG.SWKNTKYIRGG
An_Br_AM9   KSELVRYGQFQFNDASWGLQGEYACASCHYERGQTTGLIWDLGDEGWG.SWKNTKYIRGG
An_Br_sini  KSELVRYGQFIFNDASWGLQGEYACASCHYERGQTTGLIWDLGDEGWG.SWKNVKYIRGG
An_Br_sapo  KSELVRYGQFIFNDASWGLQGEYSCASCHYERGQTTGLIWDLGDEGWG.SWKNVKYIRGG
1_Sy_fuma   EVTLERMGERIFHSAEATYGCQFSCHSCHP.DGGIDGITYDIEPDGIGINPVDNRTLRGI
1_Op_terr   EVTMHRRGERLFHSADIAFRRQFSCSTCHP.DGHIDNLAYSIEDSGVGMNPVDNRTLRGI
1_Pa_ferm   EITLQRKGERLFHNAKISFRQQFACHSCHP.DGHVDGLAYDIEADGIGVSPVDNRTLRGI
1_No_arti   QITLARKGERLFHSADITFHRQFSCHSCHP.DGHVDGVTYDIEPDGIGISPVDNRTLRGI
1_Th_terr   IITRERLGERIFHSAKNTFHRQFSCASCHP.DGHVEGLTYDIE.DDIGVNPVDNRTLRGI
1_Aq_sp01   EITEARFGERLFNSAGIAFRRQLSCHTCHP.DGHVDGLTYDIEADGIGTAPVDNRTLRGI
2_UBA5172   QLSRIRRGQQLFFDASHCYQGQMACATCHPHEGLSDGLAWSLETPQLGRDVVENRTLYSI
2_Tr_sava   ENSVLRNGEQTFYTARYSFQGQIGCANCHI.DSTFDGLQWDLEPDGFGRDIVDNRPIEAV
3_Lu_citr   RITVARKGRRLFNNAGHTFQNQYACYTCHP.DNHEDGLVYNMAGKDMGRNVTNTQSLREI
3_Ma_lute   RITVARQGRRLFNNAGHTFQNQYACYTCHP.DNHEDGLVYNMAGKDMGRNVTNTQSLREI
                                   Heme γ1

            η4           TT TT         α7     α8          α9
An_Sc_japo       480       490       500       510       520       530
An_Sc_japo  RYLPPFRHEGFTGHPDEIVGATSSIDRVCG.RDPGFVFRSENFSPERLEALIAYIRS.LE
An_Sc_eryt  RYLPPFRHEGFTGHPDEIVGATSSIDRVCG.RDPGFVFRSENFSPERLEALIAYIRS.LE
An_Je_AM49  RYLPPFRHEGFTGHPDEIVGATSSLDRVCG.RDPGFVFRSENFSPERLESIICYIRS.LE
An_Ku_stu   RYLPPFRHEGFTGHPDEIVGATSSLDRVCG.RDPGFVFRSENFSPMRLEALICYIRA.LE
An_Br_pitu  RYLPPFRHEGFTGHPDEIVGATSSLDRVCG.RDPGFVFRSENFSPLRLEALICYIRA.LE
An_Br_AM9   RYLPPFRHEGFTGHPDEIVGATSSLDRVCG.RDPGFVFRSENFSPLRLEALICYIRA.LE
An_Br_sini  RYLPPFRHEGFTGHPDEIVGATSSLDRVCG.RDPGFVFRSENFSPERLESLICYIRA.LE
An_Br_sapo  RYLPPFRHEGFTGHPDEIVGATSALDRVCG.RDPGFVFRSENFSPERLDALICYIRA.LE
1_Sy_fuma   LDTAPFKWTG..........KNPSLSRQCGPRLAVFFTRVDPFTPDQVKALDRYICS.IP
1_Op_terr   ADTAPFKWSG..........VNPSLKRQCGARLAVFITRIQPFTPAQLEDLHDYLCS.IP
1_Pa_ferm   YDTAPFKWEG..........TNATLRRQCGPRLSVFFTRTLPFTNEELDALDYYITT.IQ
1_No_arti   LDTAPFKWEG..........TNPSLKRQCGPRLSVFFTRIEPFTPEQLEALDYYICT.IP
1_Th_terr   LDTSPFKWEG..........INPTLSRQCGTRLAVFFTRIQPFTPEELTALDYYICT.IP
1_Aq_sp01   LDTGPFKWNG..........GNLTLSRQCGPRLSVYFTRIQPYTAKELEAVDHYIST.IP
2_UBA5172   EGTEPFKWNG..........KNPDLETQDGPRTAMYIFRSQGFSPTEVEDLVAYIFS.LH
2_Tr_sava   KDTEPYKWNG..........GNPNLPTECGPRTEKFFWRAENYDDLTLTDLVVYVRS.LP
3_Lu_citr   KGTAPFKWNG..........KNQTVYKQDGIRFSTVLTRTEQFSYDDLDAITAYIKTGIK
3_Ma_lute   GDTPPFKWNG..........KNQTIYKQDGMRFSTVLTRTEQFSYDDLDAITAYIKRGIK
                 ▲                    ▲   ▲
               H/W144               C165 D168
```

```
                    α10                               α11
An_Sc_japo     TT   TT    ℓℓℓℓℓℓℓℓℓℓ                        ℓℓℓℓ      TT TT
                     540       550       560                     570       580
An_Sc_japo   FTGSPFRNDDGSLTEAQKRGWKVFSDPKVG..........CIECHPGDPKNPRALFSDAQ
An_Sc_eryt   FTGSPFRNEDGSLTAAQKRGWKVFSDPKVG..........CIECHPGDPNNPRALFSDAQ
An_Je_AM49   FTGSPFRNADGSLTEAQKRGEKLFNDPKIG..........CVECHPGESSDPKALYSDAQ
An_Ku_stu    FTGSPFRNADGSLTEAQKRGQKIFEDPKVG..........CLECHPGDPMDPRALFSDAQ
An_Br_pitu   FTGSPFRNADGSLSDAQKRGEKIFNDPNVG..........CAECHPGDVSDPKALFSDAQ
An_Br_AM9    FTGSPFRNPDGSLTDAQKRGEKIFNDPNVG..........CVECHPGDASDPKALFSDAQ
An_Br_sini   FTGSPFRNADGSLTEAAKRGEKLFNDPAVG..........CAECHPGDAMDPKALFSDAQ
An_Br_sapo   FTGSPFRTADGGLSEAQKRGEKIFNDPKVG..........CAECHPGDAMDPKALFSDAQ
1_Sy_fuma    RNPNRYRSED.RLTPAQRRGKAIFERTHTNTGEEIPVANRCDTCH......PGPYFTNRR
1_Op_terr    RPPNRYRALGQDLTEAQRRGQAIFERTRRNDGSLIPPEGRCATCH......PAPLYTDGR
1_Pa_ferm    RPPNRHHTPGEPYTPAQKRGKAIFERGTANDGRVIPLENRCVTCH......MPPYYTSRK
1_No_arti    RPPNRYRPLGAPLTEAQRRGKGVFEREKRANGSPIPKDRRCVTCH......FPPLYTDRS
1_Th_terr    LAPNRHRPVGAPLTPAQRRGRAIFERIYTNDGRLIPPENRCVTCH......PPPLYTDRR
1_Aq_sp01    RPPNRYRPLGAELTPAQRRGKAIFERTTTNDGRSIHKMRRCTTCH......FPPLYTDRE
2_UBA5172    LVRNPHLAADGHLTDAQTRGQALFFREKTNSGAPISPLQQCPTCH.....SPLTHFTSKI
2_Tr_sava    PRPNRSRPANGEFSAAQERGRALFNRTTDKFHNPIAEGNRCGYCH......SGAKGTNQK
3_Lu_citr    YPPNLMNNPSGELTESQYRGKKLFNRSIDNSGKEIPENGRCVTCH......PAPFYTNLQ
3_Ma_lute    YPPNLMYNPHGKLTESQLRGKEIYNRTKDNSGNVIPENNRCYTCH......PAPLYSNLQ
                                                       Heme γ2

             β32                   β33  α12                β34      β35     η5
An_Sc_japo   →            TT TT    →   ℓℓℓℓℓ      TT        →        →      ℓℓ
                     590       600       610       620       630       640
An_Sc_japo   THDVGTGRVGRDGFRTTPGAVFNTTVLESGVEPYSEEYDVPIIGLDLVKEFDTPTLRDIY
An_Sc_eryt   THDVGTGRVGKDGFRTTPGAVFNTTALEAGVDPYGEEYDVPIIGLDLVKEFDTPTLRDIY
An_Je_AM49   THDVGTGRVGVKGFRSTPGKVYNMKALEAGEDPYGEESDTPIIGLDLVKEFDTPTLRDIY
An_Ku_stu    THDVGTGRVGVNGFRSTPGKVFNISALEAGEDPYGVESNTPIIGLDLVKEFDTPTLRDIY
An_Br_pitu   THDVGTGRVGVKGFRSTPGKVFNLKALEAGEDPYGEESDTPIIGLDLVKEFDTPTLRDIY
An_Br_AM9    THDVGTGRVGVKGFRSTPGKVFNLKALEAGEDPYGEESDTPIIGLDLVKEFDTPTLRDIY
An_Br_sini   THDVGTGRVGVKGFRSTPGKVFNMQALNAGEDPYGEESDTPIIGLDLVKEFDTPTLRDIY
An_Br_sapo   THDVGTGRVGVKGFRSTPGKVFNIKALEAGEDPYGEESDTPIIGLDLVKEFDTPTLRDIY
1_Sy_fuma    IVDVGTRS...................................ELDTGGKFDVPQLNNIY
1_Op_terr    LHDVGTKF...................................ALDRDGKFDPPHLMNIY
1_Pa_ferm    KFDVGTKQ...................................ALDRQGTFDVPHLNNIY
1_No_arti    LHNVGTQM...................................PSDRDAEFDTPHLNNIY
1_Th_terr    SHDVGTKM...................................KLDRHGVFDTPQLLNIY
1_Aq_sp01    KHDIGSKM...................................EFDLDDKVDVPHLNNIY
2_UBA5172    QIDVGTAT...................................KYDTITAFDVPQLEGAV
2_Tr_sava    SFDVGTKR...................................ASDNTGLLDTPQLINIA
3_Lu_citr    LADVGTLS...................................ESDDPILFDTPHLNNIY
3_Ma_lute    PADVSTLA...................................VSDDSILFDTPHLGNIY

                                                   α13
An_Sc_japo   ℓ      TT                TT      TT   ℓℓℓℓℓℓℓℓℓℓℓℓℓ
                     650       660       670       680       690
An_Sc_japo   ASGTYFHDGSAQTLMNTIDNTCTTKDMHGITSHLSNQELQDLVEFMKAL...........
An_Sc_eryt   ASGTYFHDGSAETLMNTIDNTCTTKDMHGVTSHLNNQQLQDLVEFMKAL...........
An_Je_AM49   ASGTYFHDGSARTLMDTINNSVTEKDMHGRTSHLSQQELEDLVEFMKAL...........
An_Ku_stu    ASGTYFHDGGARTLMDTINNTVNDKDMHGRTSHLKQQELQDLVEYLKAL...........
An_Br_pitu   ASGTYFHDGSARLLIDTINNTVNDKDMHGRTSHLTAQEMQDLVEFLKAL...........
An_Br_AM9    ASGTYFHDGGARTLIDTINNTVNEKDMHGRTSHLTQQEMQDLVEFLKAL...........
An_Br_sini   ASGTYFHDGSARTLIDTINNTVNDKDMHGRTSHLSAQDLQDLVEFLKAL...........
An_Br_sapo   ASGTYFHDGSARTLIDTINNTVNEKDMHGRTSHLSQQDLQDLVEFLKA............
1_Sy_fuma    ENAPFLHDGRADTL.EEIWTRFNPEDRHGVTNDMTKDQLNDLIEYLKTL...........
1_Op_terr    DSAPYLHNGIARTL.EEIWTVYNPDDRHGVTNDMTKDQLNDLVEYLKTL...........
1_Pa_ferm    DSAPYLHNGMANTL.EEIWTVFNPYDKHGVTNDMTKDQLNDLIEFVKTL...........
1_No_arti    DSAPYLHNGIAPTL.EEIWTRYNPHDQHGVTNDLTKDQLNDLIEYLNTL...........
1_Th_terr    NSAPYLHNGIAPTL.EEIWTVHNPYDTHGVTNDLTKDQLNDLIEYLKTL...........
1_Aq_sp01    DSAPYMHNGIAATL.EEIWTVYNPNDTHGVTNDMTKDQLNDLIEYLKTL...........
2_UBA5172    MKPPYLHDGEALQL.EEIWTRFNPDDKHGLTSDMNKAQLNDLIEYLKTL...........
2_Tr_sava    ITGPYLHDGSAQTL.EQIWTVFNPEDKHGRTNDLTKDELNDLIEYLRTR...........
3_Lu_citr    SSAPYLHDGRAETL.EEIWTLYGEEDEHGYVNDMTKIQLNDLVAYLKSLRGPEYEKSKPE
3_Ma_lute    ASPPYLHDGRAATL.EEIWTIYGEDNKHGYVNDLTKMQLNDLIDYLKSLGGPEQED...E
                                         ▲
                                        H332

An_Sc_japo

An_Sc_japo   ..........
An_Sc_eryt   ..........
An_Je_AM49   ..........
An_Ku_stu    ..........
An_Br_pitu   ..........
An_Br_AM9    ..........
An_Br_sini   ..........
An_Br_sapo   ..........
1_Sy_fuma    ..........
1_Op_terr    ..........
1_Pa_ferm    ..........
1_No_arti    ..........
1_Th_terr    ..........
1_Aq_sp01    ..........
2_UBA5172    ..........
2_Tr_sava    ..........
3_Lu_citr    SYANVHLTNK
3_Ma_lute    GYQEQQTASF
```

Supplementary figure S8A

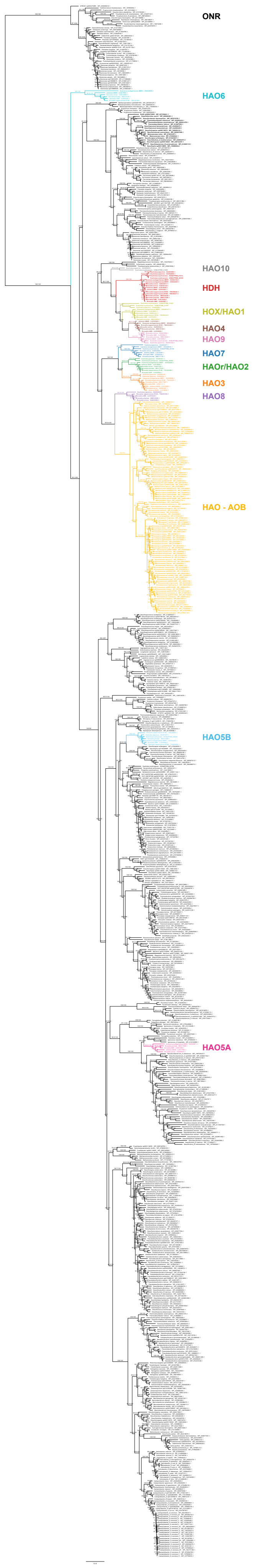

Supplementary Figure S8B

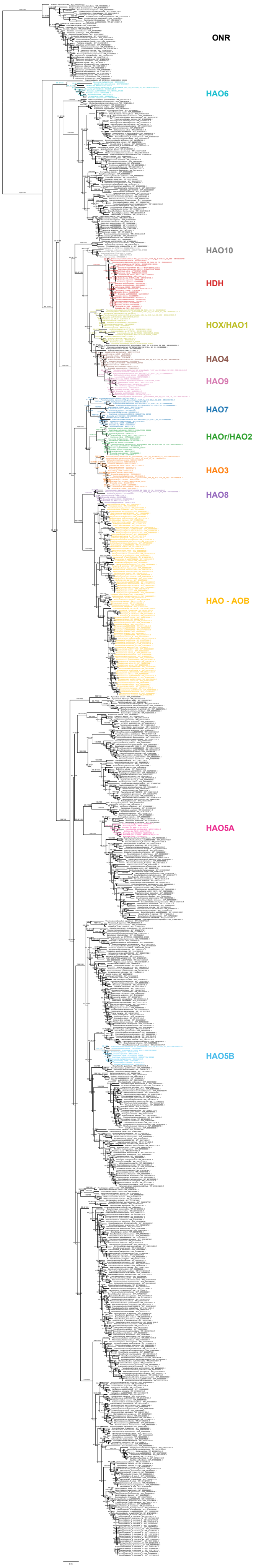

Supplementary Figure S8C

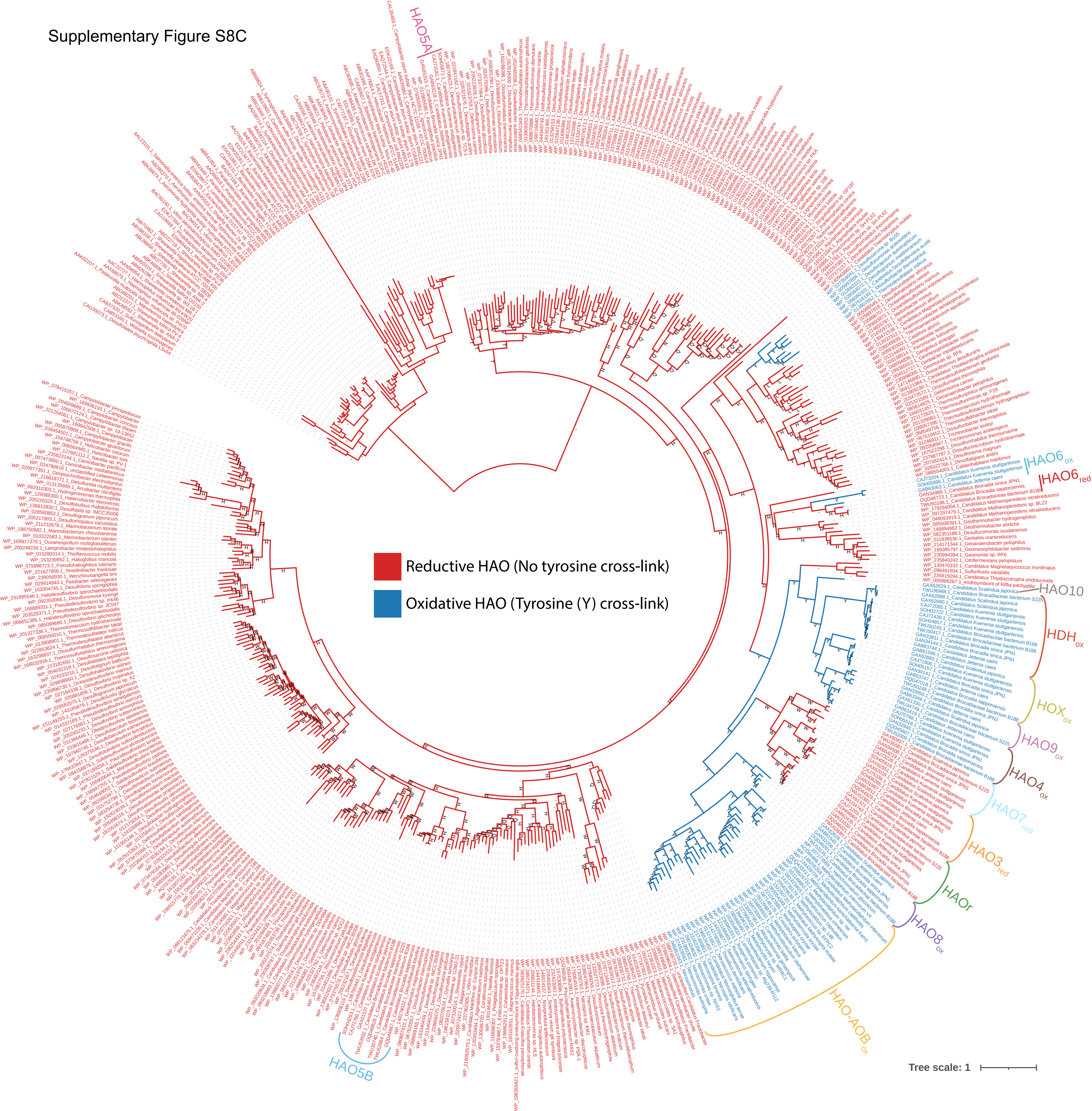

Supplementary Figure S9A

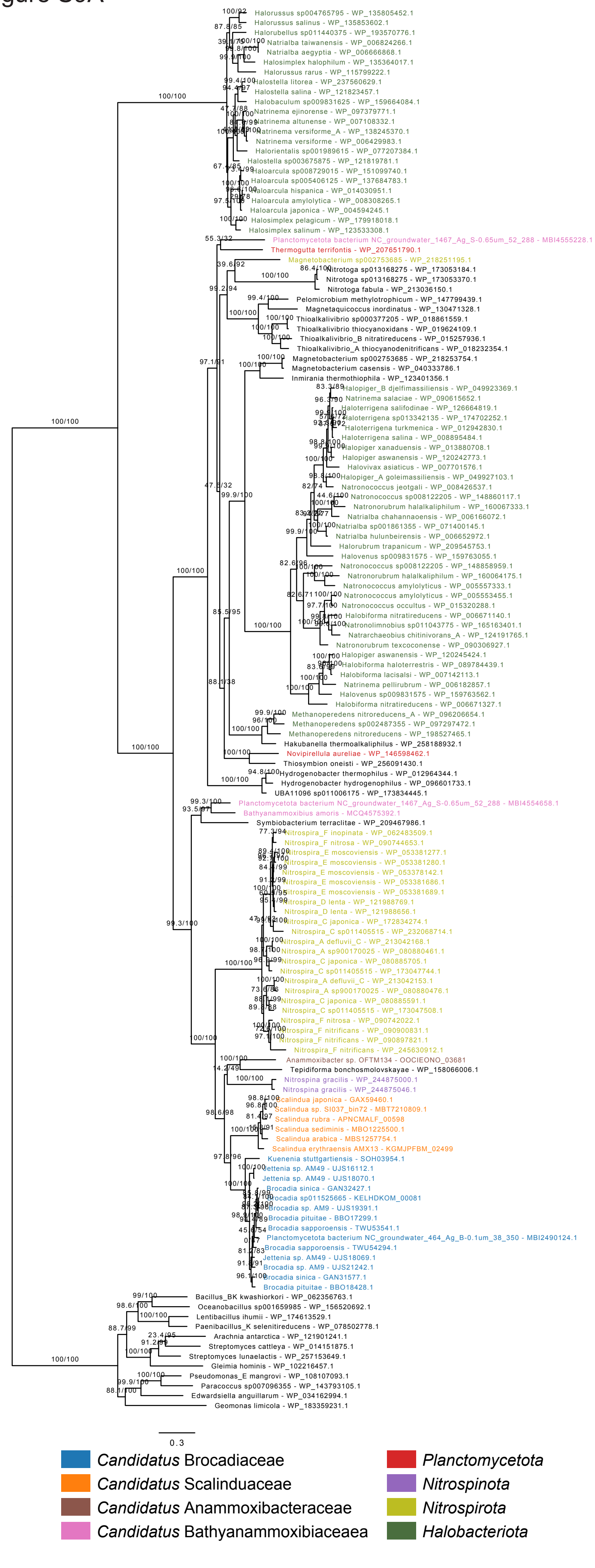

Supplementary Figure S9B

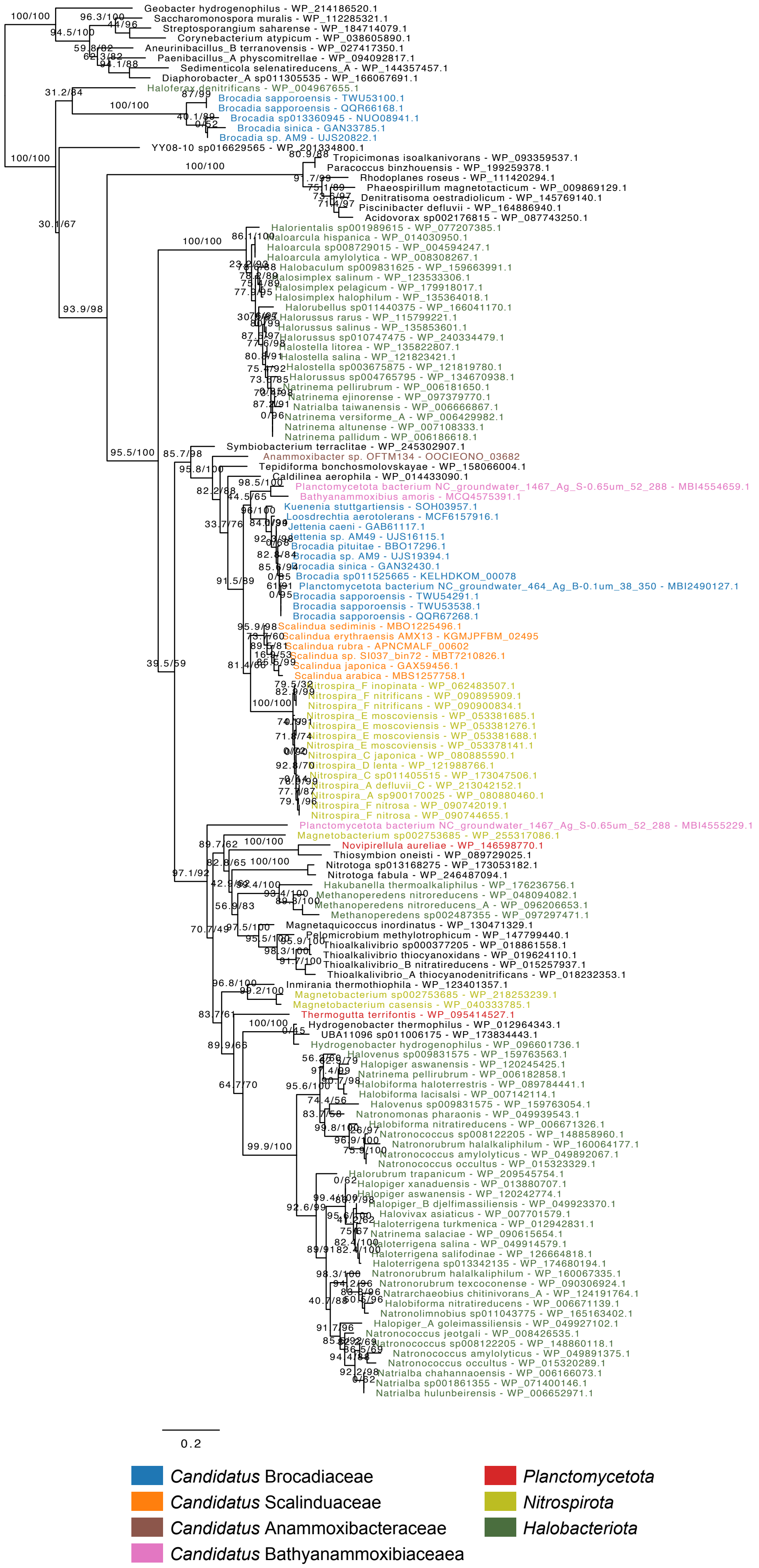

Supplementary Figure S9C

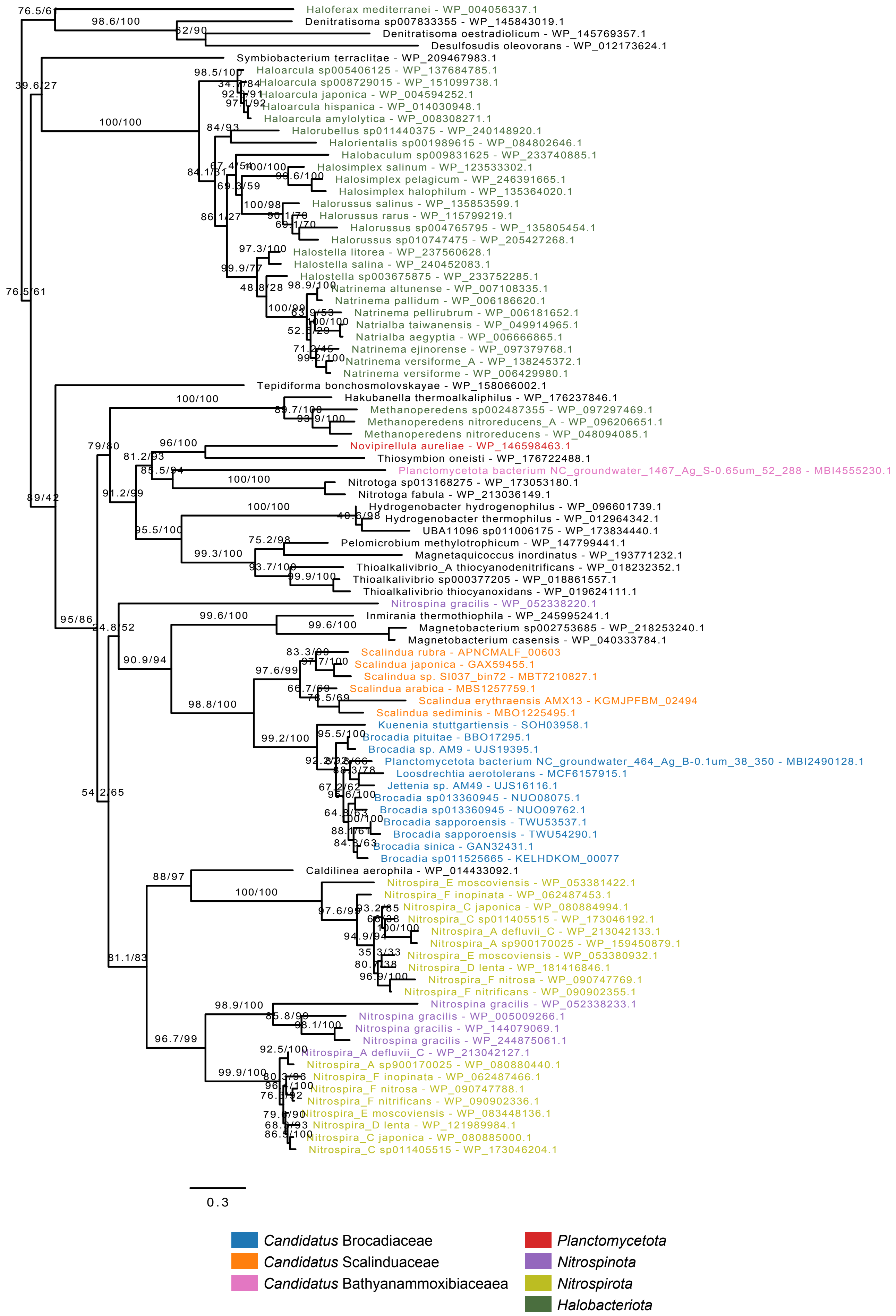

Supplementary Figure S9D

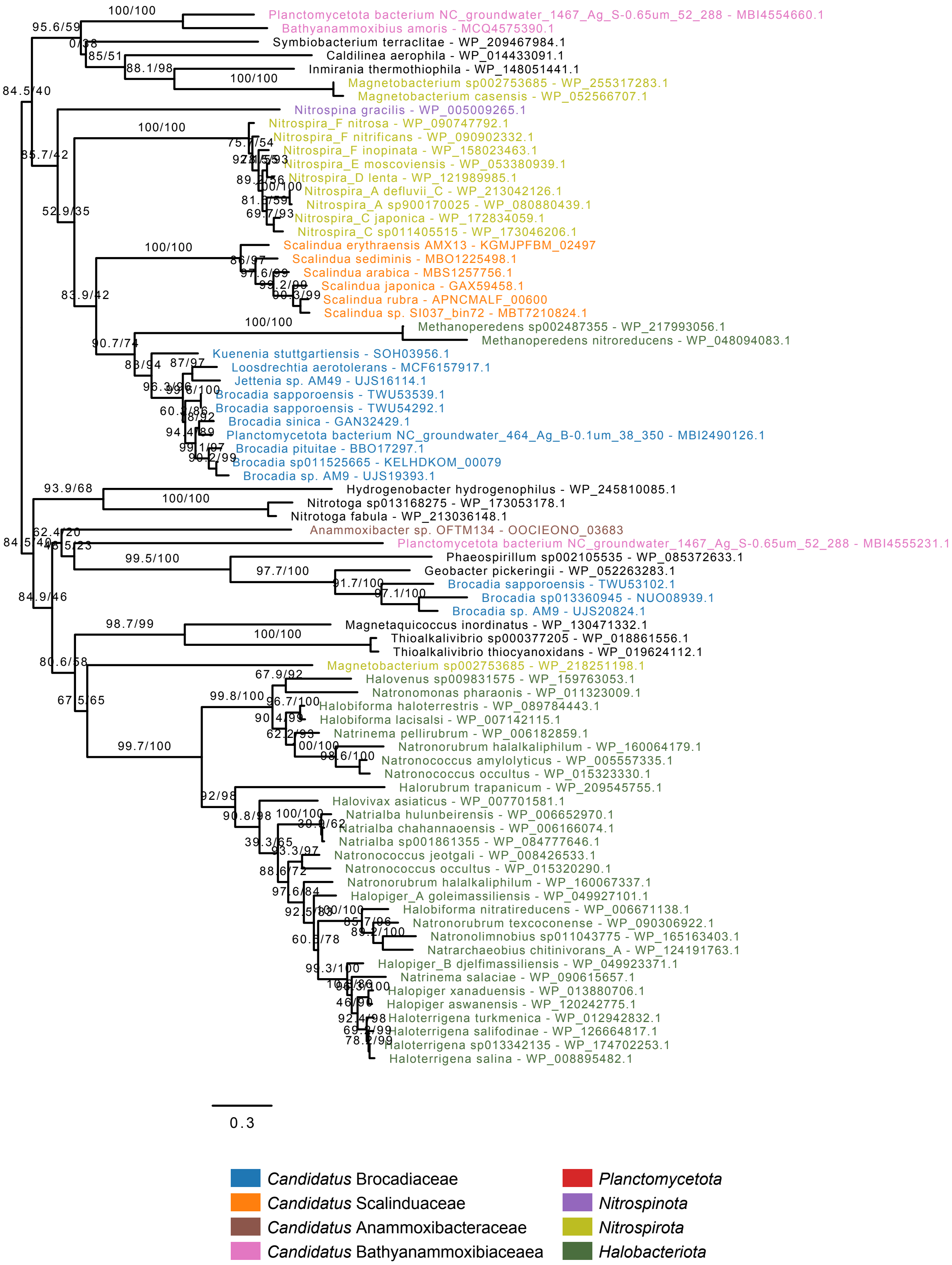

Supplement: evaf244_Supplementary_Data [file evaf244_supplementary_data.zip › SI.FiguresS1-S9.pdf]
